# Synthesis and analysis of separation networks for the recovery of intracellular chemicals generated from microbial-based conversions

**DOI:** 10.1186/s13068-017-0804-2

**Published:** 2017-05-08

**Authors:** Kirti M. Yenkie, Wenzhao Wu, Christos T. Maravelias

**Affiliations:** 1 Department of Chemical and Biological Engineering, University of Wisconsin-Madison, 1415 Engineering Drive, Madison, WI 53706-1691 USA; 2 DOE Great Lakes Bioenergy Research Center, University of Wisconsin-Madison, 1552 University Ave, Madison, WI 53726 USA

**Keywords:** Downstream separation, Physical property, Technology selection, Optimization, Cost contribution, Threshold value

## Abstract

**Background:**

Bioseparations can contribute to more than 70% in the total production cost of a bio-based chemical, and if the desired chemical is localized intracellularly, there can be additional challenges associated with its recovery. Based on the properties of the desired chemical and other components in the stream, there can be multiple feasible options for product recovery. These options are composed of several alternative technologies, performing similar tasks. The suitability of a technology for a particular chemical depends on (1) its performance parameters, such as separation efficiency; (2) cost or amount of added separating agent; (3) properties of the bioreactor effluent (e.g., biomass titer, product content); and (4) final product specifications. Our goal is to first synthesize alternative separation options and then analyze how technology selection affects the overall process economics. To achieve this, we propose an optimization-based framework that helps in identifying the critical technologies and parameters.

**Results:**

We study the separation networks for two representative classes of chemicals based on their properties. The separation network is divided into three stages: cell and product isolation (stage I), product concentration (II), and product purification and refining (III). Each stage exploits differences in specific product properties for achieving the desired product quality. The cost contribution analysis for the two cases (intracellular insoluble and intracellular soluble) reveals that stage I is the key cost contributor (>70% of the overall cost). Further analysis suggests that changes in input conditions and technology performance parameters lead to new designs primarily in stage I.

**Conclusions:**

The proposed framework provides significant insights for technology selection and assists in making informed decisions regarding technologies that should be used in combination for a given set of stream/product properties and final output specifications. Additionally, the parametric sensitivity provides an opportunity to make crucial design and selection decisions in a comprehensive and rational manner. This will prove valuable in the selection of chemicals to be produced using bioconversions (bioproducts) as well as in creating better bioseparation flow sheets for detailed economic assessment and process implementation on the commercial scale.

**Electronic supplementary material:**

The online version of this article (doi:10.1186/s13068-017-0804-2) contains supplementary material, which is available to authorized users.

## Background

Concerns regarding climate change, energy security, and petroleum costs have encouraged the search for alternative and sustainable sources of energy, fuels, and chemicals [1–3]. Significant work has been done in the bioenergy and biofuel sectors [4, 5]; however, strategies for the production of bio-based chemicals are at their infancy [6, 7]. Bio-based chemicals production has significant advantages, such as carbon neutrality and bioremediation, over traditional petrochemical routes [8–10]. Furthermore, the feedstocks involved in petrochemical processes are crude oil and natural gas. Natural gas (low cost) can contribute to the production of low-carbon content molecules such as methane, ethane, and propane. However, the production of higher carbon-containing molecules, such as butenes from natural gas, requires catalytic oligomerization which is non-trivial and cost intensive [10–12]. Typically, these high-carbon (≥4) molecules are produced from cracking of naphtha, gas oils, or from crude refinery streams [13, 14]. Since these feedstocks are quite expensive and have limited reserves when compared to natural gas, there is scope for substitution with bio-renewable feedstocks.

**Fig. 1:**
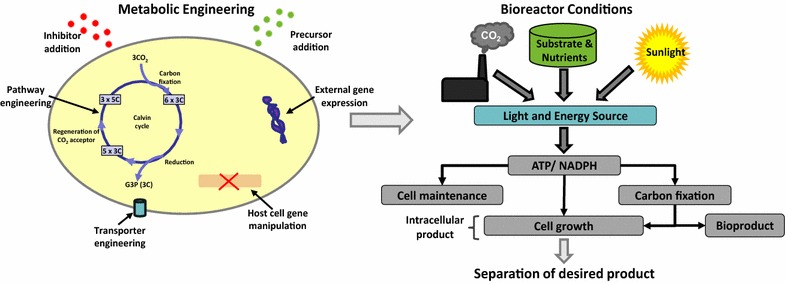
Upstream aspects for enhanced production of products of interest from microbial-based conversions. Some metabolic engineering tools and controllable bioreactor conditions are highlighted. Intracellular product is associated with cell growth, whereas extracellular product is secreted by the cells into the bioreactor effluent stream

There are several bio-renewable sources such as biodegradable wastes, dedicated energy crops, lignocellulosic biomass, and microbial cultivations [15]. Microbial hosts are advantageous as they can be engineered precisely to produce molecules of interest and the process can be controlled using different bioreactor and fermenter configurations [16]. Also, their ability to use external carbon sources other than atmospheric carbon dioxide is an added advantage [17–20]. Microbes can utilize energy in the form of light, inorganic and organic substrates for cellular growth, growth-independent cellular maintenance, and extracellular (secreted) product formation [21], as shown in Fig. 1. Cellular growth and extracellular product formation fluxes can be manipulated by metabolic engineering and/or controlled conditions [22–29]. The energy required for cellular maintenance cannot be manipulated since it is an essential function for cell survival.

Intracellular chemicals produced in microbial cultivations are usually energy or carbon storage compounds [30]. They have diverse functions and properties which render them suitable for several industrial and specialty applications; examples include biopolymers [31] and bio-lubricants [32]. The knowledge about molecular reactions and cellular level synthesis pathways has made it possible to alter the concentration, properties, and structures of intracellular constituents to products of interest [33–38]. Continuous developments and improvements in the field of metabolic engineering have generated hope that the commercialization of bio-based chemicals can be made economically feasible and profitable. Metabolic engineering tools (Fig. 1) include pathway and enzyme engineering to create synthetic pathways, substrate utilization engineering, transporter engineering, byproduct elimination and precursor enrichment, and rerouting pathways [23, 39–41]. Some examples of industrial production of biopolymers after the application of the formerly mentioned techniques are Biopol (P(3HB-*co*-3HV)) produced by ICI, Zeneca and Monsanto [42], Nodax (short- and medium-chain length PHAs) by P&G and MHG [43], and P(3HB-*co*-3HHX) by Kaneka Corporation [44].

The selection of microbial cultivation method and increase in the product content in the cell are two important aspects of bioprocess development. Equally important, if not more, is the recovery of bioproducts in desired form and purity economically. Usually, product content is very low in the (bio)reactor effluent streams (less than 20 wt%) [45], and hence separation cost can amount to 60–80% of the overall production cost [46–49]. Also, genetic modifications and growth conditions can result in the formation of complex streams containing constituents which can introduce additional challenges in product recovery. For example, selection of a suboptimal microbial strain enables faster initial progress in terms of product titer, rate, and yield (TRY) enhancement, but results in the formation of co-products which might be difficult to isolate later [6]. Hence, designing of the overall process in terms of upstream considerations, resulting effluent (also addressed as process stream) stream as well as the commercial-scale separation process, should be considered from the very beginning while developing new processes [50, 51].

Previous work on economic assessment for recovery of intracellular metabolites has been restricted to specific examples (details in Additional file 1) on storage polymers such as PHAs [52, 53], cyanophycin [54, 55], and pigments such as astaxanthin [56–58] and β-phycoerythrin [59]. Furthermore, assessment studies have been performed for individual separation technologies [60–63] and some guidelines have been suggested regarding their applicability to microalgae harvesting [64] and biofuel production [45]. However, a systematic framework for quantitative analysis and selection of technology alternatives is not available. Traditional analyses have usually focused on sensitivity studies where the technologies in the separation network are already fixed and one parameter is varied at a time to analyze its effect on the process economics [65–67]. However, technology selection is not trivial since many competing alternatives are often available. Hence, researchers have suggested alternative methods such as generation of schemes and superstructures for the synthesis of separation networks [48, 68–76]. A separation scheme [77] incorporates a list of technologies for product isolation from a mixture of components. A separation superstructure [74, 78] is a network-based representation of all potentially useful technologies and the interconnections among them, and it is used as a basis for the formulation of optimization models.

A separation scheme [77] starts with the effluent of a (bio)reactor and enables the generation of a separation superstructure, which can be used for systematic process synthesis of separation processes [74, 78–81]. To come up with systematic guidelines for designing separation networks in the future, the key objectives of this work are to:

- develop a methodology for the assessment of separation technologies performing similar tasks and analyze the effect of technology selection on overall process economics;
- include the complete separation network while performing the economic assessment so as to understand the combination of technologies in a selected network and the cost-intensive tasks;
- analyze the change in process cost with change in important process parameters such as biomass titer, cellular product content, product purity, and technology performance, based on their relative impact in the separation network selection; and
- determine the critical values of the selected parameters and understand the shifts in technology selection for designing efficient separation processes in the future.

In the "Methods" section, we present the proposed analysis framework which includes formulation of separation networks, modeling, and solution strategy. In the "Results and discussion" section, we present two case studies for intracellular products; the first study represents intracellular insoluble products, while the second study represents intracellular soluble products. We propose a base case for each and then discuss technology selection and important process considerations. This is followed by further analysis and results for selected parameters in each product class. Toward the end, we present some key conclusions drawn from this work.

## Methods

In this section, we discuss the stage-wise separation scheme and specific classes of intracellular products; the superstructure generation and solution method for these product classes; and the analysis framework for the assessment of separation technologies.

### Stage-wise separation scheme

**Fig. 2:**
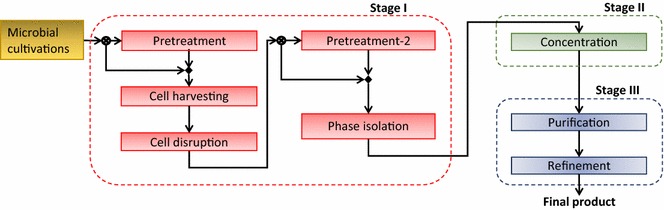
Representation of the three-stage separation scheme for intracellular products. The process streams and tasks are shown inside the *boxes* representing each stage. In stage I, the tasks of cell harvesting, cell disruption, and phase isolation are essential and must be performed in series, while pretreatment is optional and can be performed before cell harvesting or phase isolation tasks. The task in stage II is product concentration which may comprise single or multiple technologies. In stage III, purification and refinement tasks can be accomplished by either a single or combination of technology options based on the features of the input stream and final product specifications

The recovery of an intracellular bioproduct is divided into three stages: (I) Cell and product isolation, (II) Product concentration, and (III) Product purification and refinement. Each stage can have multiple technologies for performing similar tasks. Sometimes, more than one technology needs to be selected in a stage and sometimes a stage can be skipped, based on product properties, initial stream concentration, and desired recovery and purity constraints. In Fig. 2, we present a schematic of the three-stage separation framework for intracellular products. The different technologies applicable for each task are listed in Table 1 [72, 79, 82–95].

**Table 1:** Technology options available for performing the tasks listed in three separation stages

Tasks	Technologies
Pretreatment	Flocculation (Flc), coagulation (Cog)
Cell harvesting	Sedimentation (Sdm), filtration (Ftt), centrifugation (Cnt), flotation (Flt), microfiltration (MF)
Cell disruption	Bead mill (Bml), chemical lysis (Chy), enzyme lysis (Ely), homogenization (Hph)
Phase isolation: cell component separation	Sedimentation (Sdm), filtration (Ftt), centrifugation (Cnt), flotation (Flt), membranes (Mbr- MF (microfiltration), UF (ultrafiltration), and RO (reverse osmosis))
Phase isolation: product phase formation	Differential digestion (Ddg), solubilization (Slb)
Product concentration	Extraction (Ext), ATPE, evaporation (Evp), precipitation (Prc), membranes (MF, UF, NF (nanofiltration), RO), distillation (Dst), adsorption (Ads)
Product purification	Chromatography (Chr), crystallization (Crs), pervaporation (Pvp), membranes (Mbr-MF, UF, NF, RO)
Product refinement	Drying (Dry), bleaching (Blc)

*ATPE* aqueous two-phase extraction

### Intracellular product classes

The technology options available for various tasks listed in the three-stage separation scheme can be narrowed down depending on other distinguishing product properties such as the product’s solubility in water [insoluble (NSL) or soluble (SOL)], physical state [solid (SLD) or liquid (LQD)], density with respect to water [heavy (HV) or light (LT)], relative volatility with respect to water [volatile (VOL) and non-volatile (NVL)] for soluble products, and intended use [commodity (CMD) or specialty (SPC)]. Thus, intracellular chemicals can be categorized into specific product classes based on their properties. Such classification helps in identifying the relevant tasks and technology options in each stage of the separation scheme.

### Superstructure generation and solution method

The potential separation stages and the relevant technology options can be reduced using additional product properties (discussed earlier in intracellular product classes). Hence, building upon the previous work on separation schemes [96] and superstructure-based synthesis of separation networks [97], we generate an appropriate separation superstructure for each class of product. The next steps are formulation of a superstructure optimization model, solution to identify the optimal separation network design, and economic assessment.

The optimization model is formulated as a mixed-integer non-linear programming (MINLP) problem, with binary variables denoting the active (1) and inactive (0) states of technologies present in the separation superstructure. The objective is to minimize the ‘overall process cost,’ which comprises feed, annualized capital, materials, consumables, labor, utility, and other costs (e.g., supervisory and overhead cost) [98]. The optimization model is formulated in GAMS 24.4.6 environment and solved using BARON [99], a global optimization solver.

### Analysis framework

After generating a separation superstructure for a product class, we have multiple technologies which can perform the same task and each technology has a performance metric which indicates its suitability over other parallel technologies. The MINLP optimization model comprises separation technology models, stream flows, and product recovery and purity constraints. We formulate a base case using reference values for parameters such as input conditions, technology efficiencies, material cost, and requirements, and solve the optimization problem to identify the key cost drivers. However, since these reference parameters affect the process economics, we perform additional analysis to study how variations in the values of the aforementioned parameters impact the overall process cost and technology selection.

The steps involved in the proposed analysis framework are the following:

*Step#1* Formulate a base case and solve it to determine the optimal separation network and the key cost contributors. Also, determine alternate (next best) configurations, to decide which technologies are essential and which can be changed in the optimal design with little compromise in the process cost.

*Step#2* Vary a combination of parameters for the key cost contributing technologies (i.e., solve multiple optimization problems with varying parameter values) to determine the critical values when there is a shift in technology selection.

*Step#3* Extend the analysis to other product classes based on (1) the results for the representative case, if the same technologies are available for the other classes, or (2) the literature, individual technology considerations, and simulation tools [100], if new technologies should be considered.

## Results and discussion

### Study 1: intracellular insoluble products

Intracellular (IN) insoluble (NSL) products can be further classified as solid (SLD)/liquid (LQD), heavy (HV)/light (LT), and commodity (CMD)/specialty (SPC). Based on these, we list the product classes and the corresponding separation options in Table 2. The difference between commodity and specialty products is their required purity grade and hence similar technologies are applicable to both, but the latter may require more than one purification and/or refining step to achieve the desired purity.

**Table 2:** Product classes for intracellular and insoluble type of product

Product classes	Product examples	Stage I technologies	Stage II technologies	Stage III technologies	References
IN NSL SLD HV (CMD/SPC)	PHAs, Cyanophycin	Flc, Sdm, Ftt, Cnt, Ely, Ahy, Bml, Mbr-MF/RO, Ddg, Slb	Mbr-all, Cnt, Prc, Ads	Mbr-all, Dry, Blc	[53–55, 101]
IN NSL LQD HV (CMD/SPC)	Membrane proteins	Flc, Sdm, Ftt, Cnt, Ely, Ahy, Bml, Mbr-MF/RO, Slb	Mbr-all, Cnt, Ads	Mbr-all, Chr, Blc	[102]
IN NSL SLD LT (CMD/SPC)	β-carotene	Flc, Sdm, Ftt, Cnt, Ely, Ahy, Bml, Mbr-MF/RO, Ddg, Slb	Mbr-all, Cnt, Prc, Ads	Mbr-all, Dry, Blc	[103]
IN NSL LQD LT (CMD/SPC)	Microalgal oils	Flc, Sdm, Flt, Ftt, Cnt, Ely, Ahy, Bml, Mbr-MF/RO, Slb	Mbr-all, Cnt, Ads	Mbr-all, Chr, Blc	[104, 105]

*PHAs* polyhydroxyalkanoates

*Mbr-all* is inclusive of all types of membrane technologies (MF, UF, RO, NF)

Full forms of the technology abbreviations used are listed earlier in Table 1

We choose intracellular (IN), insoluble (NSL), solid (SLD), heavy (HV), commodity (CMD) product as a representative class for most intracellular insoluble products. The proposed analysis framework is applied to the aforementioned product class.

### Separation superstructure for IN NSL SLD HV CMD product

**Fig. 3:**
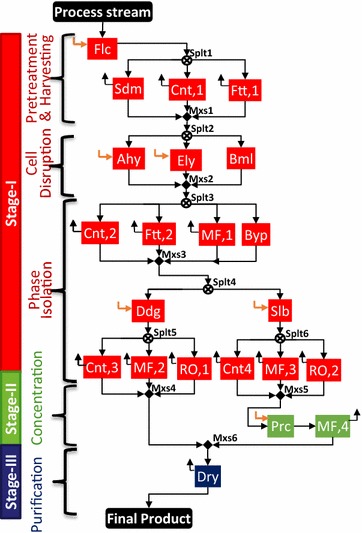
Separation superstructure for intracellular (IN) insoluble (NSL) solid (SLD) heavy (HV) commodity (CMD) product. It consists of three stages distinguished using *different colors*: (I) cell and product isolation: *red*; (II) product concentration: *green*; and (III) product purification and refinement: *blue*. The technologies involved are flocculation (Flc), sedimentation (Sdm), centrifugation (Cnt,1,2,3,4), filtration (Ftt,1,2), acid hydrolysis (Ahy), enzyme lysis (Ely), bead mill (Bml), membrane processes [microfiltration (MF,1,2,3,4) and reverse osmosis (RO,1,2)], differential digestion (Ddg), solubilization (Slb), precipitation (Prc), and drying (Dry). An option for bypassing (Byp) a set of parallel technologies is included in stage I

We start with the general separation scheme for an intracellular insoluble product (refer Additional file 1) and simplify it to suit the specific class of IN NSL SLD HV CMD product. This simplified scheme is used to generate a separation superstructure as illustrated in Fig. 3. Since the product is intracellular, we have four tasks in stage I: (1) pretreatment, (2) cell harvesting, (3) cell disruption, and (4) phase isolation. Pretreatment is optional and can be used to increase the effective size of the cells through flocculation. Cell harvesting is used to separate the cells from water present in the bioreactor effluent stream. Cell disruption releases the desired product along with other non-product cellular materials (NPCM) such as proteins, carbohydrates, lipids, nucleic acids, and cell debris in the resultant stream [101, 106]. Thus, the phase isolation task for this class may require multiple steps. One of the steps is product-rich phase formation that can be accomplished by two alternatives: differential digestion and solubilization.

Differential digestion uses an agent for NPCM digestion leaving the product unaffected in the process [52, 107], while solubilization [108, 109] uses a solvent which can selectively dissolve the product, leaving NPCM as is in the stream. Isolation of product using differential digestion already achieves substantial product concentration, and thus stage II is not required. However, if the product has been isolated using solubilization, then stage II is required to recover the product by precipitation using an anti-solvent. This is followed by membrane separation (microfiltration) to separate the precipitated product from the liquid phase. The product obtained after the two separation stages may still contain small amounts of water, acids, solvents, and anti-solvent. Drying in stage III can remove these traces and achieve the desired dry solid state with required purity specifications for the product.

Some important input parameters essential to comprehend the cost contribution and further analysis are provided in Table 3.

**Table 3:** Important input parameters for the base case for IN NSL SLD HV CMD product

Parameter	Nominal value	Units
Initial cell titer	5	g/L (kg/m^3^)
Product content in cells	25	wt% of cell dry weight (CDW)
Desired production capacity	1000	kg/h
Annual operation time	330	Days/year
Final product purity	95	wt% purity

### Cost contribution analysis

The objective is to ‘minimize the overall process cost.’ Details regarding the input conditions and technology parameters for the base case and the MINLP problem formulation are discussed in Additional file 1.

#### Optimal configuration

**Fig. 4:**
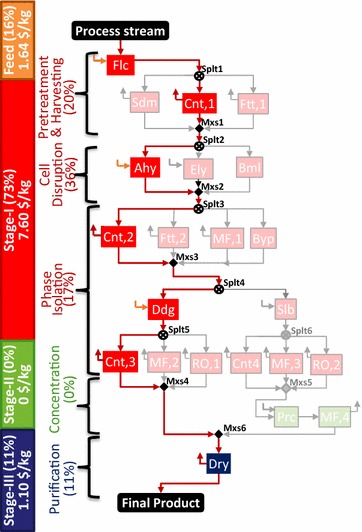
Technologies selected in three separation stages for IN NSL SLD HV CMD product. The active streams are shown by *bold red lines* and selected technologies are highlighted in *different colors* corresponding to each stage: *red* for stage I, *green* for stage II, and *blue* for stage III. Cost contribution shown by the *numbers* on the* left bar* indicates stage I to be the key cost driver, followed by feed cost and stage III. Stage II is absent in the optimal network

The base case optimal configuration and cost contributions are presented in Fig. 4. The technologies selected in stage I include flocculation (Flc) for pretreatment, centrifugation (Cnt,1) for cell harvesting, acid hydrolysis (Ahy) for cell disruption, centrifugation (Cnt,2) for initial phase separation, and differential digestion (Ddg) for product-rich phase formation, followed by centrifugation (Cnt,3) to separate the solid product from the digested NPCM and other liquid phase components. Stage II is bypassed because of the relative product concentration already achieved in stage I. The final product refinement in stage III is achieved by drying (Dry). The cost contribution shown in Fig. 4 reveals that stage I is the key cost driver (73%). The relative contribution by different tasks in the separation process is also presented. The overall process cost is $10.34/kg product where the separation cost contribution is $8.69/kg (~84%). The separation cost is a summation of the annualized capital, materials, consumables, utilities, labor, and other costs (refer Additional file 1).

#### Alternate configurations

To determine the alternate (next best) separation configurations, we add successive integer cuts [110] as additional constraints in our model (see Additional file 1). In Table 4, we present the next three alternate configurations along with the overall process cost and separation cost.

**Table 4:** Alternate separation configurations for study 1 using successive integer cuts

Configuration #	Technologies selected	Overall cost ($/kg) (% increase)	Separation cost ($/kg)
Best case	Flc, Cnt1, Ahy, Cnt2, Ddg, Cnt3, Dry	10.34 (NA)	8.69
2nd best	Flc, Cnt1, Ahy, Cnt2, Slb, Cnt4, Prc, MF4, Dry	10.69 (3.38%)	9.05
3rd best	Flc, Cnt1, Ely, Cnt2, Ddg, Cnt3, Dry	10.96 (5.99%)	9.41
4th best	Flc, Cnt1, Ahy, Cnt2, Slb, MF3, Prc, MF4, Dry	11.18 (8.12%)	9.36

### Important process considerations

Based on the base case cost contribution analysis, some key process parameters are identified. Changes in the values of these parameters have the potential of affecting the optimal separation network design as well as process economics. The details of the selected parameters and their probable range of variation are presented in Table 5.

**Table 5:** Parameters selected for analysis in study 1

Separation stage and task	Parameter	Base case (nominal)	Range	References
Stage I: input parameter	Biomass titer in input process stream	5 g/L	(0.1–200)	[45, 118–120]
Stage I: task—cell harvesting	Cell separation efficiency for centrifugation (Cnt,1)	80%	(70–95)	[64, 121]
	Cell retention factor for filtration (Ftt,1)	80%	(70–95)	[85]
Stage I: task—cell disruption	% Release for acid hydrolysis (Ahy)	85%	(80–98)	[122]
	% Release for bead mill (Bml)	85%	(80–98)	[123]
	% Release for enzyme lysis (Ely)	90%	(85–98)	[124]
Stage I: task—phase isolation	Amount of agent required for differential digestion (Ddg)	0.4 kg/kg other cell components	(0.05–3)	[109]
	Amount of solvent required for solubilization (Slb)	0.4 kg/kg product	(0.05–3)	[107]

*Biomass titer* in the feed entering the separation network is a parameter dependent on the microbial strain, cultivation route, substrate utilization, and bioreactor design. It has a potential to be altered by upstream features such as metabolic engineering tools [23, 111]. For example, microbial strains can be engineered to enhance the accumulation of desired product inside the cells [112–114]. Additionally, suitable microbial hosts that can tolerate stress conditions such as product or co-product toxicity, and growth inhibitors can achieve high product yields and biomass titers [115]. Thus, biomass titer can differ for different microbial strains and product systems, and hence it is selected for further analysis.

*Cell harvesting technologies* The performance for sedimentation and centrifugation is defined in terms of ‘efficiency’ of the separation of cells from the aqueous phase. For filtration, it is defined as the ‘retention factor’ of cells on the retentate side of the filter. It is dependent on cell size, relative cell concentration in the process stream, and properties such as hydrophobicity, relative density, and shear susceptibility [85, 86, 116]. For example, a larger sized microbial cell may be separated effectively by ‘filtration,’ while a denser cell may be separated by simple ‘gravity sedimentation.’ For non-Newtonian flows (cell concentration >15%), the performance of sedimentation and centrifugation will be affected negatively as they both are dependent on viscosity, but filtration and membranes might perform better. The efficiency of sedimentation is dependent on Stokes’ law [117], proportional to gravitational acceleration and the square of particle diameter, and inversely proportional to viscosity. These are properties of the components in the system, and hence there is limited scope for performance enhancement when compared to centrifugation and filtration options [64]. Thus, efficiency of sedimentation (Sdm,1) is kept fixed at 70% (base case) and the values for centrifugation (Cnt,1) and filtration (Ftt,1) options are varied in the selected range (Table 5) for analysis.

*Cell disruption technologies* After cell harvesting, the subsequent step is cell disruption. The concentration of cells in the stream entering the cell disruption technology has a considerable effect on the overall cost as well as product release. Bead mill is a mechanical method for cell disruption and does not introduce any secondary agents in the system. However, the properties like cell wall thickness, cell size, and shear susceptibility can change the product release efficiency [64, 124, 125]. Chemical and enzymatic lyses are other cell disruption methods which have gained popularity due to higher product release efficiency, selectivity, and low energy requirements. However, these methods introduce other components in the system, which increases the amount of materials handled downstream. The potential to recover and recycle the enzymes and chemicals in these methods is an important issue which needs further research. Enzymatic lysis can result in smaller cell debris particles which can be difficult to isolate later. All these parameters and considerations can alter the product release efficiency of the cell disruption technologies. Hence, in the third analysis, we choose the performance of cell disruption technologies, defined in terms of the percentage release of intracellular components for the bead mill (Bml), acid hydrolysis (Ahy), and enzyme lysis (Ely).

*Phase isolation technologies* The performance of differential digestion and solubilization is influenced by the amounts and costs of digestion agent or solubilizing solvent added [52, 107, 108]. Thus, we select the materials added for these parallel technologies for further analysis. Stage II is absent for the base case and stage III contributes 11% in the overall cost. Consequently, we do not perform additional analysis for stage II and stage III parameters for this product class.

### Analysis and results for IN NSL SLD HV CMD product

The results from the proposed framework are presented and some insights regarding shifts in technology selection and changes in optimal separation network design are provided.

#### Biomass titer

We vary the biomass titer in the range of 0.5–200 g/L as it includes the range for intracellular insoluble products in photoautotrophic (0.5–10 g/L) [118, 120] as well as heterotrophic (10–200 g/L) [53, 119] conditions.

**Fig. 5:**
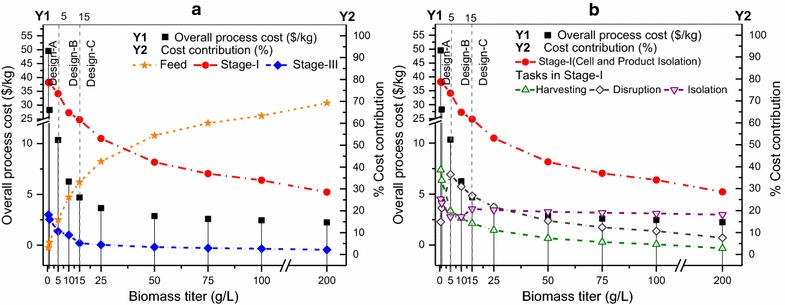
Overall process cost and contributions by feed, separation stages, and tasks with varying biomass titer. **a** Overall process cost (Y1) and cost contribution (Y2) by feed, and active separation stages I and III. **b** Cost contribution (Y2) by the three tasks in stage I: cell harvesting, cell disruption, and product isolation. The *vertical dotted lines* at 5 and 15 g/L represent the change in optimal separation network design

In Fig. 5, we show the variation in the overall process cost (axis Y1) and cost contributions (axis Y2) for feed, separation stages (Fig. 5a) and three important tasks of cell harvesting, cell disruption, and product isolation in stage I (Fig. 5b). We observe that the optimal separation network design changes with the change in titer values. When the titer is less than 5 g/L, design A is optimal; from 5 to less than 15 g/L, design B is optimal; and from 15 to 200 g/L, design C is optimal.

**Fig. 6:**
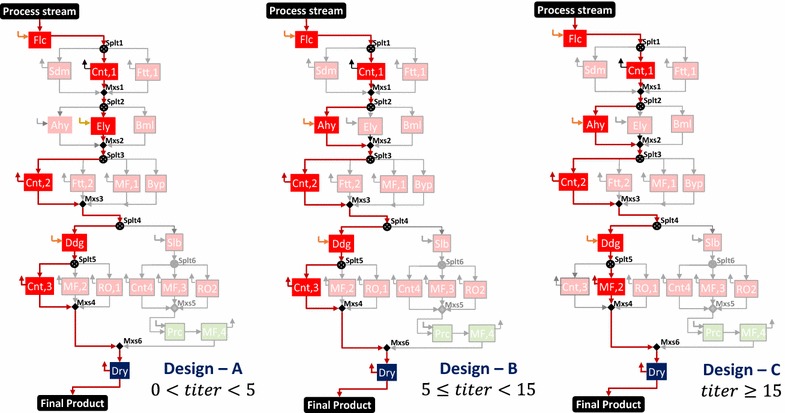
Selected technologies and active streams in designs* A*,* B*, and* C* for IN NSL SLD HV CMD product. Design* A* is optimal for titers less than 5 g/L, design* B* is optimal for titers in the range of 5 (base case) to less than 15 g/L, and design* C* is optimal for titers 15 g/L and greater

The selected technologies and active streams are highlighted in Fig. 6. For lower biomass titers (design A), enzyme lysis (Ely) proves to be the optimal choice as compared to acid hydrolysis (Ahy) in the base case (design B) because the enzyme required for disruption depends on the amount of biomass [124, 125], while acid is added to maintain a certain normality in the aqueous phase [122]. For titer values of 15 and greater, the microfiltration (MF,2) option is selected after differential digestion (Ddg) as compared to centrifugation (Cnt,3) in the base case (design B) because microfiltration (MF,2) has a better retention and concentration factor as compared to centrifugation (Cnt,3). Along with the changes in optimal network design, we also observe changes in the dominant cost drivers (Fig. 5b). For titers less than 2.5 g/L, cell harvesting is the major cost contributor because of the high utility and capital costs of centrifugation (due to the large incoming flow). For titers between 2.5 and 30 g/L, cell disruption is the major contributor as the amount of acid needed for cell disruption is high. For titers greater than 30 g/L, cell harvesting and disruption costs are low, so phase isolation becomes the major contributor as differential digestion (high digestion agent cost) is followed by microfiltration (high consumable cost).

#### Cell harvesting technologies

The second analysis is performed by varying the cell separation efficiency for centrifugation and cell retention factor for filtration [64, 85, 121]. During this analysis, we assume that the sedimentation efficiency is constant at 70% since there is not much scope for efficiency improvement.

**Fig. 7:**
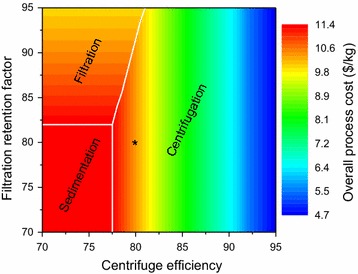
Overall process cost with variation in performance of cell harvesting technologies. The *contour lines* denote the viable region for the three technologies available for cell harvesting: centrifugation, filtration, and sedimentation. They are *horizontal* in the region where filtration is selected, whereas they are *vertical* where centrifugation is selected. The *constant color rectangular* region [(70,70), (70,82), (77.5,82), (77.5,70)] denotes the selection of sedimentation. Critical values, when there is a change in technology selection from centrifugation to filtration or sedimentation, are shown by *white lines*

In Fig. 7, the overall process cost per kilogram of product is shown as a function of the centrifuge efficiency and filtration retention factor. The black asterisk corresponds to the base case (centrifuge efficiency: 80% and filtration retention factor: 80%). The results show that centrifugation is the preferred technology for biomass harvesting in most cases (corresponding region shown by the vertical contour lines). Filtration gets selected when the retention factor is greater than 80% and the corresponding centrifuge efficiency is less than 76%. Sedimentation is selected when centrifuge efficiency is less than 77.5%, and filtration retention factor is less than 82%.

Centrifugation is the preferred technology in most cases because its major cost contributor is utility, which is lower than the large capital cost of the sedimentation tank and the high consumable replacement cost of the filtration membrane. Also, in some cases, although individual centrifugation may be costly, its combination with other technologies renders a lower cost (due to increased centrifugation efficiency and decreased input flow rate into other technologies).

#### Cell disruption technologies

**Fig. 8:**
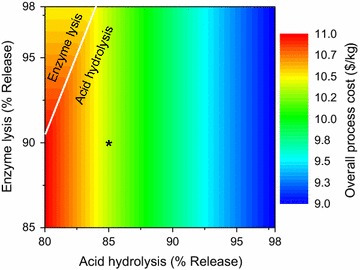
Overall process cost with variation in performance of cell disruption technologies. Direction of the contour lines denotes the viable region for the two technologies available for cell disruption: acid hydrolysis and enzyme lysis. They are *horizontal* where enzyme lysis is selected, whereas *vertical* where acid hydrolysis is selected. Critical values, when there is a change in technology selection from acid hydrolysis to enzyme lysis, are shown by *white lines*

The analysis for cell disruption technologies is performed by varying the percentage release of intracellular components for the three technology options: bead mill, acid hydrolysis, and enzyme lysis [122–124]. All the three available technologies can have variable component releases depending upon the type of microbial biomass handled, the entering feed characteristics, and product sensitivity to harsh conditions. The overall process cost as a function of the percentage release in acid hydrolysis and enzyme lysis is shown in Fig. 8. The base case is denoted by the black asterisk at coordinates (85, 90). In most cases, acid hydrolysis is the preferred option due to the low cost of acid in comparison with the high cost of enzymes.

Furthermore, we observe that the option of bead mill was not selected for this analysis as its cost (14.94$/kg at 98% release to 17.11$/kg at 80% release) was much higher when compared to other two disruption options.

#### Phase isolation technologies

**Fig. 9:**
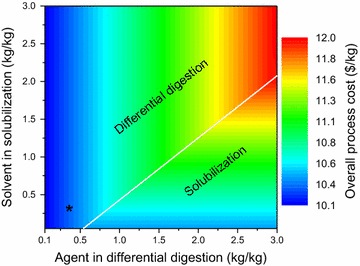
Overall process cost with variation in performance of phase isolation technologies. The *contour lines* are vertical in the region where differential digestion (Ddg) is selected, whereas they are *horizontal* in the region where solubilization (Slb) is selected. The critical values, when there is a change in technology selection from differential digestion to solubilization, are denoted by the *white lines*

The analysis for phase isolation technologies is performed by varying the amounts of agent required in differential digestion and solvent required in solubilization. The overall process cost as a function of the variation in amounts of digestion agent and solubilizing solvent is shown in Fig. 9. Solubilization selection adds additional cost in stage II because of the requirement of precipitation and microfiltration technologies. Thus, even if a digestion agent is required in higher amounts as compared to solvent, it is still preferable to select differential digestion rather than solubilization in most cases.

### Extension to other classes of IN NSL products

As per separation heuristics [84, 96], the most plentiful impurity for an intracellular product is the excess amount of water present in the form of extracellular liquid. Thus, cell harvesting is the foremost task for all classes of intracellular products. This task needs to be followed by cell disruption to release the product from the intracellular matrix. After disruption, the product needs segregation from other cell constituents. Generally, insoluble solids tend to separate out with the cell debris and hence require product segregation methods such as differential digestion and solubilization. Sometimes, if the solids are less dense than water (light) and have a relatively large particle size, they might easily float to the surface. Thus, low-cost technology, such as decantation, might suffice the product phase isolation task. Selective solubilization would be more favorable for lighter solids with smaller particle size.

Insoluble liquids that are lighter than water can be separated from solid impurities or heavier liquids using sedimentation, decantation, or centrifugation, which are usually low-cost technologies when less amount of materials are handled. Insoluble liquids if heavier than water might segregate along with the heavy solids and cell debris. Such products can be isolated using filtration or membranes depending upon the size of solid impurities. Thus, in the other three classes of intracellular insoluble products (IN NSL SLD LT CMD/SPC, IN NSL LQD LT CMD/SPC, and IN NSL LQD HV CMD/SPC), major differences occur in product isolation. The technologies are either similar to the ones discussed in the selected case study (solubilization or differential digestion) or simple low-cost options such as decantation, sedimentation, centrifugation, filtration, or membranes. Furthermore, the results for biomass titer suggest that the cost contribution of the phase isolation task is always less than 20% in the overall process cost. Thus, it is safe to say that the proposed framework and the results presented for the representative case study can help in deciding the optimal separation configurations for most intracellular insoluble products.

The current study does not consider parameters in stage II and stage III for further analysis as they do not contribute significantly in the separation of most intracellular, insoluble, high-volume chemicals. We did not include the scenario for high-value chemicals, because for these chemicals quality is a major concern and cost minimization becomes secondary [126]. However, for high-value chemicals a similar analysis can be performed to determine the impact of variation in technology parameters in the later separation stages II and III.

## Study 2: intracellular soluble products

We list the product classes for intracellular (IN) soluble (SOL) products (based on additional properties) and the separation options involved for each of them in Table 6. From the list of technologies for intracellular insoluble (Table 2) and intracellular soluble (Table 6) products, it is evident that the major differences are observed in stage II. Thus, we analyze the different options in stage II for understanding their effects in the overall separation process. For this study, we choose the class of intracellular (IN), soluble (SOL), liquid (LQD), volatile (VOL), and specialty (SPC) product.

**Table 6:** Product classes for intracellular and soluble type of product

Product classes	Product examples	Stage I technologies	Stage II technologies	Stage III technologies	References
IN SOL SLD (CMD/SPC)	Soluble proteins	Flc, Sdm, Ftt, Cnt, Ely, Bml, Hph, Mbr-MF/RO/UF, Slb	Mbr-all, Evp, Cnt, Prc, Ads	Mbr-all, Blc, Crys, Dry	[127, 128]
IN SOL LQD VOL (CMD/SPC)	Some algal biofuels	Flc, Sdm, Ftt, Cnt, Ely, Bml, Mbr-MF/RO/UF, Slb	Mbr- all, Ext, Dst, Atpe, Evp, Ads, Pvp	Mbr-all, Chr, Blc, Pvp	[129]
IN SOL LQD NVL (CMD/SPC)	Soluble hormones	Flc, Sdm, Ftt, Cnt, Ely, Bml, Mbr-MF/RO/UF, Slb	Mbr- all, Ext, Dst, Atpe, Evp, Ads, Pvp	Mbr-all, Chr, Blc, Pvp	[130]

*Mbr-all* is inclusive of all types of membrane technologies (MF, UF, RO, NF)

Full forms of the technology abbreviations used are listed earlier in Table 1

### Separation superstructure for IN SOL LQD VOL SPC product

**Fig. 10:**
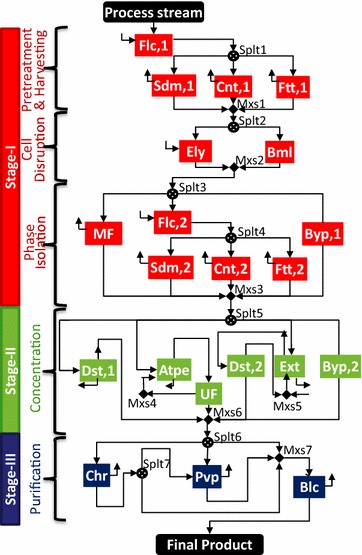
Separation superstructure for intracellular (IN) soluble (SOL) liquid (LQD) volatile (VOL) specialty (SPC) product. The three stages are distinguished based on *colors*: (I) Cell and product isolation: *red*; (II) Product concentration: *green*; and (III) Product purification and refinement: *blue*. The technologies involved are flocculation (Flc,1,2), sedimentation (Sdm,1,2), centrifugation (Cnt,1,2), filtration (Ftt,1,2), enzyme lysis (Ely), bead milling (Bml), membrane processes (microfiltration (MF) and ultrafiltration (UF)), distillation (Dst,1,2), aqueous two-phase extraction (Atpe), extraction (Ext), chromatography (Chr), pervaporation (Pvp), and bleaching (Blc). Options for bypassing (Byp,1,2) a stage or some tasks are also included

The superstructure (Fig. 10) for IN SOL LQD VOL SPC product is developed from the general separation scheme [96] for all intracellular soluble products (see Additional file 1). We have five tasks in stage I: pretreatment-1, cell harvesting, cell disruption, pretreatment-2, and phase isolation. Pretreatment #1 and #2 are optional while the other three tasks are essential for all IN SOL products. We do not consider acid hydrolysis for cell disruption because previous literature [122, 131] does not support its suitability for most soluble products.

Due to low biomass titers, the product is usually present in dilute concentrations in the stream leaving stage I. Thus, the product is first concentrated in stage II using methods like distillation, aqueous two-phase extraction (ATPE) that involves a polymer–salt system [132, 133], or liquid–liquid extraction that involves an extraction solvent [134]. Distillation can concentrate the product by utilizing thermal energy (heat), whereas ATPE and extraction use mass separating agents, which require further separation from the product as well as recycling of the added agents. Thus, ATPE is followed by a membrane technology of ultrafiltration to recover product from polymer phase, and extraction is followed by distillation to recover the product from the solvent. For further purification and refining of the product, technologies like chromatography, pervaporation, and bleaching are available in stage III. Some basic input parameters for the case study are presented in Table 7.

**Table 7:** Important input parameters for the base case for IN SOL LQD VOL SPC product

Parameter	Nominal value	Units
Initial cell titer	5	g/L (kg/m^3^)
Product content in cells	20	wt% of cell dry weight (CDW)
Desired production capacity	500	kg/h
Annual operation time	330	Days/yr
Final product purity	99	wt% purity

### Cost contribution analysis

The objective is the same as in study 1, to minimize the overall process cost. The details regarding input conditions, technology parameters, and MINLP problem formulation are discussed in Additional file 1.

#### Optimal configuration

**Fig. 11:**
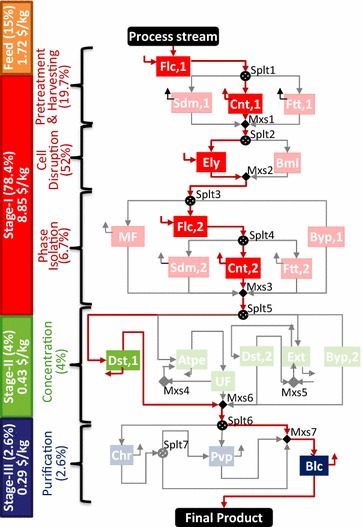
Technologies selected in the three separation stages for IN SOL LQD VOL SPC product. The active streams in the superstructure are shown by *bold red lines*. The selected technologies are* highlighted* in* different colors* corresponding to each stage: *red* for stage I, *green* for stage II, and *blue* for stage III. The stage-wise cost contribution analysis shows stage I to be the key cost driver, followed by feed, stage II, and stage III

The base case optimal configuration and the cost contributions are presented in Fig. 11. The technologies selected in stage I include flocculation (Flc,1) for pretreatment, centrifugation (Cnt,1) for cell harvesting, enzyme lysis (Ely) for cell disruption, flocculation (Flc,2) for pretreatment-2, and centrifugation (Cnt,2) for phase isolation. In stage II, distillation (Dst,1) is selected for product concentration. Pigments can sometimes impart undesirable color and appearance to the product. Hence, in stage III, bleaching (Blc) is used if the product is required to be colorless. Stage I is the key cost driver (~78.4%). The overall process cost is $11.29/kg product, wherein the separation cost contribution is $9.57/kg (~85%).

#### Alternate separation configurations

Similarly to case study 1, we use integer cuts successively to determine alternate separation configurations (see Table 8).

**Table 8:** Alternate separation configurations for study 2 using successive integer cuts

Configuration #	Technologies selected	Overall cost ($/kg)	Separation cost ($/kg)
Best case	Flc1, Cnt1, Ely, Flc2, Cnt2, Dst1, Blc	11.29 (NA)	9.57
2nd best	Flc1, Cnt1, Ely, Flc2, Sdm2, Dst1, Blc	11.38 (0.79%)	9.66
3rd best	Flc1, Cnt1, Ely, Flc2, Ftt2, Dst1, Blc	14.29 (26.57%)	12.14
4th best	Flc1, Sdm1, Ely, Flc2, Sdm2, Atpe, UF, Pvp, Blc	15.36 (36.04%)	13.09

#### Alternate concentration options in stage II

Distillation (Dst1) has been selected in the base case because the product is assumed to be volatile (relative volatility: 2.5) as compared to water. However, if the relative volatility is less than 1.05 then distillation is not preferred [135]. Thus, we also analyze the separation configurations when ATPE and extraction are selected in stage II.

**Fig. 12:**
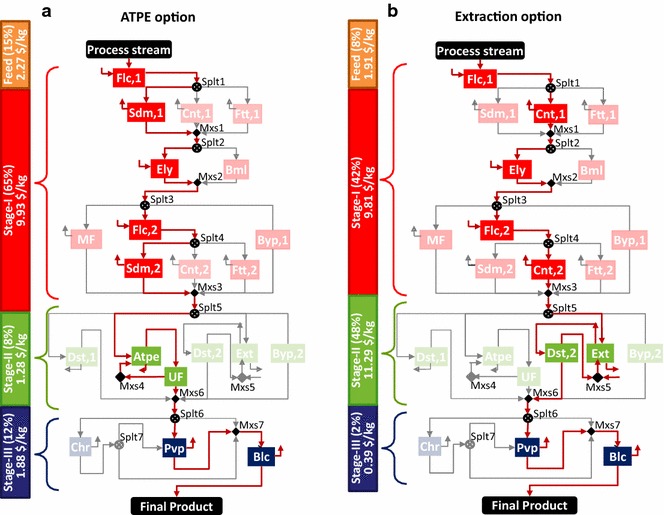
Technologies selected for ATPE and extraction options in stage II for IN SOL LQD VOL SPC product. **a** Technologies selected when Atpe and UF are chosen in stage II. **b** Technologies when Ext and Dst,2 are chosen in stage II. The active streams are shown by *bold red lines* and the selected technologies are highlighted in *different colors* corresponding to each stage: *red* for stage I, *green* for stage II, and *blue* for stage III. Stage-wise cost distribution assessment is also shown

*Option #2*: *Aqueous two-phase extraction* In this case, we choose the aqueous two-phase extraction (Atpe) option in stage II (refer Additional file 1). The technologies selected (Fig. 12a) in stage I include flocculation (Flc,1) for pretreatment, sedimentation (Sdm,1) for cell harvesting, enzyme lysis (Ely) for cell disruption, and flocculation (Flc,2) for second pretreatment, followed by sedimentation (Sdm,2) for phase isolation. In stage III, the remaining impurities are removed by pervaporation (Pvp) and bleaching (Blc) to achieve the desired product purity. The overall process cost is $15.36/kg product wherein the separation cost contribution is $13.09/kg (~85%). Stage I is still the major cost contributor, followed by feed, stage III, and stage II.

*Option #3*: *Extraction* In this case, we choose liquid–liquid extraction (Ext) as the concentration option in stage II and the following distillation (Dst,2) option for solvent recovery and recycling. The technologies selected (Fig. 12b) in stage I include flocculation (Flc,1) for pretreatment, centrifugation (Cnt,1) for cell harvesting, enzyme lysis (Ely) for cell disruption, and flocculation (Flc,2) and centrifugation (Cnt,2) for phase isolation. In stage III, the remaining impurities are removed by pervaporation (Pvp) and bleaching (Blc) to achieve the desired product purity. The overall process cost is $23.4/kg product wherein the separation cost contribution is $21.49/kg (~92%). Stage II becomes the key cost driver, followed by stage I, feed, and stage III.

### Important process considerations

Based on the base case configuration and other options in stage II, some key process parameters are identified. Details of the selected parameters and their range of variation are presented in Table 9.

**Table 9:** Parameters selected for analysis in study 2

Separation stage and task	Parameter	Base case (nominal)	Range	References
Stage I: input parameters	Biomass titer in input process stream	5 g/L	(0.1–200)	[59, 136]
	Product content in input process stream	20% CDW	(10–70)	[59, 137, 138]
Stage II: distillation #1(Dst,1)	Heat of vaporization of product	2000 kJ/kg	(700–2200)	[135, 139–141]
Stage II: ATPE (Atpe)	Partition coefficient of product in the top phase (KpT)	5 (−)	(5–10)	[133, 141–143]
Stage II: extraction (Ext)	Partition coefficient of product in solvent	1.2 (−)	(1.2–10)	[134, 141, 144]
	Solubility of solvent in water	0.03 kg/kg	(0.0002 –0.03)	
	Cost of solvent per unit mass	1.5 $/kg	(0.2–1.5)	

*Biomass titer and product content* We select the input parameters of biomass titer and product content [112, 115] for the first set of analysis. The biomass titer has been varied in the range of 0.1–250 g/L as it is the feasible range reported for intracellular soluble products for both photoautotrophic (0.1–10) [59] and heterotrophic (5–268) [136] routes of microbial cultivation. Also, product content in the biomass has been varied in the range of 10–70% CDW [59, 137, 138].

*Parameters in stage I* Important considerations for the tasks in stage I are similar to the ones discussed for case study #1, since the major distinguishing property is product localization. The cell harvesting options are the same as in case study #1, and hence we do not perform additional analysis. For cell disruption, we check the overall process cost when bead mill is selected and it is much higher (15.29$/kg at 98% release to 18.59$/kg at 80% release) as compared to enzyme lysis. Hence, bead mill is never selected when all the other parameters are at their nominal values (refer Table 7 and Additional file 1).

*Parameters in stage II* For soluble products, stage II can be crucial as it involves concentration of the product present in water-rich phase along with other soluble components. The properties like physical state and volatility play a major role in separating soluble products. Technologies using energy separating agent (ESA) like distillation can prove effective if the relative volatility of the product is high (>1.05—for the more volatile component) and the heat of vaporization is low. However, if these conditions are not satisfied, then technologies using mass separating agents (MSA) like ATPE (Atpe) and extraction (Ext) have to be used. The availability of a suitable MSA, ease of product purification, and MSA recycling are important considerations for the ATPE and extraction options in stage II.

We select parameters from these technologies and vary them in the range reported in Table 9 (refer Additional file 1). For distillation (Dst,1), the utility cost is the highest contributor, and hence we select the heat of vaporization of the product for further analysis. For ATPE (Atpe), the annualized capital cost is the highest contributor. The equipment size is a function of the feed and added materials during the separation process. We choose the partition coefficient for the top phase (KpT) as the varying parameter since it affects the amount of polymer (top phase) and salt (bottom phase) added in the ATPE unit. For extraction (Ext), the materials cost is the highest contributor, and hence we select the solvent-specific parameters of product partition coefficient (Kp) in solvent versus water, solubility of solvent in water, and the cost of solvent per unit mass for further analysis.

*Parameters in stage III* For specialty products, technologies in stage III have to be more stringent to meet the purity requirements. Based on the technologies used in the previous stages, the product-containing stream can have different components (other soluble materials and added separating agents such as solvents, polymers, and salts) and concentrations and hence one or more technology may be required to achieve the desired purity. The intracellular pigments impart undesirable color and appearance to the product; hence, bleaching is an important technology for specialty products. If chromatography is used for product purification, then the resulting stream will be dilute. Hence, pervaporation can be useful for the removal of excess water or solvents and further refinement of the product. However, the stage III parameters are not selected for further analysis as the cost contribution is less than 3% in the base case.

### Analysis and results for IN SOL LQD VOL SPC product

#### Biomass titer and product content

**Fig. 13:**
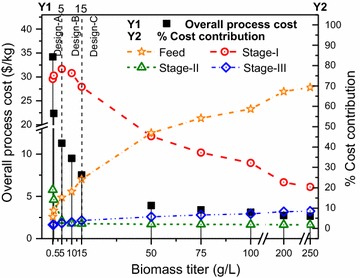
Overall process cost and contributions by feed and separation stages with varying biomass titer. Axis *Y*1 represents the overall process cost per unit product. Axis *Y*2 represents the cost contribution of the feed and three separation stages. The *vertical dotted lines* at 5 and 15 g/L represent the change in optimal separation network design

**Fig. 14:**
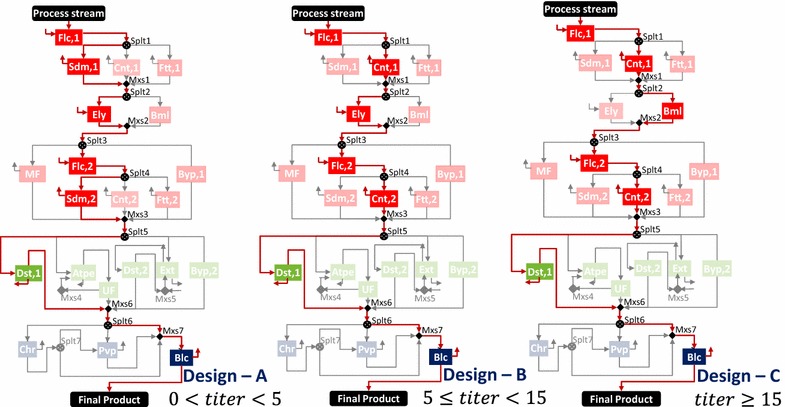
Selected technologies and active streams in designs* A*,* B*, and* C* for IN SOL LQD VOL SPC product. Design* A* is optimal for titers less than 5 g/L, design* B* is optimal for titers in the range of 5 (base case) to less than 15 g/L, and design* C* is optimal for titers 15 g/L and greater

*Biomass titer* The results of the analysis are shown in Fig. 13, wherein the overall process cost (axis *Y*1) as well as the cost contributions (axis *Y*2) of the feed and the three separation stages are presented. We observe that the optimal separation network design changes with the titer. When the titer is less than 5 g/L, design A is optimal; from 5 to less than 15 g/L, design B is optimal; and from 15 to 250 g/L, design C is optimal. The selected technologies for each design and active streams are highlighted in Fig. 14.

For lower biomass titers, Design A with sedimentation options in stage I prove to be optimal when compared to centrifugation options in the base case (Design B). This is because the low biomass titer in the input stream requires more materials to be handled in stage I, which makes centrifugation and filtration more expensive than sedimentation, even though it offers a lower biomass recovery. For titer values of 15 and greater, the bead mill (design C) is selected for cell disruption because of the trade-off between annualized capital cost (high for Bml and low for Ely) and material cost (none for Bml and high for Ely). As titer increases, the entering feed reduces in both bead mill and enzyme lysis. However, the enzyme required in enzyme lysis for a fixed production level remains constant even if the feed amount reduces. Thus, the materials handled will always be more in enzyme lysis when compared to bead mill.

**Fig. 15:**
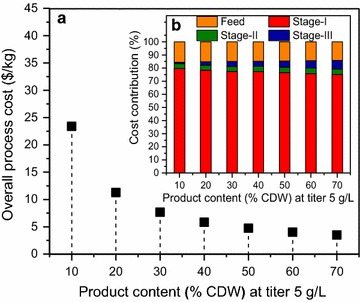
Overall process cost and contributions by feed and separation stages with variation in product content. **a** Overall process cost per unit product with varying product content (% CDW: cell dry weight) at constant biomass titer (5 g/L). **b** *Bar chart* showing cost contributions by feed (*yellow*) and the three separation stages (stage I: *red*, stage II: *green*, and stage III: *blue*). The technology selection is the same for all these values—design B reported for this case study

*Product content* The overall cost as a function of product content is shown in Fig. 15a. We observe a decrease in the overall process cost with the increase in product content. This happens because the higher the product content, the lower the amount of entering stream in the separation network for maintaining a constant production level. This decreases the feed cost as well as the cost incurred in each separation stage. The bar chart in plot (B) shows the contribution of feed and separation stages in the overall cost at varying product content, and this remains relatively constant for feed, stage I, and stage II and increases slightly with the increasing product content for stage III. This is because the technologies selected and the active process streams are the same. The lower amount of feed entering the separation network decreases the amount of materials handled in stages I and II; however, the materials handled in stage III remain fairly constant which increases its percentage contribution slightly with the increasing product content.

**Fig. 16:**
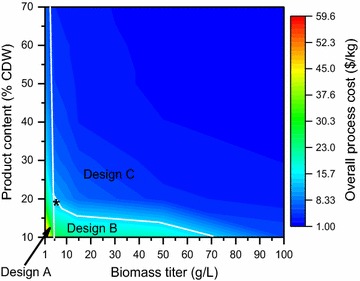
Overall process cost as a function of biomass titer and product content. The change in optimal separation network design is denoted by the *white lines* and the details of the selected technologies and active stream in designs *a*, *b*, and *c* are the same as those represented in Fig. 14. The base case (5 g/L, 20% CDW) is denoted by the *black asterisk*

*Simultaneous variation in biomass titer and product content* We vary biomass titer (1–100 g/L) and product content (10–70%CDW) as shown in Fig. 16. We observe that design A (refer Fig. 14) is optimal for very low biomass titer values irrespective of the product content. Design B is optimal for titer values up to 70 g/L but with low product content (≤20%CDW at 5 g/L). The optimal separation network changes to design C for most of the biomass titer and product content values.

#### Heat of vaporization of product

**Fig. 17:**
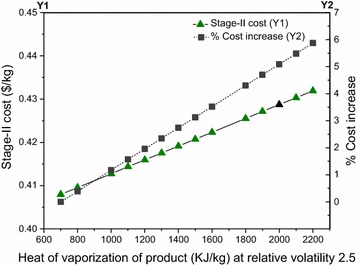
Stage II cost (*Y1*) and % increase (*Y2*) as a function of heat of vaporization of product. The base case is shown by the *black triangular* marker at (2000, 0.428)

The overall process cost increases with the increase in heat of vaporization (Additional file 1). This increase is not significant since the percentage contribution of distillation in the overall process cost is just ~4%. However, the stage II cost can increase by almost 6% if the heat of vaporization increases to 2200 kJ/kg from 700 kJ/kg (see Fig. 17). This happens because utilities are the major cost drivers in stage II; the more the heat of vaporization of the volatile component (product), the more heating utility will be required to perform distillation.

#### Partition coefficient for ATPE

**Fig. 18:**
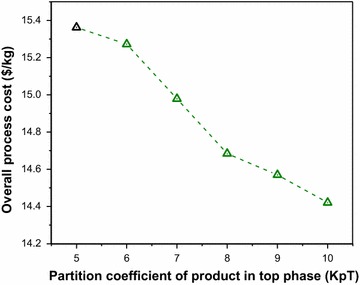
Overall process cost as a function of partition coefficient of product in the top phase (KpT). The result with nominal KpT value is shown by the *black* marker at (5, 15.36)

In Fig. 18, we observe that the overall process cost decreases with the increase in partition coefficient. The increasing partition coefficient enables more amount of product to be extracted in the top phase. Thus, less amount of separating agents, i.e., polymer and salt, are required for product concentration. Along with the decrease in overall process cost, we also observe a change in the optimal separation design at KpT values of 6 and 8, and thus instead of a smooth decrease we see the trend change at the aforementioned KpT values.

#### Extraction parameters

The change in partition coefficient (Kp) does not have a significant effect on the overall process cost (refer Additional file 1). The product recovery is a fixed parameter for the extraction technology; hence, at low Kp the increased equipment size and additional number of extraction stages contribute to the desired product recovery. As Kp increases, the equipment size and number of extraction stages decrease, hence resulting in slightly lower cost at higher Kp values. However, this change is not significant when compared to materials cost contributed by the added solvent.

**Fig. 19:**
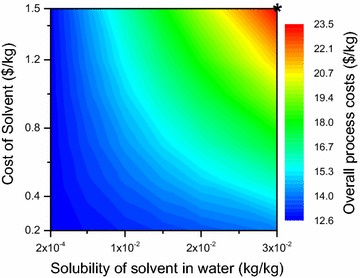
Overall process cost as a function of solubility of solvent and cost of solvent. The nominal values assumed for the three parameters (Kp—1.2 (not shown), cost of solvent—1.5 $/kg (*Y* *axis*), and solubility of solvent in water—0.03 kg/kg (*X* *axis*) and the corresponding overall process cost are shown by the *black asterisk*

The result for the solubility of solvent in water and the cost of solvent per unit mass is shown in Fig. 19. The nominal case of high solubility (0.03 kg solvent/kg water) and high solvent cost (1.5 $/kg) has an overall process cost of 23.5 $/kg. This can be reduced to 12.6 $/kg if the solubility decreases to 0.0002 kg/kg and the solvent cost decreases to 0.2 $/kg. Thus, finding a solvent with low water solubility and low cost even if the partition coefficient values are low is beneficial. This is because low solubility will enable higher recycling and less solvent will be required to replenish the solvent loss in raffinate phase.

### Extension to other classes of IN SOL products

For intracellular soluble products, additional properties such as physical state and volatility are important while separating the product from other components. In the case study, we considered a liquid product which is not temperature sensitive. Hence, the volatility gradient plays a major role in deciding the optimal product concentration technology. If the desired product is in solid state, then membranes, evaporation, precipitation, or adsorption can be used for product concentration. Membranes and precipitation costs (refer case study #1, intracellular insoluble chemicals) are lower when compared to evaporation (energy intensive) and adsorption (materials costs and regeneration of adsorbent).

The purification and refinement technologies may include crystallization and/or drying, based on market requirements. Drying cost can be in a similar range reported for case study #1 (~11% contribution); however, crystallization cost is dependent on the product’s solubility as a function of temperature (cooling or evaporative crystallization), heat of crystallization, and structure-specific properties such as chiral composition [145] and preferred crystal shape. Usually, evaporative crystallization is expensive as compared to cooling crystallization, since it involves two operations, evaporation followed by crystallization. Cooling crystallization requires a substantial variation in solubility with temperature and its cost contribution can range from ~9% [80] to 26% (Additional file 1) based on the properties of the product.

## Conclusion

We studied separation systems for the recovery of intracellular chemicals produced by microbial conversions and presented two representative case studies that include most technologies which are common to other intracellular products.

The first study (IN NSL SLD HV CMD) is a representative for intracellular, insoluble chemicals. In this study, we found that product separation contributed to almost ~84% in the overall process cost and stage I was the major cost driver with ~73% cost contribution. Further analysis suggests that the overall cost can decrease significantly with an increase in biomass titer up to 50 g/L, a point beyond which the overall cost remains fairly constant. Major shifts in technology selection occur for the tasks of cell disruption and phase isolation in stage I.

For cell harvesting, centrifugation is the preferred option in most cases, and if we can improve its efficiency, we may be able to reduce the cost by almost ~54%. For cell disruption, acid hydrolysis is the preferred technology for insoluble products, and by improving the release parameters the cost can be reduced by ~13%. This proves that the process is more sensitive to the technology selected for cell harvesting as compared to disruption and indicates that further research and development is required for performance enhancement of harvesting technologies. For product isolation, differential digestion of NPCM is more favorable than product solubilization. Thus, there is a need to find digestion agents which do not affect the product quality and are required in reasonable amounts.

The second study (IN SOL LQD VOL SPC) is a representative case for intracellular soluble chemicals. Separation again was the major cost contributor (~85%). We found that biomass titer affected the optimal separation network design and overall process cost at constant product content, whereas change in product content at low biomass titers (≤5 g/L) did not alter the optimal design but affected the overall process cost. Further analysis suggested that simultaneous improvements in both parameters can reduce the overall process cost significantly.

Stage II is crucial for soluble products and the cost contribution in the overall process can vary significantly depending upon the available technologies. We observed that stage II could become dominant if extraction is the only feasible option. The analysis for alternate options in stage II revealed that distillation (option #1: base case) cost can increase slightly with the increase in heat of vaporization of the product (more volatile or light key component). For ATPE (option #2), it was observed that with the increase in product partition coefficient the overall cost could be decreased. For extraction (option #3), it was found that the process cost reduced significantly when a solvent was almost insoluble in water and was available at low cost, but the change in partition coefficient had no significant effect.

In the current studies, we include most common technologies to generate reliable process insights (e.g., identifying biomass titer, product content, separation efficiency, etc. as the key cost influencers). However, new technologies and products can be taken into account by changing model parameters and/or adding case-specific constraints in the model. Thus, we believe that the proposed analysis will prove valuable in selecting new target bio-based chemicals that can be produced economically using microbial bioconversions and for designing cost-effective separation processes.

Furthermore, this study addresses the change in ‘optimal’ system performance. The predictions associated with the changing inputs and model parameters can assist in the development of policies or general guidelines for similar systems. Additionally, the model can be extended to account for environmental constraints with regard to waste streams or the overall environmental footprint. This can be achieved by (1) adding constraints representing environmental regulations [147–150] and/or (2) modifying the objective function to include an environmental sustainability indicator [146] and employing a multi-objective optimization approach. Most importantly, it helps in identifying some promising research directions in the area of separations, a topic that has not received its due attention despite its substantial impact on the economics of biomass-to-fuels/chemicals strategies.

## Product properties

(In the order of classification)–NSL: insoluble; SOL: soluble; SLD: solid; LQD: liquid; HV: heavy; LT: light; VOL: volatile (more volatile that water); NVL: non-volatile (less volatile than water); CMD: commodity; SPC: specialty.

## Technologies

Ads: Adsorption; Ahy: acid hydrolysis; Atpe: aqueous two-phase extraction; Blc: bleaching; Bml: bead mill; Chr: chromatography; Chy: chemical lysis; Cnt: centrifugation; Cog: coagulation; Crs: crystallization; Ddg: differential digestion; Dry: drying; Dst: distillation; Ely: enzyme lysis; Evp: evaporation; Ext: extraction; Flc: flocculation; Flt: flotation; Ftt: filtration; Hph: homogenization; MF: microfiltration; Mxs: mixer; NF: nanofiltration; Prc: precipitation; Pvp: pervaporation; RO: reverse osmosis; Sdm: sedimentation; Slb: solubilization; Splt: splitter; UF: ultrafiltration.

## Others

CDW: cell dry weight; Kp: product partition coefficient in solvent phase for Ext technology; KpT: product partition coefficient in the top phase for Atpe technology; MINLP: mixed-integer non-linear programming; NPCM: non-product cellular materials.

## Additional file

**Additional file 1.** **A**. Information about intracellular chemicals: includes previous studies and economic assessment for some intracellular chemicals. **B**. Case study 1 – Intracellular insoluble product: includes **B.1.** Separation scheme and superstructure for intracellular insoluble product, **B.2.** Model details and equations, **B.3** Model parameters and input data, **B.4.** Integer-cuts for alternate configurations and **B.5.** Some additional results. **C**. Case study 2 – Intracellular soluble product: includes **C.1.** Separation scheme and superstructure for intracellular soluble product, **C.2.** Model details and equations, **C.3.** Model parameters and input data, **C.4.** Integer-cuts for alternate configurations and **C.5.** Some additional results.
