# Supplementary material for: Synthesis and analysis of separation networks for the recovery of intracellular chemicals generated from microbial-based conversions
Source: Biotechnol Biofuels. 2017 May 8;10:119. doi: 10.1186/s13068-017-0804-2 (PMC5422901; doi:10.1186/s13068-017-0804-2)
Supplement: Supplementary file 1 — Additional file 1. A. Information about intracellular chemicals: includes previous studies and economic assessment for some intracellular chemicals. B. Case study 1 – Intracellular insoluble product: includes B.1. Separation scheme and superstructure for intracellular insoluble product, B.2. Model details and equations, B.3 Model parameters and input data, B.4. Integer-cuts for alternate configurations and B.5. Some additional results. C. Case study 2 – Intracellular soluble product: includes C.1. Separation scheme and superstructure for intracellular soluble product, C.2. Model details and equations, C.3. Model parameters and input data, C.4. Integer-cuts for alternate configurations and C.5. Some additional results. [file 13068_2017_804_MOESM1_ESM.pdf]

Synthesis and analysis of separation networks for the recovery of intracellular chemicals generated from microbial-based conversions

Kirti M. Yenkie, Wenzhao Wu, Christos T. Maravelias*

Department of Chemical and Biological Engineering, University of Wisconsin, Madison, WI – 53706.

DOE Great Lakes Bioenergy Research Center, University of Wisconsin-Madison, 1552 University Ave, Madison, WI 53726, USA

ADDITIONAL FILE

A. Information about intracellular chemicals

Table A.1 presents information from previous studies regarding the important process parameters and assumptions for production and recovery of some intracellular chemicals.

Table A.1 Previous studies on economic assessment for production and recovery of intracellular chemicals

Product example	Important assumptions and parameters	Results and important findings	Limitations	References
Poly-hydroxybutyrate (PHB)	Two process flowsheets for PHB isolation: (i)surfactant digestion (ii)solubilization in $CHCl_3$<br>Capacity: 2850 tons/year<br>Depending on microbial species; biomass titer: 15-110 g/L and product content: 40 to 70 %CDW	Production cost ranges from 5.58 to 11.96 $/kg PHB	No optimization only process simulation<br>No direct comparison of alternate technologies	[1, 2]
Cyanophycin	Substrate: food industry waste stream<br>Capacity: 1200 tons/year<br>Biomass titer: 5g/L<br>Product content: 25% (CDW)	Production cost estimated as 5.61 $/kg cyanophycin	Analysis based on comparison with other chemicals<br>No details of process flowsheet or technologies	[3, 4]
β-phycoerythrin (BPE)	Major technologies in flowsheet: photobioreactor, bead mill, precipitation, aqueous two phase extraction, ultrafiltration<br>Capacity: 149 mg/batch<br>Biomass titer: ~ 6g/L<br>Product content: 1% (CDW)	Production cost estimated as $1.17 $/mg of BPE	Process simulation on pilot scale<br>No sensitivity information	[5]
Astaxanthin	Major technologies in flowsheet: Filtration, centrifuge, bead mill, acid hydrolysis, precipitation, drying<br>Capacity – Pilot scale	Production cost estimated about 1600 – 7900 $/kg	Information collected from multiple studies<br>Usually lab and pilot scale simulations	[6–8]

B. Case study 1 – Intracellular insoluble product

B.1 Separation scheme and superstructure for intracellular insoluble product

The general separation scheme for all intracellular (IN) and insoluble (NSL) products is presented in Figure B.1 and is adapted from previous work on separation schemes for bio-based chemicals [9].

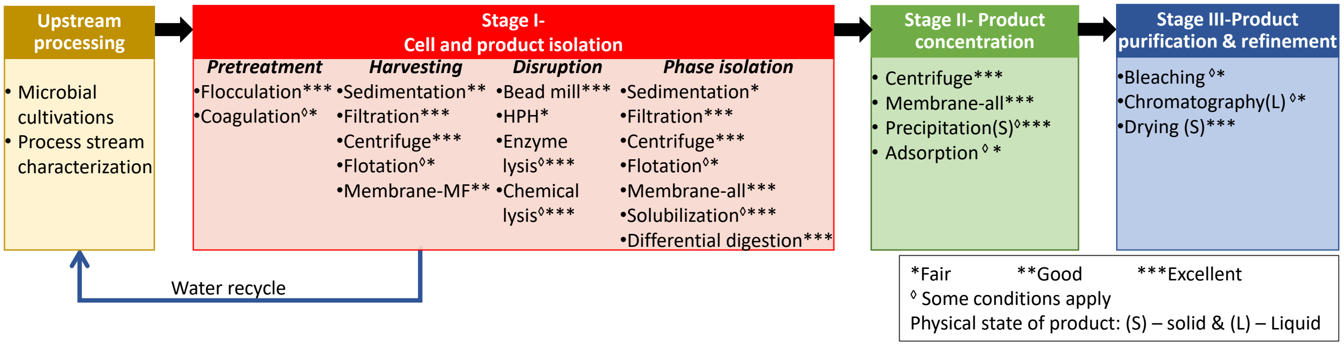

Figure B.1 Separation scheme for intracellular (IN) and insoluble (NSL) product. The color scheme denotes the three stages: stage I – red, stage II –green and stage III – blue. The technology suitability has been divided into grades - fair (*), good (**) and excellent (***), if applicable under certain conditions (◊) and physical state of the product (solid (S) and liquid (L)).

We reduce the list of technologies based on additional product properties such as solid (SLD), heavy (HV), and commodity (CMD) grade, considered for case study I. The simplified scheme for the additional property descriptors is shown in Figure B.2. This enables the generation of a more relevant separation superstructure [10–12], which can be used for the systematic synthesis of separation processes. The separation superstructure for the class IN-NSL-SLD-HV-CMD is shown in Figure B.3.

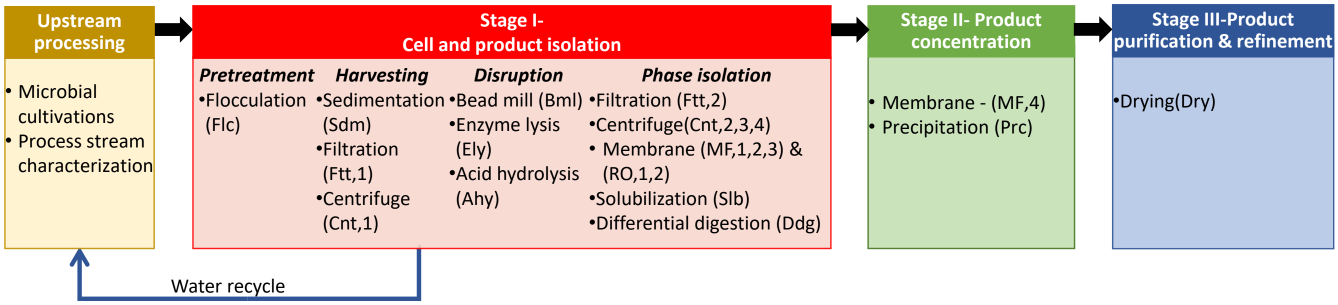

Figure B.2 Separation scheme for product class intracellular-insoluble-solid-heavy-commodity (IN-NSL-SLD-HV-CMD) chemical. The reduced list of relevant technology options in each stage are listed in the corresponding boxes.

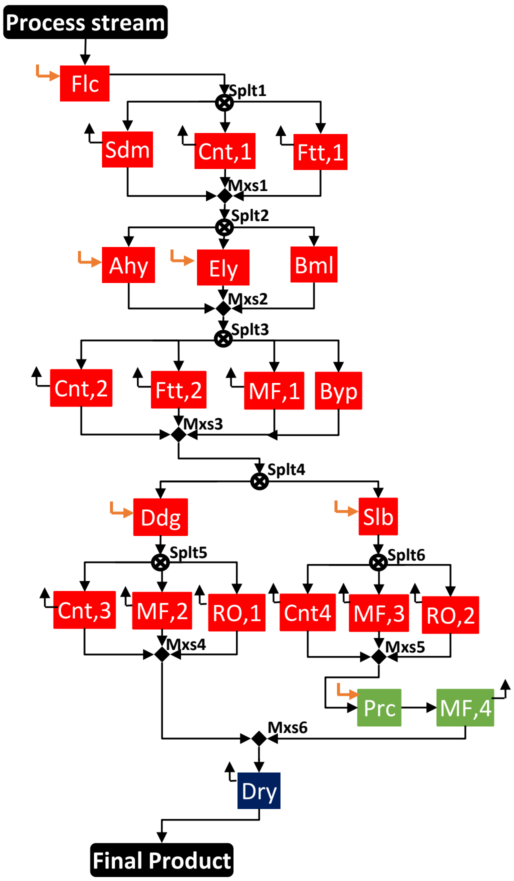

Figure B.3 Separation superstructure for intracellular (IN), insoluble (NSL), solid (SLD), heavy (HV), commodity (CMD) product. It consists of three stages distinguished using different colors: (I) Cell and product isolation - red (II) Product concentration – green (III) Product purification and refinement – blue. The technologies involved are flocculation (Flc), sedimentation (Sdm), centrifugation (Cnt,1,2,3,4), filtration (Ftt,1,2), acid hydrolysis (Ahy), enzyme lysis (Ely), bead milling (Bml), membrane processes (microfiltration (MF,1,2,3,4) and reverse osmosis (RO,1,2)), differential digestion (Ddg), solubilization (Slb), precipitation (Prc) and drying (Dry). An option for bypassing (Byp) a set of parallel technologies for phase isolation is included in stage-I.

B.2 Model details and equations

Notations

- *The 'uppercase italic Latin fonts (not colored)' are for variables (optimization variables)*
- *The uppercase Latin fonts and lowercase Greek fonts in red are the specified input parameters*
- *The uppercase Latin fonts and lowercase Greek fonts in green color are for the parameters evaluated from inputs available*
- *The parameter or variable to be evaluated is always on the L.H.S. of the equation*

B.2.1 Indices and sets

$i \in \boldsymbol{I}$ - technologies (used as subscript to variables)

{*flc* - flocculation,

sdm - sedimentation,

splt# and *mxs#* - splitters and mixers,

cnt# - centrifugation and # = {1, 2, 3, 4},

ftt# - filtration and # = {1, 2},

mbr - membranes, MF (*mf#*) – microfiltration {1, 2, 3, 4} and RO (*ro#*) – reverse osmosis {1, 2}

ahy - acid hydrolysis (cell disruption by chemical method)

ely - enzyme lysis (cell disruption by biological method)

bml - bead mill (cell disruption by mechanical method)

byp - bypass

ddg - differential digestion of NPCM (non-product cell materials)

slb - solubilization of product

prc - precipitation

dry - drying}

$j \in \boldsymbol{J}$ - stream (used as subscript to variables)

{1, 2, 3, 4, ………………….., 68}

$k \in \boldsymbol{K}$ - components (used as subscript to variables)

{B – biomass, *W* – water, *Prd* – product, *Prot* – proteins, *Lpd* – lipids, *RNA*, *Oth* – others, *Deb* – debris, *RS* – residual solids, *Flcnt* – flocculent added*, Acid* – acid added, *Enz* – enzyme added*, Solv* – solvent, *Agnt* – digestion agent, *Ansl* – antisolvent in precipitation}

$s \in \boldsymbol{NS}$ – stages {*s1, s2, s3*}

$l \in \boldsymbol{L}$ - utilities {electricity (*elec*), steam (*stm*), cooling water (*cwt*), refrigerant (*rfrg*)}

B.2.2 Subsets

Subsets for technologies

$\boldsymbol{I}^{\mathbf{CST}}$ – technologies with costs

{*flc, sdm, cnt1, ftt1, ahy, ely, bml, cnt2, ftt2, mf1, ddg, slb, cnt3, mf2, ro1, cnt4, mf3, ro2, prc, mf4, dry*}

$\boldsymbol{I}^{\mathbf{CF}}$ – technologies with concentration factor

{*sdm, cnt1*, *cnt2, cnt3, cnt4, ftt1, ftt2, mf1, mf2, mf3, mf4, ro1, ro2*}

$\boldsymbol{I}^{\mathbf{CONS}}$ – technologies having consumables

{*ftt1, ftt2, mf1, mf2, mf3, mf4, ro1, ro2*}

$\boldsymbol{I}^{BV}$ – technologies having binary selection constraints

{*sdm, cnt1, ftt1, ahy, ely, bml, cnt2, ftt2, mf1,byp, ddg, slb, cnt3, mf2, ro1, cnt4, mf3, ro2, prc, mf4*}

$\boldsymbol{I}^{S1}$ – technologies in stage I

{*flc, sdm, cnt1, ftt1, ahy, ely, bml, cnt2, ftt2, mf1,byp, ddg, slb, cnt3, mf2, ro1, cnt4, mf3, ro2*}

$\boldsymbol{I}^{S2}$ – technologies in stage II

{*prc, mf4*}

I^{S3} – technologies in stage III

{*dry*}

Subsets for streams

J^{BD} – streams before disruption

{1 to 14}

J^{AD} – streams after disruption

{23 to 68}

Subsets for components

K^{JP} – components in process streams

{*B, W, Acid, Enz, Prd, Prot, Carb, RNA, Lpd, Deb, Oth, RS, Solv, Agnt, AnSl*}

K^{BD} – basic components before disruption

{*B, W*}

K^{C} – components in cell or biomass

{*Prd, Prot, Carb, RNA, Lpd, Deb, Oth*}

K^{H1} – heavy components in stage-1

{*Prd, Prot, RNA, Deb, RS*}

K^{L1} – light components in stage-1

{*Carb, W, Lpd, Oth, Acid, Enz*}

K^{DH1} – heavy components in stage-1 after ddg

{*Prd*}

K^{DL1} – light components in stage-1 after ddg

{*Prot, RNA, Deb, RS, Carb, W, Lpd, Oth, Acid, Enz, Agnt*}

K^{SH1} – heavy components in stage-1 after slb

{*Prot, RNA, Deb, RS, Carb, Lpd, Oth, Acid, Enz*}

K^{SL1} – light components in stage-1 after slb

{*Prd, Solv, W*}

K^{ADD} – externally added components

{*Flcnt, Acid, Enz, Solv, Agnt, AnSl*}

K^{SOL} – externally added solvent components

{*Acid, Enz, Solv, Agnt, AnSl*}

K^{NS} – non-solvent components

{*B, W, Prd, Prot, Carb, RNA, Lpd, Deb, Oth, RS*}

K^{OTP} – components other than product

{*B, W, Acid, Enz, Prot, Carb, RNA, Lpd, Deb, Oth, RS, Solv, Agnt, AnSl*}

B.2.3 Dynamic sets for connectivity

To denote connection between technologies, streams and components (*Yes* if connected, *No* if not)

$\boldsymbol{J_i}$ – streams of technology *i*

$\boldsymbol{Jin_i}$ – inlet streams of technology *i*

$\boldsymbol{Jout_i}$ – outlet streams of technology *i*

$\boldsymbol{K_i}$ – components *k* in technology *i*

$\boldsymbol{K_j}$ – components *k* in stream *j*

B.2.4 Model Parameters

General parameters

Υ_i (%) = Release parameter in disruption technologies

ζ_i (m^3/m^2h) = Flux of technology i {*ftt* and *mbr* technologies}

η_i(%) = Efficiency of technology i

$\theta_i(h)$ = Residence time in technology i

θ_i^{Rep}(h/year) – Replacement time for consumables in technology i

κc_{dry} (%) = Sublimation percentage in dryer

λ_{stm}(KJ/kg) = Latent heat of steam

$\lambda^{vap}{}_k$(KJ/kg) = Heat of vaporization of component k

$\xi_{k,i}$(——) = Retention factor of component k for technology i {ftt and mbr technologies}

π_{feed}($/kg biomass) = Entering feed cost in terms of per kg biomass

$\pi^{Rep}{}_i$ ($/unit) = Replacement cost of consumables per unit capacity in technology i

$\pi_{k/l}$ ($/unit) = Unit price of component k or utility l

ρ_k(kg/m^3) = Density of component k

ς_{RF}(——) = Capital recovery/charge factor (0.11)

τ_{ann}(h/annum) = Annual operating time in hours (330 day/annum x 24 h/day = 7920 h/annum)

$\phi_{add,k}$ (kg/kg) = Addition amounts for flocculants and enzyme

$\psi_{k-k'}$(kg/kg) = Solubility of component k in k'

BMC_{mult}(——) = Bare module cost multiplier (5.4)

C_{bio}(kg/m^{3}or g/L) = Biomass titer

C_{Lab}($/h) = Labor cost - operator basis (30)

Cp_k(KJ/kg-°C) = Specific heat of component k

$C0_i$($/capacity) = Cost of a technology with standard capacity

d_p(m) = Particle diameter

d_{floc}(m) = Floc diameter

Frc_k (——)= Fraction of cellular component k in biomass

g(m/s^2) = Acceleration due to gravity

$M1_k(--)$ = Big-M constant for component k

MW_k(kg/mol) = Molecular weight of component k

$nc_i(--)$ = cost scaling index for technologies

$Nlabr_i(\#/h)$ = # of laborers required for technology *i* per hour

Prd_F(kg/h) = Final product recovered per hour (1000)

Pur (%) = purity of product (95)

$Q0_i$(m^3 or m^2 or m^3/h) = Standard capacity of a technology for costing, labor and power required (varies – details in **Table A.3.18** in section A.3)

T_{amb} (°C) = Ambient temperature (25)

T_{sat}(°C) = Saturation temperature (100)

Tcw_{in}(°C) = Cooling water temperature in (25)

Tcw_{out} (°C) = Cooling water temperature out (30)

T_{frz} (°C) = Freezing temperature in freeze dryer (0.00001)

$T\text{rfrg}_{in}$(°C) = Refrigerant temperature in (-10)

$T\text{rfrg}_{out}$ (°C) = Refrigerant temperature out (0.00001)

$UA_i(KJ/m^2$°C) = Heat transfer coefficient

$Wsp_i(kW/h)$ = Power required by technology *i* per hour

Evaluated parameters

ω_i(rpm) = angular velocity in centrifuge technologies *i* = {cnt1, cnt2, cnt3, cnt4}

SOR_i(m/s) = surface overflow rate in sedimentation, *i* = {sdm}

$U_{g,Floc}$(m/s) = settling velocity of flocs

$U_{g,i}$(m/s) = settling velocity in centrifuge technologies *i* = {cnt2, cnt3, cnt4}

B.2.5 Model Variables

General variables

Ce_i($/capacity) = Purchase cost for technologies $i \in \boldsymbol{I^{CST}}$

CF_i(m^3/m^3) = Concentration factor for technologies $i \in \boldsymbol{I^{CF}}$

C_{Feed}($/h) = Purchase cost of feed stream

$Cons_i$($/annum) = Consumable costs for technologies $i \in \boldsymbol{I^{CONS}}$

$Cpur_k$($/h) = Purchase cost of added components ($k \in \boldsymbol{K^{ADD}}$)

E_{sub}(KJ/h) = Energy required for sublimation

$M_{j,k}$(kg/h) = Mass flowrate of component k in stream j

Mcw_i(kg/h) = Amount of cooling water required for technologies $i \in \boldsymbol{I^{CST}}$

$Mstm_i$(kg/h) = Amount of steam required for technologies $i \in \boldsymbol{I^{CST}}$

Nlb_i(#/h) = Number of laborers required for technologies $i \in \boldsymbol{I^{CST}}$

PW_i(KW/h) = Power required for technologies $i \in \boldsymbol{I^{CST}}$

Qc_i (m^3 or m^2 or m^3/h) = Costing variable for technologies $i \in \boldsymbol{I^{CST}}$

QW (m^3/h) = Entering stream flowrate

Stage-wise costing variables:

$CCAC_{Nstg}$= Fixed (annualized capital) cost in nth stage

$CCCS_{Nstg}$= Consumables cost in nth stage

$CCLB_{Nstg}$= Labor cost in nth stage

$CCOT_{Nstg}$= Other cost in nth stage (overheads and supervision costs)

$CCRM_{Nstg}$= Materials cost in nth stage

$CCTC_{Nstg}$= Total cost in nth stage (all costs added)

$CCUT_{Nstg}$= Utility cost in nth stage

CCTAC, CCTCS, CCTFC, CCTLB, CCTOT, CCTPC, CCTRM, CCTUT = Total costs for the different categories for the complete separation process

Binary variables

y_i(——) = Binary variables for technologies to be selected $i \in \boldsymbol{I^{BV}}$

B.2.6 Model Equations:

Initial flow assignment equations:

$$M_{1,W} = \rho_W QW \qquad (1.1a)$$

$$M_{1,B} = C_{bio} QW \qquad (1.1b)$$

Component balances in all technologies:

$$\sum_{j \in \boldsymbol{Jin_i}} M_{j,k} = \sum_{j \in \boldsymbol{Jout_i}} M_{j,k}\ ;\ \forall\ k \in \boldsymbol{K^{JP}}\ and\ i \in \boldsymbol{I} \qquad (1.2)$$

Cost of entering feed:

$$C_{Feed} = \pi_{feed} M_{1,B} \qquad (1.3)$$

Cost of technologies:

$$\left(\frac{Ce_i}{C0_i}\right) = \left(\frac{Qc_i}{Q0_i}\right)^{nc_i}\ ;\ \forall i \in \boldsymbol{I^{CST}} \qquad (1.4)$$

Labor requirement in technologies:

$$Nlb_i Q0_i = Nlabr_i Qc_i\ ;\ \forall i \in \boldsymbol{I^{CST}} \qquad (1.5)$$

Consumable costs in technologies:

$$Cons_i = \frac{\tau_{ann}}{\theta_i^{Rep}} \pi^{Rep}{}_i Qc_i\ ;\ \forall i \in \boldsymbol{I^{CST}} \qquad (1.6)$$

Logical equations:

$$M_{j,k} - M1_k y_i \leq 0\ ;\ \forall\ i \in \boldsymbol{I^{BV}}, j \in \boldsymbol{J}, k \in\ \boldsymbol{K_i} and\ \boldsymbol{K_j} \qquad (1.7)$$

Technology selection equations:

Stage-I technologies:

Harvesting technologies:

$$y_{sdm} + y_{cnt1} + y_{ftt1} = 1 \quad (1.8)$$

Cell disruption technologies:

$$y_{ahy} + y_{ely} + y_{bml} = 1 \quad (1.9)$$

Phase isolation technologies:

$$y_{cnt2} + y_{ftt2} + y_{mf1} + y_{byp} = 1 \quad (1.10)$$

$$y_{slb} + y_{ddg} = 1 \quad (1.11)$$

$$y_{cnt3} + y_{mf2} + y_{rvs1} = y_{ddg} \quad (1.12)$$

$$y_{cnt4} + y_{mf3} + y_{rvs2} = y_{slb} \quad (1.13)$$

Stage-II technologies:

$$y_{prc} = y_{slb} \quad (1.14)$$

$$y_{mf4} = y_{prc} \quad (1.15)$$

Stage-I models: Flocculation, cell harvesting, cell disruption and phase isolation technologies

Flocculation (Flc):

Flocculent added:

$$M_{2,Flcnt} = \phi_{add,Flcnt}\left[\sum_{k\in \boldsymbol{K^{BD}}}\left(\frac{M_{1,k}}{\rho_k}\right)\right] \quad (2.1)$$

Flocculent cost:

$$Cpur_{Flcnt} = \pi_{Flcnt} M_{2,Flcnt} \quad (2.2)$$

Volume of flc1:

$$Qc_{flc} = \theta_{flc}\left[\sum_{k\in \boldsymbol{K^{BD}}}\left(\frac{M_{1,k}}{\rho_k}\right)\right] \quad (2.3)$$

Power required:

$$PW_{flc} = Wsp_{flc}\left[\sum_{k\in \boldsymbol{K^{BD}}}\left(\frac{M_{1,k}}{\rho_k}\right)\right] \quad (2.4)$$

Cell harvesting:

Sedimentation (Sdm):

Efficiency equations:

$$\eta_{sdm} = \frac{M_{8,B}}{M_{4,B}} \quad (3.1)$$

Written as: $\eta_{sdm} M_{4,B} = M_{8,B}$

$$\eta_{sdm} = \frac{M_{7,W}}{M_{4,W}} \tag{3.2}$$

Concentration factor (CF_{sdm}): (volume concentration factor)

$$CF_{sdm} = \frac{\left[\sum_{k\in \boldsymbol{Kj}}\left(\frac{M_{4,k}}{\rho_k}\right)\right]}{\left[\sum_{k\in \boldsymbol{Kj}}\left(\frac{M_{8,k}}{\rho_k}\right)\right]} \tag{3.3}$$

Written as: $CF_{sdm}\left[\sum_{k\in \boldsymbol{Kj}}\left(\frac{M_{8,k}}{\rho_k}\right)\right] = \left[\sum_{k\in \boldsymbol{Kj}}\left(\frac{M_{4,k}}{\rho_k}\right)\right]$

$2 \le CF_{sdm} \le 15$

Written as: $CF_{sdm} \le 15y_{sdm}$ and $CF_{sdm} \ge 2y_{sdm}$

Area of sedimentation tank:

$$Qc_{sdm} = \frac{\left[\sum_{k\in \boldsymbol{Kj}}\left(\frac{M_{4,k}}{\rho_k}\right)\right]}{SOR_{sdm}} \tag{3.4}$$

Centrifugation (Cnt,1):

Efficiency equations:

$$\eta_{cnt1} = \frac{M_{10,B}}{M_{5,B}} \tag{4.1}$$

$$\eta_{cnt1} = \frac{M_{9,W}}{M_{5,W}} \tag{4.2}$$

Concentration factor (CF_{cnt1}):

$$CF_{cnt1} = \frac{\left[\sum_{k\in \boldsymbol{Kj}}\left(\frac{M_{5,k}}{\rho_k}\right)\right]}{\left[\sum_{k\in \boldsymbol{Kj}}\left(\frac{M_{10,k}}{\rho_k}\right)\right]} \tag{4.3}$$

$2 \le CF_{cnt1} \le 20$

Sigma factor equation:

$$Qc_{cnt1}U_{g,Floc} = \left[\sum_{k\in \boldsymbol{Kj}}\left(\frac{M_{5,k}}{\rho_k}\right)\right] \tag{4.4}$$

Power required:

$$PW_{cnt1} = Wsp_{cnt1}\left[\sum_{k\in \boldsymbol{Kj}}\left(\frac{M_{5,k}}{\rho_k}\right)\right] \tag{4.5}$$

Power dissipation to heat is about 40%, hence cooling utility is required:

$$Mcw_{cnt1}Cp_W(Tcw_{out} - Tcw_{in}) = (0.4PW_{cnt1}) \tag{4.6}$$

Filtration (Ftt,1):

Retention factor equations:

$$\xi_{k,ftt1} = \frac{M_{12,k}}{M_{6,k}}\ ;\ \forall k \in \boldsymbol{K^{BD}} \tag{5.1}$$

Written as: $\xi_{k,ftt1}M_{6,k} = M_{12,k}$

Concentration factor (CF_{ftt1}):

$$CF_{ftt1} = \frac{\left[\sum_{k\in \boldsymbol{Kj}}\left(\frac{M_{6,k}}{\rho_k}\right)\right]}{\left[\sum_{k\in \boldsymbol{Kj}}\left(\frac{M_{12,k}}{\rho_k}\right)\right]} \tag{5.2}$$

$$2 \le CF_{ftt1} \le 30$$

Flux balance:

$$\zeta_{ftt1} Qc_{ftt1} = \left[\sum_{k\in \boldsymbol{Kj}}\left(\frac{M_{6,k}}{\rho_k}\right)\right]\left(1 - \frac{1}{CF_{ftt1}}\right) \tag{5.3}$$

Written as: $\zeta_{ftt1} Qc_{ftt1} CF_{ftt1} = \left[\sum_{k\in \boldsymbol{Kj}}\left(\frac{M_{6,k}}{\rho_k}\right)\right](CF_{ftt1} - 1)$

Power required:

$$PW_{ftt1} = Wsp_{ftt1} Qc_{ftt1} \tag{5.4}$$

Cell disruption technologies:

<u>Acid hydrolysis (Ahy):</u>

Component release equation:

$$M_{19,k} = Y_{ahy} Frc_k M_{15,B};\ \forall k \in \boldsymbol{K^C} \tag{6.1}$$

Residual component (RS) after release:

$$M_{19,RS} = M_{15,B} - \sum_{k\in \boldsymbol{K^C}} M_{19,k} \tag{6.2}$$

Acid added:

$$M_{18,Acid} = \phi_{add,Acid} \sum_{k\in \boldsymbol{K^{BD}}} M_{15,k} \tag{6.3}$$

Acid cost:

$$Cpur_{Acid} = \pi_{Acid} M_{18,Acid} \tag{6.4}$$

Costing variables:

$$Qc_{ahy} = \left[\sum_{k\in \boldsymbol{Kj}}\left(\frac{M_{19,k}}{\rho_k}\right)\right] \tag{6.5}$$

Power required:

$$PW_{ahy} = Wsp_{ahy} Qc_{ahy} \tag{6.6}$$

Steam required for heating to hydrolysis temperature:

$$Mstm_{ahy}\lambda_{stm} = \left[\sum_{k\in \boldsymbol{K^{BD}}} M_{15,k}\, Cp_k + M_{18,Acid} Cp_{Acid}\right](T_{ahy} - T_{amb}) \tag{6.7}$$

<u>Enzyme lysis (Ely):</u>

Component release equation:

$$M_{21,k} = Y_{ely} Frc_k M_{16,B};\ \forall k \in \boldsymbol{K^C} \tag{7.1}$$

Residual component (RS) after release:

$$M_{21,RS} = M_{16,B} - \sum_{k\in \boldsymbol{K^C}} M_{21,k} \tag{7.2}$$

Enzyme added:

$$M_{20,Enz} = \phi_{add,Enz} M_{16,B} \tag{7.3}$$

Enzyme cost:

$$Cpur_{Enz} = \pi_{Enz} M_{20,Enz} \tag{7.4}$$

Costing variables:

$$Qc_{ely} = \left[\sum_{k\in \boldsymbol{Kj}} \left(\frac{M_{21,k}}{\rho_k}\right)\right] \tag{7.5}$$

Power required:

$$PW_{ely} = Wsp_{ely} Qc_{ely} \tag{7.6}$$

<u>Bead mill (Bml):</u>

Component release equation:

$$M_{22,k} = Y_{bm} Frc_k M_{17,k};\ \forall k \in \boldsymbol{K^C} \tag{8.1}$$

Residual component (RS) after release:

$$M_{22,RS} = M_{17,B} - \sum_{k\in \boldsymbol{K^C}} M_{22,k} \tag{8.2}$$

Volume of bead mill:

$$Qc_{bml} = \theta_{bml} \left[\sum_{k\in \boldsymbol{Kj}} \left(\frac{M_{22,k}}{\rho_k}\right)\right] \tag{8.3}$$

Power required:

$$PW_{bml} = 13.33\ y_{bml} + 167 Qc_{bml} \tag{8.4}$$

Almost, 60 percent of power dissipates to heat, hence cooling is required.

Cooling water required:

$$Mcw_{bml} Cp_W (Tcw_{out} - Tcw_{in}) = (0.6 PW_{bml}) \tag{8.5}$$

Phase isolation technologies:

<u>Centrifugation (Cnt,2):</u>

Efficiency equations:

$$\eta_{cnt2} = \frac{\sum_{k\in \boldsymbol{K^{H1}}} M_{29,k}}{\sum_{k\in \boldsymbol{K^{H1}}} M_{24,k}} \tag{9.1}$$

$$\eta_{cnt2} = \frac{\sum_{k\in \boldsymbol{K^{L1}}} M_{28,k}}{\sum_{k\in \boldsymbol{K^{L1}}} M_{24,k}} \tag{9.2}$$

Concentration factor (CF_{cnt2}):

$$CF_{cnt2} = \frac{\left[\sum_{k\in \boldsymbol{Kj}}\left(\frac{M_{24,k}}{\rho_k}\right)\right]}{\left[\sum_{k\in \boldsymbol{Kj}}\left(\frac{M_{29,k}}{\rho_k}\right)\right]} \tag{9.3}$$

$$2 \leq CF_{cnt2} \leq 20$$

Sigma factor equation:

$$Qc_{cnt2}U_{g,Deb} = \left[\sum_{k\in \boldsymbol{Kj}}\left(\frac{M_{24,k}}{\rho_k}\right)\right] \tag{9.4}$$

Power required:

$$PW_{cnt2} = Wsp_{cnt2}\left[\sum_{k\in \boldsymbol{Kj}}\left(\frac{M_{24,k}}{\rho_k}\right)\right] \tag{9.5}$$

Power dissipation to heat is about 40%, hence cooling utility is required:

$$Mcw_{cnt2}Cp_W(Tcw_{out} - Tcw_{in}) = (0.4PW_{cnt2}) \tag{9.6}$$

Filtration (Ftt,2):

Retention factor equations:

$$\xi_{k,ftt2} = \frac{M_{31,k}}{M_{25,k}} \; ; \; \forall k \in \; \boldsymbol{K}^{JP} \tag{10.1}$$

Concentration factor (CF_{ftt2}):

$$CF_{ftt2} = \frac{\left[\sum_{k\in \boldsymbol{Kj}}\left(\frac{M_{25,k}}{\rho_k}\right)\right]}{\left[\sum_{k\in \boldsymbol{Kj}}\left(\frac{M_{31,k}}{\rho_k}\right)\right]} \tag{10.2}$$

$$2 \leq CF_{ftt2} \leq 30$$

Flux balance:

$$\zeta_{ftt2}Qc_{ftt2} = \left[\sum_{k\in \boldsymbol{Kj}}\left(\frac{M_{25,k}}{\rho_k}\right)\right]\left(1 - \frac{1}{CF_{ftt2}}\right) \tag{10.3}$$

Power required:

$$PW_{ftt2} = Wsp_{ftt2}Qc_{ftt2} \tag{10.4}$$

Microfiltration (mbr-mf1):

Retention factor equations:

$$\xi_{k,mf1} = \frac{M_{33,k}}{M_{26,k}} \; ; \; \forall k \in \; \boldsymbol{K}^{JP} \tag{11.1}$$

Concentration factor (CF_{mf1}):

$$CF_{mf1} = \frac{\left[\sum_{k\in \boldsymbol{Kj}}\left(\frac{M_{26,k}}{\rho_k}\right)\right]}{\left[\sum_{k\in \boldsymbol{Kj}}\left(\frac{M_{33,k}}{\rho_k}\right)\right]} \tag{11.2}$$

$$1.01 \leq CF_{mf1} \leq 35$$

Flux balance:

$$\zeta_{mf1} Qc_{mf1} = \left[\sum_{k \in \boldsymbol{Kj}} \left(\frac{M_{26,k}}{\rho_k}\right)\right]\left(1 - \frac{1}{CF_{mf1}}\right) \tag{11.3}$$

Power required:

$$PW_{mf1} = Wsp_{mf} Qc_{mf1} \tag{11.4}$$

Differential digestion (ddg):

Digestion agent added:

$$M_{38,Agnt} = \phi_{add,Agnt}\left[\left(\sum_{k \in \boldsymbol{K}^c} M_{36,k}\right) - M_{36,Prd}\right] \tag{12.1}$$

Digestion agent cost:

$$Cpur_{Agnt} = \pi_{Agnt} M_{38,Agnt} \tag{12.2}$$

Costing variables:

$$Qc_{ddg} = \left[\sum_{k \in K^{JP}} \left(\frac{M_{39,k}}{\rho_k}\right)\right] \tag{12.3}$$

Power required:

$$PW_{ddg} = Wsp_{ddg} Qc_{ddg} \tag{12.4}$$

Solubilization (slb):

Solubilizing solvent added:

$$M_{40,Solv} = \phi_{add,Solv} M_{37,Prd} \tag{13.1}$$

Solvent cost:

$$Cpur_{Solv} = \pi_{Solv} M_{40,Solv} \tag{13.2}$$

Costing variables:

$$Qc_{slb} = \left[\sum_{k \in K^{JP}} \left(\frac{M_{41,k}}{\rho_k}\right)\right] \tag{13.3}$$

Power required:

$$PW_{slb} = Wsp_{slb} Qc_{slb} \tag{13.4}$$

Centrifugation (Cnt,3):

Efficiency equations:

$$\eta_{cnt3} = \frac{\sum_{k \in \boldsymbol{K}^{\boldsymbol{DH1}}} M_{46,k}}{\sum_{k \in \boldsymbol{K}^{\boldsymbol{DH1}}} M_{42,k}} \tag{14.1}$$

$$\eta_{cnt3} = \frac{\sum_{k \in \boldsymbol{K}^{\boldsymbol{DL1}}} M_{45,k}}{\sum_{k \in \boldsymbol{K}^{\boldsymbol{DL1}}} M_{42,k}} \tag{14.2}$$

Concentration factor (CF_{cnt3}):

$$CF_{cnt3} = \frac{\left[\sum_{k\in \boldsymbol{Kj}}\left(\frac{M_{42,k}}{\rho_k}\right)\right]}{\left[\sum_{k\in \boldsymbol{Kj}}\left(\frac{M_{46,k}}{\rho_k}\right)\right]} \tag{14.3}$$

$$1.01 \leq CF_{cnt3} \leq 25$$

Sigma factor equation:

$$Qc_{cnt3}U_{g,cnt3} = \left[\sum_{k\in \boldsymbol{Kj}}\left(\frac{M_{42,k}}{\rho_k}\right)\right] \tag{14.4}$$

Power required:

$$PW_{cnt3} = Wsp_{cnt3}\left[\sum_{k\in \boldsymbol{Kj}}\left(\frac{M_{42,k}}{\rho_k}\right)\right] \tag{14.5}$$

Power dissipation to heat is about 40%, hence cooling utility is required:

$$Mcw_{cnt3}Cp_W(Tcw_{out} - Tcw_{in}) = (0.4PW_{cnt3}) \tag{15.6}$$

Reverse osmosis (ro1):

Retention factor equations:

$$\xi_{k,rvs1} = \frac{M_{50,k}}{M_{44,k}}\ ;\ \forall k \in\ \boldsymbol{K^{JP}} \tag{15.1}$$

Concentration factor (CF_{ro1}):

$$CF_{ro1} = \frac{\left[\sum_{k\in \boldsymbol{Kj}}\left(\frac{M_{44,k}}{\rho_k}\right)\right]}{\left[\sum_{k\in \boldsymbol{Kj}}\left(\frac{M_{50,k}}{\rho_k}\right)\right]} \tag{15.2}$$

$$1.01 \leq CF_{ro1} \leq 35$$

Flux balance:

$$\zeta_{ro1}Qc_{ro1} = \left[\sum_{k\in \boldsymbol{Kj}}\left(\frac{M_{44,k}}{\rho_k}\right)\right]\left(1 - \frac{1}{CF_{ro1}}\right) \tag{15.3}$$

Power required:

$$PW_{ro1} = Wsp_{ro1}Qc_{ro1} \tag{15.4}$$

Microfiltration-2 (mf,2):

Retention factor equations:

$$\xi_{k,mf2} = \frac{M_{48,k}}{M_{43,k}}\ ;\ \forall k \in\ \boldsymbol{K^{JP}} \tag{16.1}$$

Concentration factor (CF_{mf2}):

$$CF_{mf2} = \frac{\left[\sum_{k\in \boldsymbol{Kj}}\left(\frac{M_{43,k}}{\rho_k}\right)\right]}{\left[\sum_{k\in \boldsymbol{Kj}}\left(\frac{M_{48,k}}{\rho_k}\right)\right]} \tag{16.2}$$

$$1.01 \leq CF_{mf2} \leq 35$$

Flux balance:

$$\zeta_{mf2} Qc_{mf2} = \left[\sum_{k \in \boldsymbol{Kj}} \left(\frac{M_{43,k}}{\rho_k}\right)\right] \left(1 - \frac{1}{CF_{mf2}}\right) \tag{16.3}$$

Power required:

$$PW_{mf2} = Wsp_{mf} Qc_{mf2} \tag{16.4}$$

Centrifugation (Cnt,4):

Efficiency equations:

$$\eta_{cnt4} = \frac{\sum_{k \in \boldsymbol{K}^{\boldsymbol{SH1}}} M_{55,k}}{\sum_{k \in \boldsymbol{K}^{\boldsymbol{SH1}}} M_{52,k}} \tag{17.1}$$

$$\eta_{cnt4} = \frac{\sum_{k \in \boldsymbol{K}^{\boldsymbol{SL1}}} M_{56,k}}{\sum_{k \in \boldsymbol{K}^{\boldsymbol{SL1}}} M_{52,k}} \tag{17.2}$$

Concentration factor (CF_{cnt4}):

$$CF_{cnt4} = \frac{\left[\sum_{k \in \boldsymbol{Kj}} \left(\frac{M_{52,k}}{\rho_k}\right)\right]}{\left[\sum_{k \in \boldsymbol{Kj}} \left(\frac{M_{55,k}}{\rho_k}\right)\right]} \tag{17.3}$$

$$1.01 \le CF_{cnt4} \le 20$$

Sigma factor equation:

$$Qc_{cnt4} U_{g,cnt4} = \left[\sum_{k \in \boldsymbol{Kj}} \left(\frac{M_{52,k}}{\rho_k}\right)\right] \tag{17.4}$$

Power required:

$$PW_{cnt4} = Wsp_{cnt4} \left[\sum_{k \in \boldsymbol{Kj}} \left(\frac{M_{52,k}}{\rho_k}\right)\right] \tag{17.5}$$

Power dissipation to heat is about 40%, hence cooling utility is required:

$$Mcw_{cnt4} Cp_W (Tcw_{out} - Tcw_{in}) = (0.4 PW_{cnt4}) \tag{17.6}$$

Reverse osmosis (ro2):

Retention factor equations:

$$\xi_{k,rvs2} = \frac{M_{59,k}}{M_{54,k}} \; ; \; \forall k \in \; \boldsymbol{K}^{\boldsymbol{JP}} \tag{18.1}$$

Concentration factor (CF_{ro2}):

$$CF_{ro2} = \frac{\left[\sum_{k \in \boldsymbol{Kj}} \left(\frac{M_{54,k}}{\rho_k}\right)\right]}{\left[\sum_{k \in \boldsymbol{Kj}} \left(\frac{M_{59,k}}{\rho_k}\right)\right]} \tag{18.2}$$

$$1.01 \le CF_{ro2} \le 35$$

Flux balance:

$$\zeta_{ro2} Qc_{ro2} = \left[\sum_{k \in \boldsymbol{Kj}} \left(\frac{M_{54,k}}{\rho_k}\right)\right] \left(1 - \frac{1}{CF_{ro2}}\right) \tag{18.3}$$

Power required:

$$PW_{ro2} = Wsp_{ro2} Qc_{ro2} \tag{18.4}$$

Microfiltration 3 (mf3):

Retention factor equations:

$$\xi_{k,mf3} = \frac{M_{57,k}}{M_{53,k}} \; ; \; \forall k \in \; \boldsymbol{K^{JP}} \tag{19.1}$$

Concentration factor ($\mathrm{CF_{mf3}}$):

$$CF_{mf3} = \frac{\left[\sum_{k \in \boldsymbol{Kj}} \left(\frac{M_{53,k}}{\rho_k}\right)\right]}{\left[\sum_{k \in \boldsymbol{Kj}} \left(\frac{M_{57,k}}{\rho_k}\right)\right]} \tag{19.2}$$

$$1.01 \le CF_{mf3} \le 35$$

Flux balance:

$$\zeta_{mf3} Qc_{mf3} = \left[\sum_{k \in \boldsymbol{Kj}} \left(\frac{M_{53,k}}{\rho_k}\right)\right] \left(1 - \frac{1}{CF_{mf3}}\right) \tag{19.3}$$

Power required:

$$PW_{mf3} = Wsp_{mf} Qc_{mf3} \tag{19.4}$$

Stage-II technologies: Product concentration

Precipitation (prc):

Anti-solvent added for precipitation:

$$M_{62,Ansl} = \phi_{add,Ansl} \sum_{k \in K^{JP}} M_{61,k} \tag{20.1}$$

Solvent cost:

$$Cpur_{Ansl} = \pi_{Ansl} M_{62,Ansl} \tag{20.2}$$

Costing variables:

$$Qc_{prc} = \left[\sum_{k \in \boldsymbol{Kj}} \left(\frac{M_{63,k}}{\rho_k}\right)\right] \tag{20.3}$$

Power required:

$$PW_{prc} = Wsp_{prc} Qc_{prc} \tag{20.4}$$

Microfiltration-4 (mf4):

Retention factor equations:

$$\xi_{k,mf4} = \frac{M_{65,k}}{M_{63,k}} \; ; \; \forall k \in \; \boldsymbol{K^{JP}} \tag{21.1}$$

Concentration factor (CF_{mf4}):

$$CF_{mf4} = \frac{\left[\sum_{k\in Kj}\left(\frac{M_{63,k}}{\rho_k}\right)\right]}{\left[\sum_{k\in Kj}\left(\frac{M_{65,k}}{\rho_k}\right)\right]} \tag{21.2}$$

$$1.01 \le CF_{mf4} \le 35$$

Flux balance:

$$\zeta_{mf4} Qc_{mf4} = \left[\sum_{k\in Kj}\left(\frac{M_{63,k}}{\rho_k}\right)\right]\left(1 - \frac{1}{CF_{mf4}}\right) \tag{21.3}$$

Power required:

$$PW_{mf4} = Wsp_{mf} Qc_{mf4} \tag{21.4}$$

Stage III technologies: Product purification and refinement

Drying (dry):

Solvents and other components removed by sublimation:

$$\sum_{k\in K^{OTP}} M_{67,k} = \kappa c_{dry} \sum_{k\in K^{OTP}} M_{66,k} \tag{22.1}$$

Product not sublimed in freeze drying:

$$M_{68,Prd} = M_{66,Prd} \tag{22.2}$$

Energy required for sublimation:

$$E_{sub} = \sum_{k\in Kj} M_{67,k} \left[\left(Cp_k \left|T_{frz} - T_{amb}\right|\right) + \lambda_{sub,k}\right] \tag{22.3}$$

Refrigerant required for freezing:

$$M_{rfrg} Cp_{rfrg} \left|Trfrg_{out} - Trfrg_{in}\right| = E_{sub} \tag{22.4}$$

Area of dryer:

$$UA_{dry} A_{dry} \left|Trfrg_{out} - T_{amb}\right| = E_{sub} \tag{22.5}$$

Sublimation capacity – costing variable:

$$Qc_{dry} = \sum_{k\in Kj} M_{67,k} \tag{22.6}$$

Power required in dryer:

$$PW_{dry} = Wsp_{dry} A_{dry} \tag{22.7}$$

Final desired product conditions:

Product purity:

$$M_{68,Prd} \ge Pur \sum_{k\in Kj} M_{68,k} \tag{23.1}$$

Final product amount:

$$\sum_{k\in Kj} M_{68,k} \ge Prd_F \tag{23.2}$$

Equations for evaluating stage-wise categorical costs:

Annualized capital costs in each stage (annualized)

$$CCAC_{Nstg} = 1.66\, \varsigma_{RF} BMC_{mult} \sum_{i \in istg\{1,2,3\}} Ce_i \quad (24.1)$$

Material costs in stages:

$$CCRM_{s1} = \left[\tau_{ann}\left(Cpur_{Flcnt} + Cpur_{Acid} + Cpur_{Enz} + Cpur_{Solv} + Cpur_{Agnt}\right)\right] \quad (24.2a)$$

$$CCRM_{s2} = \left[\tau_{ann}\left(Cpur_{Ansl}\right)\right] \quad (24.2b)$$

$$CCRM_{s3} = 0 \quad (24.2c)$$

Consumable costs in each stage:

$$CCRM_{s1} = Cons_{ftt1} + Cons_{ftt2} + Cons_{mf1} + Cons_{mf2} + Cons_{mf3} + Cons_{rvs1} + Cons_{rvs2} \quad (24.3a)$$

$$CCRM_{s2} = Cons_{mf4} \quad (24.3b)$$

$$CCRM_{s3} = 0 \quad (24.3c)$$

Labor costs in each stage:

$$CCLB_{Nstg} = \tau_{ann}\, C_{Lab} \sum_{i \in istg\{1,2,3\}} Nlb_i \quad (24.4)$$

Utility costs in each stage:

$$CCUT_{Nstg} = \tau_{ann} \begin{bmatrix} \left(C_{elec} \sum_{i \in istg\{1,2,3\}} PW_i\right) + \left(C_{cwt} \sum_{i \in istg\{1,2,3\}} Mcwt_i\right) \\ + \left(C_{stm} \sum_{i \in istg\{1,2,3\}} Mstm_i\right) + \left(C_{rfrg} \sum_{i \in istg\{1,2,3\}} Mrfrg_i\right) \end{bmatrix} \quad (24.5)$$

Total cost in each stage:

$$CCTC_{Nstg} = CCAC_{Nstg} + CCRM_{Nstg} + CCCS_{Nstg} + 2.78\, CCLB_{Nstg} + CCUT_{Nstg} \quad (24.6)$$

Other costs in each stage:

$$CCOT_{Nstg} = CCTC_{Nstg} - \{CCAC_{Nstg} + CCRM_{Nstg} + CCCS_{Nstg} + CCLB_{Nstg} + CCUT_{Nstg}\} \quad (24.7)$$

Total costs in different categories:

$$CCTFC = \tau_{ann}\, C_{Feed} \quad (25.1)$$

$$CCTAC = \sum_{n \in Nstg} CCAC_n \quad (25.2)$$

$$CCTRM = \sum_{n \in Nstg} CCRM_n \quad (25.3)$$

$$CCTCS = \sum_{n \in Nstg} CCCS_n \quad (25.4)$$

$$CCTLB = \sum_{n \in Nstg} CCLB_n \quad (25.5)$$

$$CCTUT = \sum_{n \in Nstg} CCUT_n \quad (25.6)$$

$$CCTOT = \sum_{n \in Nstg} CCOT_n \quad (25.7)$$

Total process cost:

$$CCTPC(\$/annum) = CCTFC + CCTAC + CCTRM + CCTCS + CCTLB + CCTUT + CCTOT \quad (26)$$

Objective function:

$$Obj \; = \; Min\, CCTPC \quad (27)$$

Overall process cost per unit product:

$$UPC(\$/kg) = \frac{CCTPC}{\tau_{ann} Prd_F} \quad (28)$$

B.3 Model parameters and input data for base case:

Table B.3.1 Important input parameters and product specifications

Parameter	Nominal value	Units
Initial cell titer	5	g/L (kg/m3)
Cell diameter	10	µm
Product content in cells	25	wt% of cell dry weight (CDW)
Desired production capacity	1000	kg/h
Annual operation time	330	days/year
Final product purity	95	wt% purity

Table B.3.2 Cell dry weight composition:

Component	Mass composition (wt %)
Product	25%
Carbohydrate	28%
Protein	13%
RNA	13%
Lipid	7%
Cell wall material	10%
Other	4%

Table B.3.3 Component density data:

Component	Density (kg/m3)
Water	1000
Biomass	1100
Product	2000
Carbohydrate	1100
Protein	1850
RNA	1030
Lipid	850
Debris	1950
Other	0.04

Table B.3.4 Utility and labor costs: (SuperPro Designer v8.5)

Utility	Cost per unit ($/unit)
Electricity	0.1 $/KWH
Cooling water	5E-5 $/kg
Steam	0.012 $/kg
Labor	30 $/laborer-h

B.3.5 Flocculation (Flc):

Flocculent added – 0.04 kg/m3; Flocculent cost – 5 $/kg
Floc diameter – 10E^{-5} m (5 times increase in size)

B.3.6 Sedimentation tank (Sdm):
Efficiency – 70%
Depth – 3m

B.3.7 Centrifuge (Cnt):

Parameter/Technology	Cnt1	Cnt2, Cnt3, Cnt4
Efficiency	80%	85%
Rotation speed	9000 rpm	12000 rpm

B.3.8 Filtration (Ftt):
Flux: 0.2 $m^3m^{-2}h^{-1}$
Retention factors (Ftt1): Biomass – 80%, Water – 20%
Retention factors (Ftt2): product – 80%, Water – 20%, other solid components (protein, debris) – 80%, liquids like acid and enzyme – 20%, light liquids and soluble components (lipids, others) – 20%
Filter cost – 100 $/m^2$
Replacement time – 2000 h

B.3.9 Bead mill (Bml):
Product release – 85% (k – 0.02, phi – 0.82)

B.3.10 Enzyme lysis (Ely):
Enzyme addition – 0.02 kg/kg biomass
Enzyme cost – 50 $/kg
Density enzyme – 1150 kg/m^3
Product release – 90%

B.3.11 Acid hydrolysis (Ahy):
Acid addition – 0.1 kg/kg solution (achieve 0.02 N)
Acid cost – 0.1 $/kg
Density enzyme – 1840 kg/m^3
Product release – 85%
Temperature for lysis – 120 ^{0}C

B.3.12 Microfiltration (MF,1, 2, 3 and 4):
Flux – 0.0856 $m^3m^{-2}h^{-1}$
Retention factor: light liquids and water – 0.15, heavy solid (product, debris, proteins) – 0.85, solvents – 0.15.
Microfilter membrane cost – 736 $/m^2$
Replacement time – 2000 h

B.3.13 Reverse osmosis (RO-1,2):
Flux – 0.025 $m^3m^{-2}h^{-1}$
Retention factor: light liquid components and water – 0.1, heavy solid (product, debris, proteins) – 0.90, solvents like enzyme, acid – 0.1.
RO Membrane cost – 350 $/m^2$
Replacement time – 2000 h

B.3.14 Differential digestion (Ddg):
Agent required – 0.4 kg/kg NPCM (non-product cellular materials)
Cost of agent – 0.6 $/kg
Density of agent – 1100 kg/m^3

B.3.15 Solubilization (Slb):
Solvent required – 0.4 kg/kg product
Cost of solvent – 0.6 $/kg
Density of solvent – 1800 kg/m^3

B.3.16 Precipitation (Prc)
Efficiency of product precipitation – 95%
Anti-solvent required – 2 kg/kg product
Cost of anti-solvent – 0.3 $/kg
Density of anti-solvent – 1000 kg/m^3

B.3.17 Freeze drying (Dry):
Sublimation of solvents – 97%
Heat of sublimation – 5000 KJ/kg
Ambient temperature: 20 ^{0}C
Freezing temperature: (-1) ^{0} C
Refrigerant inlet temperature – (-10) ^{0} C
Refrigerant outlet temperature – 0 ^{0} C
Specific heat (KJ/kg-^{0}C): Refrigerant – 12, water – 4.2, other liquids – 1
Heat transfer coefficient – 180 KJ/m^2-K-h

B.3.18 Table for standard capacity, costs, scaling factors, labor requirements for technologies:

Unit operation (costing capacity)	Standard capacity (units)	Base costs (million $)	Scaling exponent (n)	Laborers required (#/h)	Power required (KW/h)
Flocculation (Volume)	2000 m^3	0.538	0.5	0.1	0.0002
Sedimentation (Area)	2500 m^2	1.128	0.57	0.1	0
Centrifuge1 (Sigma factor)	60000 m^2	0.275	0.65	1	12.79
Filtration (Area)	80 m^2	0.039	0.55	0.5	0.1
Bead milling (Volume)	0.275 m^3	0.272	0.95	0.5	(calculated from eq. 8.4 in B.2.6)
Acid hydrolysis (Flowrate)	80 m^3/h	0.379	0.5	1	0.1
Microfiltration (Area)	80 m^2	0.75	0.55	1	0.1
Differential digestion (Flowrate)	40 m^3/h	0.474	0.5	1	0.1
Solubilization (Flowrate)	40 m^3/h	0.474	0.5	1	0.1
Centrifuge (2,3,4) (Sigma factor)	60000 m^2	0.66	0.65	1	19.2
Reverse osmosis (Area)	80 m^2	0.234	0.55	1	0.35
Precipitation (Flowrate)	40 m^3/h	0.474	0.5	1	0.1
Freeze drying (Capacity)	600 kg/h	0.107	0.67	0.5	0.3

B.4 Integer-cuts for alternate configurations

Integer cut (a. k. a. no good integer cut) is used to find the near best separation configuration [13]. The utility of this implementation is to; (i) identify alternate technologies and separation configurations which can be used if the optimal configuration cannot be implemented due to some constraints, and (ii) to what extent is the overall process cost affected by the change in configurations. This is an iterative method

where we keep adding integer cuts formulated from the previous solutions to the current optimization problem, and solve it to find the next best solution. The equation (29) shows the generalized formula for an integer cut.

$$\sum_{y_{ibv}=1} y_{ibv} - \sum_{y_{ibv}=0} y_{ibv} \leq (\text{\# of 1's in solution}) - 1 \qquad (29)$$

Thus, the successive integer cuts added to the optimization model for study case I are:

(1) Integer cut#1:

$$\left[y_{cnt1} + y_{ahy} + y_{cnt2} + y_{ddg} + y_{cnt3}\right] - \begin{bmatrix} y_{sdm} + y_{ftt1} + y_{ely} + y_{bml} + y_{mf1} + y_{ftt2} \\ +y_{byp} + y_{slb} + y_{mf2} + y_{ro1} + y_{slb} + y_{mf3} \\ +y_{ro2} + y_{cnt4} + y_{prc} + y_{mf4} \end{bmatrix} \leq (5) - 1 \qquad (29.1)$$

(2) Integer cut#2:

$$\begin{bmatrix} y_{cnt1} + y_{ahy} + y_{cnt2} + y_{slb} \\ +y_{cnt4} + y_{prc} + y_{mf4} \end{bmatrix} - \begin{bmatrix} y_{sdm} + y_{ftt1} + y_{ely} + y_{bml} + y_{mf1} \\ + y_{ftt2} + y_{byp} + y_{ddg} + y_{cnt3} + y_{mf2} \\ +y_{ro1} + y_{mf3} + y_{ro2} \end{bmatrix} \leq (7) - 1 \qquad (29.2)$$

(3) Integer cut#3:

$$\left[y_{cnt1} + y_{ely} + y_{cnt2} + y_{ddg} + y_{cnt3}\right] - \begin{bmatrix} y_{sdm} + y_{ftt1} + y_{ahy} + y_{bml} + y_{mf1} + y_{ftt2} \\ +y_{byp} + y_{slb} + y_{mf2} + y_{ro1} + y_{slb} + y_{mf3} \\ +y_{ro2} + y_{cnt4} + y_{prc} + y_{mf4} \end{bmatrix} \leq (5) - 1 \qquad (29.3)$$

B.5 Additional results from study -1

We present some additional results from the analysis performed for case study-1 in the following sub-sections. These results support the major results presented in the paper and help in understanding the overall approach and efforts used in the analysis.

B.5.1 Stage-wise categorical cost contributions

The stage-wise categorical (annualized capital, materials, consumables, utilities, labor and others) cost contributions for the base case is shown in Figure B.4. It can be seen that materials (~42%) and utilities (~41%) are major contributors in stage-I. The material costs are high because of flocculent addition in flocculation (Flc), acid required in acid hydrolysis (Ahy) and digestion agent added in differential digestion (Ddg) technologies. However, flocculation is a pretreatment technology required for efficient cell harvesting and the amount of flocculent added is very low (lower cost contribution) as compared to the amounts of acid and digestion agent (higher cost contribution). The utility requirements are high because of centrifuge (Cnt,1,2,3) selection, an energy intensive process. Also, high speed of rotation is required to achieve a reasonable centrifugal to gravitational force ratio, which causes heat dissipation and cooling is required to maintain normal temperature conditions. In stage-III, the utility costs are high (~44%) because of the sublimation agent used in drying (Dry).

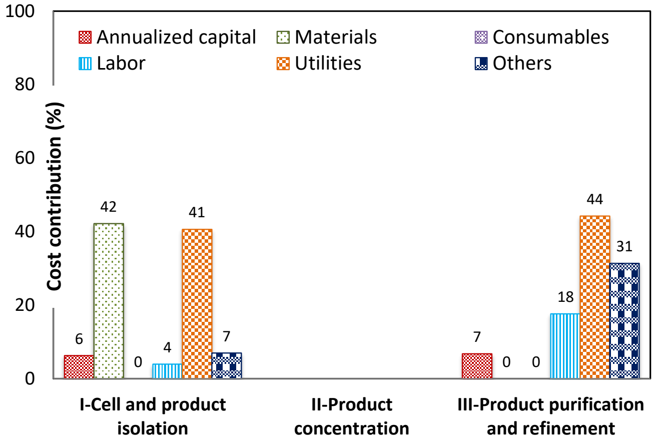

Figure B.4. Cost contribution by different categories (annualized capital, materials, consumables, labor, utilities and other costs) in the three separation stages.

B.5.2 Plots for cell harvesting performance indices

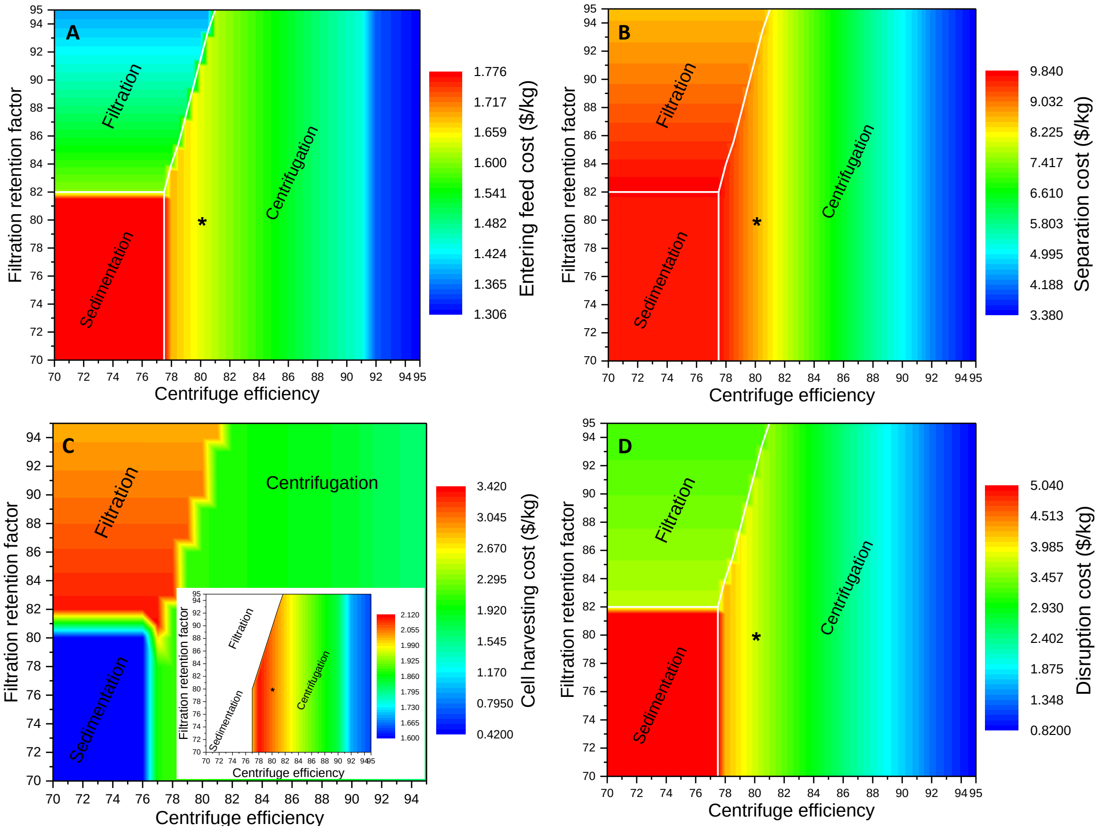

Figure B.5. Variation in the (A) Entering feed cost, (B) Separation cost, (C) Cell harvesting cost, and (D) Cell disruption cost, for a range of performance index values for centrifuge (efficiency) and filtration (retention factor) technologies used for cell harvesting. The white lines denote the change in unit selection and the black marker corresponds to the base case (80, 80), where the centrifuge efficiency and filtration retention factor are both 80%.

It is evident from plot A that entering feed is more for sedimentation. The feed costs decrease significantly with the increase in performance indices of centrifuge as well as filtration. Separation costs (plot B) follow a similar trend as the overall process costs. The harvesting cost (plot C) is lowest for sedimentation,

followed by centrifugation and highest for filtration. The cost variation for the region where centrifuge is selected is highlighted in a smaller plot in plot C to show a clear change in the costs. The disruption cost (plot D) depends on the amount of materials handled in the unit (as acid hydrolysis is the selected technology in all scenarios) and hence is highest for sedimentation due to its low efficiency in harvesting. The disruption costs vary based on the performance indices of centrifuge and filtration.

B.5.3 Plots for cell disruption performance indices

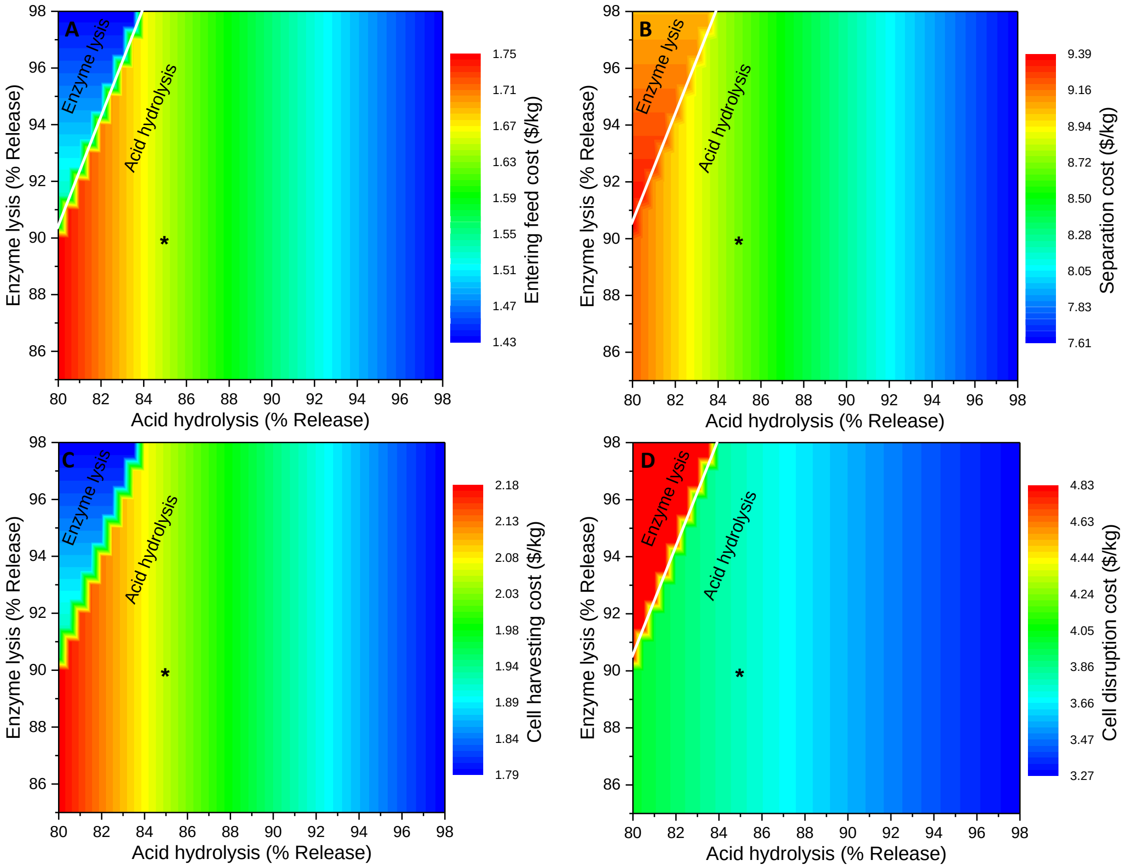

Figure B.6. Variation in the (A) Entering feed cost, (B) Separation cost, (C) Cell harvesting cost, and (D) Cell disruption cost, for a range of performance index values for acid hydrolysis (% release) and enzyme lysis (% release) technologies used for cell disruption. The white lines denote the change in unit selection and the black marker corresponds to the base case (85, 90), where the acid hydrolysis release is 85% and enzyme lysis release is 90%.

For variation in performance indices for cell disruption technologies, the bead mill option is not competitive when compared to acid hydrolysis and enzyme lysis. It is evident from plot A that entering feed is more for acid hydrolysis. The feed costs decrease significantly with the increase in performance indices of acid hydrolysis and enzyme lysis. Separation costs (plot B) follow a similar trend as the overall process costs. The harvesting cost (plot C) is low in the region where enzyme lysis is selected. The disruption cost (plot D) is high for enzyme lysis (due to high enzyme costs) as compared to acid hydrolysis (acid is cheaper than enzyme).

B.5.4 Plots for matching parameter for phase isolation

We observe that the cost of separation (plot A) increases as the amount of digestion agent or solubilizing solvent increases. The stage-II is absent when differential digestion is selected (plot B). This shows that we need to find better digestion agents with low requirements and low costs to enhance the separation process as well as lower the costs.

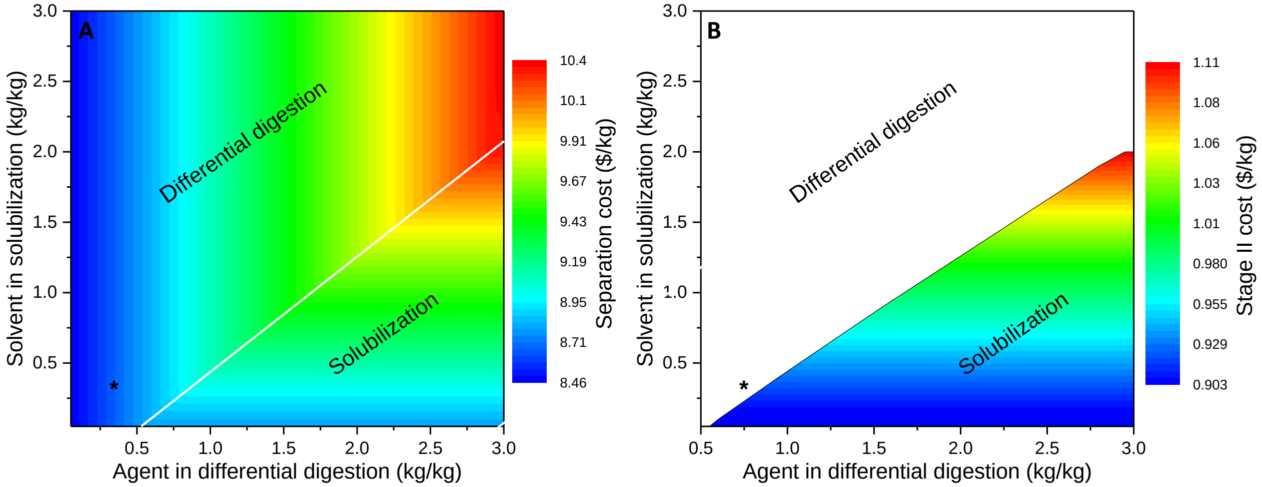

Figure B.7. Variation in the (A) Separation cost, and (B) Stage II cost, for a range of values for agent and solvent addition (kg/kg biomass) in differential digestion and solubilization respectively. The white lines and the white region denote the change in unit selection and the black marker corresponds to the base case (0.4, 0.4).

B.5.5 Plots for varying feed costs

The feed cost (a. k. a. the effluent for bioreactors) can vary based on the system in upstream processing. The nutrient and substrates used for microbial growth and product formation can come from different sources, hence causing a variation in the feed cost. We assume a cost based on unit product in the downstream separation system and hence this number is different than the actual entering feed costs as this also includes losses due to limitations in performance indices (less than 100% in all cases) of the separation technologies. The overall process cost increases with the increase in feed cost however, the separation cost remains constant. The separation technologies selected in all cases are the same (base case configuration) hence there are no changes. The feed cost increase affects the actual entering feed costs and hence the increase in overall process costs.

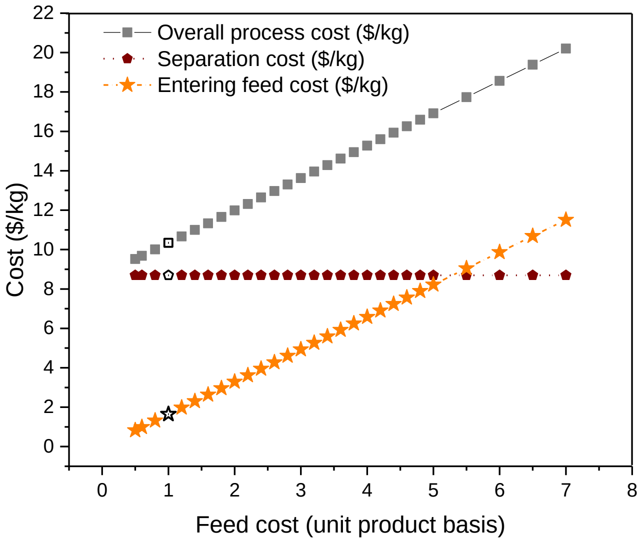

Figure B.8. Effect of variation in feed costs (kg feed/kg product) on the overall process cost, separation cost and actual entering feed costs. The feed cost on the x-axis is an assumed number and the actual entering feed cost is different because of some losses during product recovery and purification in the technologies used.

C. Case study 2 – Intracellular soluble product

C.1 Separation scheme and superstructure for intracellular soluble product

The general separation scheme for all intracellular (IN) soluble (SOL) products is presented in Figure C.1.

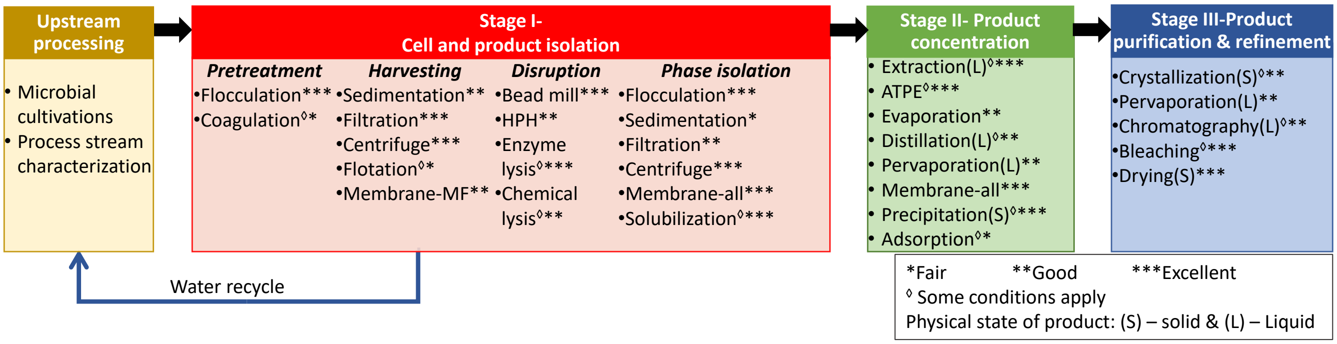

Figure C.1 Separation scheme for intracellular (IN) and soluble (SOL) product. The color scheme denotes the three stages: stage I – red, stage II –green and stage III – blue. The technology suitability has been divided into grades - fair (*), good (**) and excellent (***), if applicable under certain conditions (◊) and physical state of the product (solid (S) and liquid (L)).

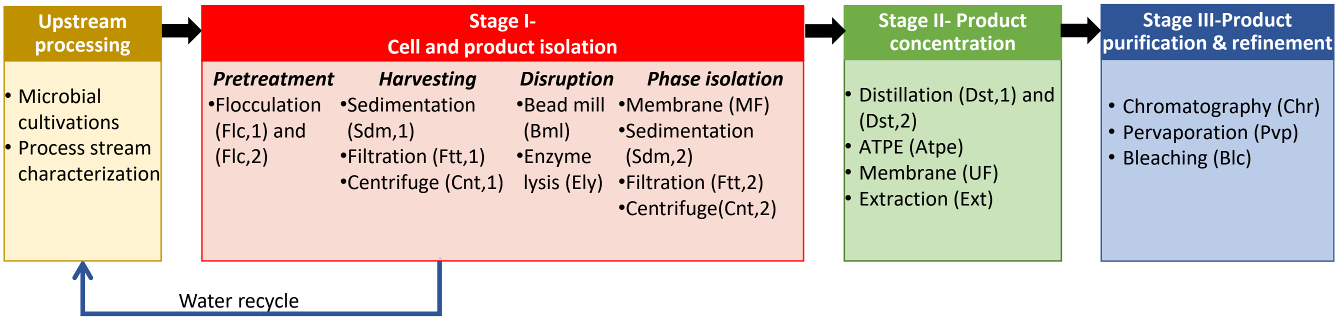

Figure C.2 Separation scheme for product class intracellular-soluble-liquid-volatile-specialty (IN-SOL-LQD-VOL-SPC) chemical. The reduced list of relevant technology options in each stage are listed in the corresponding boxes.

We reduce the list of technologies based on additional product properties such as liquid (LQD), volatile (VOL), and specialty (SPC) grade, considered for study case II. The simplified scheme for the additional property descriptors is shown in Figure C.2. The separation superstructure for the class IN-SOL-LQD-VOL-SPC is shown in Figure C.3.

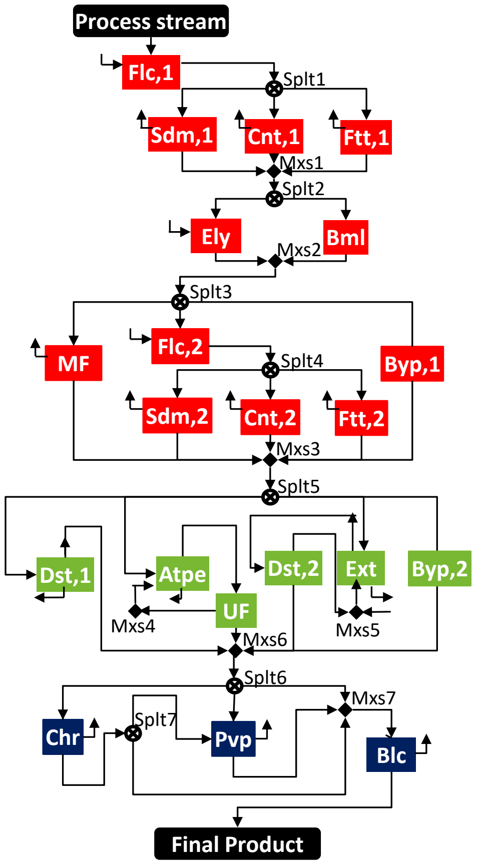

Figure C.3 Separation superstructure for intracellular (IN) - soluble (SOL) - liquid (LQD) - volatile (VOL) - specialty (SPC) chemical. The three stages are distinguished based on colors: (I) Cell and product isolation - red (II) Product concentration – green (III) Product purification and refinement – blue. The technologies involved are flocculation (Flc,1,2), sedimentation (Sdm,1,2), centrifugation (Cnt,1,2), filtration (Ftt,1,2), enzyme lysis (Ely), bead milling (Bml), membrane processes (microfiltration (MF) and ultrafiltration (UF)), distillation (Dst1 and Dst2), aqueous two phase extraction (Atpe), extraction (Ext), chromatography (Chr), pervaporation (Pvp) and bleaching (Blc). Options for bypassing (Byp) a stage or some tasks are also included.

C.2 Model details and equations

C.2.1 Indices and sets

$i \in \boldsymbol{I}$ - technologies (used as subscript to variables)

{ *flc#* - flocculation and #={1,2},

sdm – sedimentation and #={1,2},

splt# and *mxs#* - splitters and mixers,

cnt# - centrifugation and # = {1, 2,},

ftt# - filtration and # = {1, 2},

ely - enzyme lysis (cell disruption by biological method)

bml - bead mill (cell disruption by mechanical method)

mbr - membrane technologies, MF (*mf*) – microfiltration and UF (*uf*) – ultrafiltration

byp# - bypass

dst# - distillation and # = {1, 2},

atpe – aqueous two phase extraction

ext – liquid-liquid extraction

chr – chromatography

pvp – pervaporation

blc – bleaching}

$j \in \boldsymbol{J}$ – streams (used as subscript to variables)

{1, 2, 3, 4,…………………, 72}

$k \in \boldsymbol{K}$ – components (includes the following - used as subscript to variables)

{*B* – biomass, *W* – water, *Deb* – cell debris, *Prd* – desired product, *Slb* – soluble co-product, *RS* – residual solids (unreleased cell components), *Flcnt1*, *Flcnt2* – Flocculants added, *Enz* – enzyme added, *Solv* – solvent in ***ext***, *Salt* – salt in ***atpe***, *Poly* – polymer in ***atpe***}

$s \in \boldsymbol{NS}$ – stages {*s1, s2, s3*}

$l \in \boldsymbol{L}$ – utilities {electricity (*elec*), steam (*stm*), cooling water (*cwt*)}

C.2.2 Subsets

Subsets for technologies

$\boldsymbol{I}^{\mathbf{CST}}$ – technologies with costs

{*flc1, sdm1, cnt1, ftt1, ely, bml, flc2, sdm2, cnt2, ftt2, mf, dst1, atpe, uf, ext, dst2, chr, pvp, blc*}

$\boldsymbol{I}^{\mathbf{CF}}$ – technologies with concentration factor

{*sdm1, sdm2, cnt1, cnt2, ftt1, ftt2, mf, uf, pvp*}

$\boldsymbol{I}^{\mathbf{CONS}}$ – technologies having consumables

{*ftt1, ftt2, mf, uf, pvp, blc*}

$\boldsymbol{I}^{\boldsymbol{BV}}$ – technologies having binary selection constraints

{*sdm1, cnt1, ftt1, ely, bml, flc2, sdm2, cnt2, ftt2, mf, dst1, atpe, uf, ext, dst2, chr, pvp,byp#*}

$\boldsymbol{I}^{\boldsymbol{S1}}$ – technologies in stage I

{*flc1, sdm1, cnt1, ftt1, ely, bml, flc2, sdm2, cnt2, ftt2, mf*}

$\boldsymbol{I}^{\boldsymbol{S2}}$ – technologies in stage II

{*dst1, atpe, uf, ext, dst2*}

$\boldsymbol{I}^{\boldsymbol{S3}}$ – technologies in stage III

{*chr, pvp, blc*}

Subsets for streams

$\boldsymbol{J}^{\boldsymbol{BD}}$ – streams before disruption {1 to 13}

$\boldsymbol{J}^{\boldsymbol{AD}}$ – streams after disruption {19 to 72}

$\boldsymbol{J}^{\boldsymbol{dst}}$ – streams in distillation {38, 42, 43, 52, 54, 55}

$\boldsymbol{J}^{\boldsymbol{dst1}}$ and $\boldsymbol{J}^{\boldsymbol{dst2}}$ – streams in distillation #1{38, 42, 43} and #2 {52, 54, 55} respectively

Subsets for components

K^{JP} – components in process streams

{*B, W, Prd, Deb, Slb, RS, Enz, Solv, Salt, Poly*}

K^{BD} – components before disruption

{*B, W*}

K^{C} – components in cell or biomass

{*Prd, Slb, Deb*}

K^{ADD} – added components

{*Flcnt1, Flcnt2, Enz, Solv, Salt, Poly*}

K^{L1} – light components in stage-1

{*W, Prd, Slb*}

K^{H1} – heavy components in stage-1

{*Deb, RS, Enz*}

K^{dst} – components in distillation #{1,2}

{*W, Enz, Prd, Slb, Deb, RS, Solv*}

K^{dst1} – components in *dst1*

{*W, Enz, Prd, Slb, Deb, RS*}

K^{dst2} – components in *dst2*

{*W, Enz, Prd, Slb, Deb, RS, Solv*}

Kp^{atpe} – solutes in ATPE

{*Prd, W, Slb, Deb, RS, Enz*}

Kp^{ex} – solutes in extraction

{*Prd, Slb, Deb, Enz, RS*}

K^{SOL} – added solvents

{*Solv, Salt, Poly*}

K^{NS} – non-solvent components

{*Prd, W, Slb, Deb, RS, Enz*}

K^{OTP} – components other than product

{*W, Deb, Slb, RS, Enz, Solv, Salt, Poly*}

C.2.3 Dynamic sets for connectivity

To denote connection between technologies, streams and components (*Yes* if connected, *No* if not)

J_i – streams of technology *i*

Jin_i – inlet streams of technology *i*

$Jout_i$ – outlet streams of technology *i*

K_i – components *k* in technology *i*

$\boldsymbol{K_j}$ – components k in stream j

C.2.4 Model Parameters

General parameters

$\alpha_{k,i}$ = Relative volatility of component k for technology i {*dst1* and *dst2*}

Υ_i (%) = Release parameter in disruption technologies

ζ_i (m^3/m^2h) = Flux of technology i {*ftt* and *mbr* technologies}

η_i(%) = Efficiency of technology i

$\theta_i(h)$ = Residence time in technology i

θ_i^{Rep}(h/year) = Replacement time for consumables in technology i

κPT_k = Partition of component k in top phase in *atpe*

κP_k = Partition of component k in solvent in *ext*

λ_{stm}(KJ/kg) = Latent heat of steam

$\lambda^{vap}{}_k$(KJ/kg) = Heat of vaporization of component k

$\xi_{k,i}$(——) = Retention factor of component k for technology i {*ftt* and *mbr* technologies}

π_{feed}(\$/kg biomass) = Entering feed cost in terms of per kg biomass

$\pi^{Rep}{}_i$ (\$/unit) = Replacement cost of consumables per unit capacity in technology i

$\pi_{k/l}$ (\$/unit) = Unit price of component k or utility l

ρ_k(kg/m^3) = Density of component k

ς_{RF}(——) = Capital recovery/charge factor (0.11)

τ_{ann}(h/annum) = (330 day x 24 h/day = 7920)

$\phi_{add,k}$ (kg/kg) = Addition amounts for flocculants and enzyme

$\psi_{k-k'}$(kg/kg) = Solubility of component k in k'

BMC_{mult}(——) = Bare module cost multiplier (5.4)

C_{bio}(kg/m^{3}or g/L) = Biomass titer

C_{Lab}(\$/h) = Labor cost - operator basis (30)

Cp$_k$(KJ/kg-°C) = Specific heat of component k

$C0_i$(\$/capacity) = Cost of a technology with standard capacity

d_p(m) = Particle diameter

d_{floc}(m) = Floc diameter

Frc_k (——)= Fraction of cellular component k in biomass

g(m/s^2) = Acceleration due to gravity

$M1_k$(——) = Big-M constant for component k

MW_k(kg/mol) = Molecular weight of component k

nc_i(——) = cost scaling index for technologies

$Nlabr_i(\#/h)$ = # of laborers required for technology i per hour

Prd_F(kg/h) = Final product recovered per hour (500)

Pur (%) = purity of product (99%)

$Q0_i$(m^3 or m^2 or m^3/h) = Standard capacity of a technology for costing, labor and power required (varies – details in **Table B.3.18** in section B.3)

T_{amb} (°C) = Ambient temperature (25)

T_{sat}(°C) = Saturation temperature (100)

Tcw_{in}(°C) = Cooling water temperature in (25)

Tcw_{out} (°C) = Cooling water temperature out (30)

$Wsp_i(kW/h)$ = Power required by technology *i* per hour

Evaluated parameters

ω_i(rpm) = angular velocity in centrifuge technologies *i* = {*cnt1, cnt2*}

SOR_i(m/s) = surface overflow rate in sedimentation, *i* = {*sdm1, sdm2*}

$U_{g,Floc,i}$(m/s) = settling velocity of flocs, *i* = {*flc1, flc2*}

$U_{g,i}$(m/s) = settling velocity in centrifuge technologies *i* = {*cnt1, cnt2*}

C.2.5 Model Variables

General variables

Ce_i($/capacity) = Purchase cost for technologies $i \in \boldsymbol{I^{CST}}$

CF_i($\mathrm{m}^3/\mathrm{m}^3$) = Concentration factor for technologies $i \in \boldsymbol{I^{CF}}$

C_{Feed}($/h) = Purchase cost of feed stream

$Cons_i$($/annum) = Consumable costs for technologies $i \in \boldsymbol{I^{CONS}}$

$Cpur_k$($/h) = Purchase cost of added components ($k \in \boldsymbol{K^{ADD}}$)

$F_{jdst,kdst}$(Kmol/h) = Molar flows of components in distillation

$M_{j,k}$(kg/h) = Mass flowrate of component *k* in stream *j*

Mcw_i(kg/h) = Amount of cooling water required for technologies $i \in \boldsymbol{I^{CST}}$

$Mstm_i$(kg/h) = Amount of steam required for technologies $i \in \boldsymbol{I^{CST}}$

Nlb_i(#/h) = Number of laborers required for technologies $i \in \boldsymbol{I^{CST}}$

PW_i(KW/h) = Power required for technologies $i \in \boldsymbol{I^{CST}}$

Qc_i(m^3 or m^2 or m^3/h) = Costing variable for technologies $i \in \boldsymbol{I^{CST}}$

QW(m^3/h) = Entering stream flowrate

$Xm_{jdst,kdst}$ $(-)$ = Mole fraction of components in distillation

Stage-wise costing variables:

$CCAC_{Nstg}$= Fixed (annualized capital) cost in nth stage

$CCCS_{Nstg}$= Consumables cost in nth stage

$CCLB_{Nstg}$= Labor cost in nth stage

$CCOT_{Nstg}$= Other cost in n^th stage (overheads and supervision costs)

$CCRM_{Nstg}$= Materials cost in n^th stage

$CCTC_{Nstg}$= Total cost in n^th stage (all costs added)

$CCUT_{Nstg}$= Utility cost in n^th stage

$CCTAC$, $CCTCS$, $CCTFC$, $CCTLB$, $CCTOT$, $CCTPC$, $CCTRM$, $CCTUT$ = Total costs for the different categories for the complete separation process

Binary variables

$y_i(--)$ = Binary variables for technologies to be selected $i \in \boldsymbol{I^{BV}}$

C.2.6 Model Equations

Initial flow assignment equations:

$$M_{1,W} = \rho_w QW \tag{1.1a}$$

$$M_{1,B} = C_{bio} QW \tag{1.1b}$$

Component balances in all technologies:

$$\sum_{j \in \boldsymbol{Jin_i}} M_{j,k} = \sum_{j \in \boldsymbol{Jout_i}} M_{j,k} \; ; \; \forall \, k \in \boldsymbol{K^{JP}} \; and \; i \in \boldsymbol{I} \tag{1.2}$$

Cost of entering feed:

$$C_{Feed} = \pi_{feed} M_{1,B} \tag{1.3}$$

Cost of technologies:

$$\left(\frac{Ce_i}{C0_i}\right) = \left(\frac{Qc_i}{Q0_i}\right)^{nc_i} \; ; \; \forall i \in \boldsymbol{I^{CST}} \tag{1.4}$$

Labor requirement in technologies:

$$Nlb_i Q0_i = Nlabr_i Qc_i \; ; \; \forall i \in \boldsymbol{I^{CST}} \tag{1.5}$$

Consumable costs in technologies:

$$Cons_i = \frac{\tau_{ann}}{\theta_i^{Rep}} \pi^{Rep}{}_i Qc_i \; ; \; \forall i \in \boldsymbol{I^{CST}} \tag{1.6}$$

Logical equations:

$$M_{j,k} - M1_k y_{ibv} \le 0 \; ; \; \forall \, i \in \boldsymbol{I^{BV}}, j \in \boldsymbol{J}, k \in \; \boldsymbol{K_i} \; and \; \boldsymbol{K_j} \tag{1.7}$$

Technology selection equations:

<u>Stage-I technologies:</u>

Harvesting technologies:

$$y_{sdm1} + y_{cnt1} + y_{ftt1} = 1 \tag{1.8}$$

Cell disruption technologies:

$$y_{ely} + y_{bml} = 1 \tag{1.9}$$

Phase isolation technologies:

$$y_{flc2} + y_{mf} + y_{byp1} = 1 \quad (1.10)$$

$$y_{sdm2} + y_{cnt2} + y_{ftt2} = y_{flc2} \quad (1.11)$$

Stage-II technologies:

$$y_{dst1} + y_{atpe} + y_{ext} + y_{byp2} = 1 \quad (1.12)$$

$$y_{uf} = y_{atpe} \quad (1.13)$$

$$y_{dst2} = y_{ext} \quad (1.14)$$

Stage-III technologies:

$$y_{chr} + y_{byp3} = 1 \quad (1.15)$$

$$y_{pvp} + y_{byp4} = 1 \quad (1.16)$$

Stage-I models: Flocculation, cell harvesting, cell disruption and phase isolation technologies

Flocculation1 (Flc,1):

Flocculent added:

$$M_{2,Flcnt1} = \phi_{add,\mathrm{Flcnt1}} \left[\sum_{k \in \boldsymbol{K}^{\boldsymbol{BD}}} \left(\frac{M_{1,k}}{\rho_k}\right)\right] \quad (2.1)$$

Flocculent cost:

$$Cpur_{Flcnt1} = \pi_{Flcnt1} M_{2,Flcnt1} \quad (2.2)$$

Volume of flc1:

$$Qc_{flc1} = \theta_{flc1} \left[\sum_{k \in \boldsymbol{K}^{\boldsymbol{BD}}} \left(\frac{M_{1,k}}{\rho_k}\right)\right] \quad (2.3)$$

Power required:

$$PW_{flc1} = Wsp_{flc1} \left[\sum_{k \in \boldsymbol{K}^{\boldsymbol{BD}}} \left(\frac{M_{1,k}}{\rho_k}\right)\right] \quad (2.4)$$

Cell harvesting:

Sedimentation (Sdm,1):

Efficiency equations:

$$\eta_{sdm1} = \frac{M_{8,\mathrm{B}}}{M_{4,B}} \quad (3.1)$$

$$\eta_{sdm1} = \frac{M_{7,W}}{M_{4,W}} \quad (3.2)$$

Concentration factor ($\mathrm{CF_{sdm1}}$): (volume concentration factor)

$$\mathrm{CF_{sdm1}} = \frac{\left[\sum_{k \in \boldsymbol{K}_{\boldsymbol{j}}} \left(\frac{M_{4,k}}{\rho_k}\right)\right]}{\left[\sum_{k \in \boldsymbol{K}_{\boldsymbol{j}}} \left(\frac{M_{8,k}}{\rho_k}\right)\right]} \quad (3.3)$$

$$2 \leq \mathrm{CF}_{\mathrm{sdm1}} \leq 15$$

Area of sedimentation tank:

$$Qc_{sdm1} = \frac{\left[\sum_{k\in \boldsymbol{K_j}} \left(\frac{M_{4,k}}{\rho_k}\right)\right]}{SOR_{sdm1}} \quad (3.4)$$

SOR_{sdm} is an evaluated parameter:

$$\mathrm{SOR}_{\mathrm{sdm1}} = \eta_{\mathrm{sdm1}}\, \mathrm{U}_{\mathrm{g,Floc1}} \quad (3.5)$$

Centrifugation (Cnt,1):

Efficiency equations:

$$\eta_{cnt1} = \frac{M_{10,\mathrm{B}}}{M_{5,B}} \quad (4.1)$$

$$\eta_{cnt1} = \frac{M_{9,W}}{M_{5,W}} \quad (4.2)$$

Concentration factor ($\mathrm{CF}_{\mathrm{cnt1}}$):

$$\mathrm{CF}_{\mathrm{cnt1}} = \frac{\left[\sum_{k\in \boldsymbol{K_j}} \left(\frac{M_{5,k}}{\rho_k}\right)\right]}{\left[\sum_{k\in \boldsymbol{K_j}} \left(\frac{M_{10,k}}{\rho_k}\right)\right]} \quad (4.3)$$

$$2 \leq \mathrm{CF}_{\mathrm{cnt1}} \leq 20$$

Sigma factor equation:

$$Qc_{cnt1} U_{g,Floc1} = \left[\sum_{k\in \boldsymbol{K_j}} \left(\frac{M_{5,k}}{\rho_k}\right)\right] \quad (4.4)$$

Power required:

$$PW_{cnt1} = Wsp_{cnt1} \left[\sum_{k\in \boldsymbol{K_j}} \left(\frac{M_{5,k}}{\rho_k}\right)\right] \quad (4.5)$$

Power dissipation to heat is about 40%, hence cooling utility is required:

$$Mcw_{cnt1} Cp_W (Tcw_{out} - Tcw_{in}) = (0.4 PW_{cnt1}) \quad (4.6)$$

Filtration (Ftt,1):

Retention factor equations:

$$\xi_{k,ftt1} = \frac{M_{12,k}}{M_{6,k}} \;;\; \forall k \in \boldsymbol{K^{BD}} \quad (5.1)$$

Concentration factor ($\mathrm{CF}_{\mathrm{ftt1}}$):

$$\mathrm{CF}_{\mathrm{ftt1}} = \frac{\left[\sum_{k\in \boldsymbol{K_j}} \left(\frac{M_{6,k}}{\rho_k}\right)\right]}{\left[\sum_{k\in \boldsymbol{K_j}} \left(\frac{M_{12,k}}{\rho_k}\right)\right]} \quad (5.2)$$

$$2 \leq \mathrm{CF}_{\mathrm{ftt1}} \leq 30$$

Flux balance:

$$\zeta_{ftt1} Qc_{ftt1} = \left[\sum_{k \in \boldsymbol{K}_j} \left(\frac{M_{6,k}}{\rho_k}\right)\right]\left(1 - \frac{1}{CF_{ftt1}}\right) \quad (5.3)$$

Power required:

$$PW_{ftt1} = Wsp_{ftt1} Qc_{ftt1} \quad (5.4)$$

Cell disruption technologies:

Enzyme lysis (Ely):

Component release equation:

$$\mathrm{M}_{17,\mathrm{k}} = \Upsilon_{\mathrm{ely}} \mathrm{Frc}_{\mathrm{k}} \mathrm{M}_{14,\mathrm{B}};\ \forall \mathrm{k} \in \boldsymbol{K}^{\boldsymbol{C}} \quad (6.1)$$

Residual component (RS) after release:

$$M_{17,RS} = M_{14,B} - \sum_{k \in \boldsymbol{K}^{\boldsymbol{C}}} M_{17,k} \quad (6.2)$$

Enzyme added:

$$M_{16,Enz} = \phi_{add,\mathrm{Enz}} M_{14,B} \quad (6.3)$$

Enzyme cost:

$$Cpur_{Enz} = \pi_{Enz} M_{16,Enz} \quad (6.4)$$

Costing variables:

$$Qc_{ely} = \left[\sum_{k \in \boldsymbol{K}_j} \left(\frac{M_{17,k}}{\rho_k}\right)\right] \quad (6.5)$$

Power required:

$$PW_{ely} = Wsp_{ely} Qc_{ely} \quad (6.6)$$

Bead mill (Bml):

Component release equation:

$$M_{18,k} = \Upsilon_{bm} Frc_k M_{15,k};\ \forall \mathrm{k} \in \boldsymbol{K}^{\boldsymbol{C}} \quad (7.1)$$

Residual component (RS) after release:

$$M_{18,RS} = M_{15,B} - \sum_{k \in \boldsymbol{K}^{\boldsymbol{C}}} M_{18,kC} \quad (7.2)$$

Volume (costing variable) of bead mill technology:

$$Qc_{bml} == \theta_{bm} \left[\sum_{k \in \boldsymbol{K}_j} \left(\frac{M_{18,k}}{\rho_k}\right)\right] \quad (7.3)$$

Power required:

$$PW_{bml} = 13.33\ y_{bml} + 167 Qc_{bml} \quad (7.4)$$

Almost, 60 percent of power dissipates to heat, hence cooling is required.

Cooling water required:

$$Mcw_{bml} Cp_W (Tcw_{out} - Tcw_{in}) = (0.6 PW_{bml}) \quad (7.5)$$

Phase isolation technologies:

Microfiltration (mbr-mf):

Retention factor equations:

$$\xi_{k,mf} = \frac{M_{23,k}}{M_{20,k}} \; ; \; \forall k \in \; \boldsymbol{K^{JP}} \tag{8.1}$$

Concentration factor ($\mathrm{CF_{mf}}$):

$$\mathrm{CF_{mf}} = \frac{\left[\sum_{k \in \boldsymbol{K_j}} \left(\frac{M_{20,k}}{\rho_k}\right)\right]}{\left[\sum_{k \in \boldsymbol{K_j}} \left(\frac{M_{23,k}}{\rho_k}\right)\right]} \tag{8.2}$$

$$1.01 \leq \mathrm{CF_{mf}} \leq 35$$

Flux balance:

$$\zeta_{mf} Qc_{mf} = \left[\sum_{k \in \boldsymbol{K_j}} \left(\frac{M_{20,k}}{\rho_k}\right)\right]\left(1 - \frac{1}{CF_{mf}}\right) \tag{8.3}$$

Power required:

$$PW_{mf} = Wsp_{mf} Qc_{mf} \tag{8.4}$$

Flocculation-2 (Flc,2):

Flocculent added:

$$M_{25,Flcnt2} = \phi_{add,\mathrm{Flcnt2}} \left[\sum_{k \in \boldsymbol{K_j}} \left(\frac{M_{21,k}}{\rho_k}\right)\right] \tag{9.1}$$

Flocculent cost:

$$Cpur_{Flcnt2} = \pi_{Flcnt1} M_{25,Flcnt2} \tag{9.2}$$

Costing variable (volume) of flc2:

$$Qc_{flc2} = \theta_{flc2} \left[\sum_{k \in \boldsymbol{K_j}} \left(\frac{M_{21,k}}{\rho_k}\right)\right] \tag{9.3}$$

Power required:

$$PW_{flc2} = Wsp_{flc2} \left[\sum_{k \in \boldsymbol{K_j}} \left(\frac{M_{21,k}}{\rho_k}\right)\right] \tag{9.4}$$

Sedimentation (Sdm,2):

Efficiency equations:

$$\eta_{sdm2} = \frac{\sum_{k \in K^{H1}} M_{31,k}}{\sum_{k \in K^{H1}} M_{27,k}} \tag{10.1}$$

$$\eta_{sdm2} = \frac{\sum_{k \in K^{L1}} M_{30,k}}{\sum_{k \in K^{L1}} M_{27,k}} \tag{10.2}$$

Concentration factor ($\mathrm{CF_{sdm2}}$):

$$\mathrm{CF_{sdm2}} = \frac{\left[\sum_{k\in \boldsymbol{K_j}}\left(\frac{M_{27,k}}{\rho_k}\right)\right]}{\left[\sum_{k\in \boldsymbol{K_j}}\left(\frac{M_{31,k}}{\rho_k}\right)\right]} \tag{10.3}$$

$$2 \leq \mathrm{CF_{sdm2}} \leq 15$$

Costing variable:

$$Qc_{sdm2} = \frac{\left[\sum_{k\in \boldsymbol{K_j}}\left(\frac{M_{27,k}}{\rho_k}\right)\right]}{SOR_{sdm2}} \tag{10.4}$$

Centrifugation (cnt2):

Efficiency equations:

$$\eta_{cnt2} = \frac{\sum_{k\in K^{H1}} M_{33,k}}{\sum_{k\in K^{H1}} M_{28,k}} \tag{11.1}$$

$$\eta_{cnt2} = \frac{\sum_{k\in K^{L1}} M_{32,k}}{\sum_{k\in K^{L1}} M_{28,k}} \tag{11.2}$$

Concentration factor ($\mathrm{CF_{cnt2}}$):

$$\mathrm{CF_{cnt2}} = \frac{\left[\sum_{k\in \boldsymbol{K_j}}\left(\frac{M_{28,k}}{\rho_k}\right)\right]}{\left[\sum_{k\in \boldsymbol{K_j}}\left(\frac{M_{33,k}}{\rho_k}\right)\right]} \tag{11.3}$$

$$2 \leq \mathrm{CF_{cnt2}} \leq 25$$

Design equations:

Sigma factor equation:

$$Qc_{cnt2} U_{g,Floc2} = \left[\sum_{k\in \boldsymbol{K_j}}\left(\frac{M_{28,k}}{\rho_k}\right)\right] \tag{11.4}$$

Power required:

$$PW_{cnt2} = Wsp_{cnt2}\left[\sum_{k\in \boldsymbol{K_j}}\left(\frac{M_{28,k}}{\rho_k}\right)\right] \tag{11.5}$$

Power dissipation to heat is about 40%, hence cooling utility is required:

$$Mcw_{cnt2} Cp_W (Tcw_{out} - Tcw_{in}) = (0.4 PW_{cnt2}) \tag{11.6}$$

Filtration (Ftt,2):

Retention factor equations:

$$\xi_{k,ftt2} = \frac{M_{35,k}}{M_{29,k}} \;;\; \forall k \in \boldsymbol{K^{JP}} \tag{12.1}$$

Concentration factor ($\mathrm{CF_{ftt2}}$):

$$\mathrm{CF_{ftt2}} = \frac{\left[\sum_{k\in \boldsymbol{K_j}}\left(\frac{M_{29,k}}{\rho_k}\right)\right]}{\left[\sum_{k\in \boldsymbol{K_j}}\left(\frac{M_{35,k}}{\rho_k}\right)\right]} \tag{12.2}$$

$2 \leq \mathrm{CF}_{\mathrm{ftt2}} \leq 30$

Flux balance:

$$\zeta_{ftt2} Qc_{ftt2} = \left[\sum_{k \in \boldsymbol{K_j}} \left(\frac{M_{29,k}}{\rho_k}\right)\right] \left(1 - \frac{1}{CF_{ftt2}}\right) \quad (12.3)$$

Power required:

$$PW_{ftt2} = Wsp_{ftt2} Qc_{ftt2} \quad (12.4)$$

Stage – II (Product concentration) technologies

Distillation1 (Dst,1):

Molar flow rates in dst,1:

$$F_{j,k} = \frac{M_{j,k}}{MW_k} \; ; \; \forall \; j \in \boldsymbol{J^{dst1}}, k \in \boldsymbol{K^{dst1}} \quad (13.1)$$

Component balance in dst,1:

$$\sum_{j \in \boldsymbol{Jin_i}} F_{j,k} = \sum_{j \in \boldsymbol{Jout_i}} F_{j,k} \; ; \; \forall \; j \in \boldsymbol{J^{dst1}}, k \in \boldsymbol{K^{dst1}} \quad (13.2)$$

Mole fractions in dst,1:

$$Xm_{j,k} = \frac{F_{j,k}}{\sum_{k \in \boldsymbol{K^{dst1}}} F_{j,k}} ; \; \forall \; j \in \boldsymbol{J^{dst1}} \quad (13.3)$$

Written as: $Xm_{j,k} \sum_{k \in \boldsymbol{K^{dst1}}} F_{j,k} = F_{j,k}$

Constraints on recovery:

Heavier than heavy key (HK - water) components are not in the distillate:

$$Xm_{42,k} \, when(\alpha_k < \alpha_{HK}) = 0 \; ; \; \forall k \in \boldsymbol{K^{dst1}} \quad (13.4)$$

Lighter than light key (LK - product) components are not in the bottoms:

$$Xm_{43,k} \, when(\alpha_k > \alpha_{LK}) = 0 \; ; \; \forall k \in \boldsymbol{K^{dst1}} \quad (13.5)$$

Distillate recovery constraints:

$$Xm_{42,Prd} = 0.9 \quad (13.6)$$

$$Xm_{42,Slb} = 0.02 \quad (13.7)$$

$$Xm_{42,W} = 0.08 \quad (13.8)$$

Design calculations:

The <u>minimum number of stages</u> can be calculated using Fenske's equation.

$$N1_{min} \, log \, \alpha_{Prd} = log \left[\left(\frac{Xm_{42,Prd}}{Xm_{42,W}}\right)\left(\frac{Xm_{43,W}}{Xm_{43,Prd}}\right)\right] \quad (13.9)$$

Written as: $N1_{min} \log \alpha_{Prd} = \log(Xm_{42,Prd}) + \log(Xm_{43,W}) - \log(Xm_{42,W}) - \log(Xm_{43,Prd})$

Underwood's variable ($\mathrm{U_{v1}}$) is evaluated by using;

$$(1 - q) = \sum_{k \in \boldsymbol{K^{dst1}}} \frac{\alpha_k Xm_{38,k}}{\alpha_k - \mathrm{U_{v1}}} \quad (13.10)$$

Assume feed to be saturated liquid (q=1), L.H.S is zero

$$\sum_{k\in \boldsymbol{K}^{dst1}} \frac{\alpha_k Xm_{38,k}}{\alpha_k - \mathrm{U_{v1}}} = 0$$

Written as: $\sum_{k\in \boldsymbol{K}^{dst1}} \left[\alpha_k Xm_{38,k} \prod_{k\in \boldsymbol{KK}^{dst1}} (\alpha_k - \mathrm{U_{v1}})\right] = 0\,;\ (when\ \boldsymbol{K}^{dst1} \neq \boldsymbol{KK}^{dst1})$

Minimum reflux ratio ($R1_{min}$)

$$R1_{min} = \sum_{k\in \boldsymbol{K}^{dst1}} \frac{\alpha_k Xm_{42,k}}{\alpha_k - \mathrm{U_{v1}}} - 1 \qquad (13.11)$$

Written as: $(1 + R1_{min}) \prod_{k\in \boldsymbol{K}^{dst1}} (\alpha_k - \mathrm{U_{v1}}) = \sum_{k\in \boldsymbol{K}^{dst1}} \left[\alpha_k Xm_{42,k} \prod_{k\in \boldsymbol{KK}^{dst1}} (\alpha_k - \mathrm{U_{v1}})\right]\,;\ (when\ \boldsymbol{K}^{dst1} \neq \boldsymbol{KK}^{dst1})$

Reflux ratio ($R1$)

$R1$ – reflux ratio, usually 10-50% greater than R_{min}.

$$R1 = 1.3R1_{min} \qquad (13.12)$$

Number of theoretical stages ($N1$) (Gilliland, 1940)

$$\left(\frac{N1 - N1_{min}}{N1+1}\right) = 0.75 - 0.75\left(\frac{R1 - R1_{min}}{R1+1}\right)^{0.5668} \qquad (13.13)$$

(Requires either $N1_{min}$ or $R1_{min}$ to be already specified)

Simplified equation suggested by Towler and Sinnot, 2012

$$0.6N1 = N1_{min} \qquad (13.14)$$

Number of actual stages ($N1_{act}$)

Number of actual stages is calculated by dividing the number of theoretical stages by the stage efficiency (η_{stage} - approximately 80%).

$$N1_{act} = \frac{N1}{\eta_{stage}} \qquad (13.15)$$

Height of column (H_{dst1})

The actual number of stages times the stage height (H_{stage}) yields the height of the column.

$$H_{dst1} = H_{stage}\, N1_{act} \qquad (13.16)$$

Liquid (Liq_{dst1}) and vapor (Vap_{dst1}) flowrates within the column:

$$Liq_{dst1} = R1 \sum_{k\in \boldsymbol{K}^{dst1}} M_{42,k} \qquad (13.17)$$

$$Vap_{dst1} = Liq_{dst1} + \sum_{k\in \boldsymbol{K}^{dst1}} M_{42,k} \qquad (13.18)$$

Column diameter (D_{dst1})

The column diameter is calculated using vapor flowrate in the column.

$$D_{dst1} = \sqrt{\frac{4Vap_{dst1}}{\pi u_{vap}}} \qquad (13.19)$$

u_{vap} – vapor linear velocity (m/h) – 10800 m/h

Written as: $\pi u_{vap} D_{dst1}{}^2 = 4Vap_{dst1}$

Volume (costing variable) of distillation column:

$$Qc_{dst1} = \frac{\pi}{4} D_{dst1}{}^2 H_{dst1} \quad (13.20)$$

Utility requirements:

Initial heating (QS_{dst1}) of the feed to reach saturation temperature:

$$QS_{dst1} = \sum_{k \in \boldsymbol{K^{dst1}}} M_{38,k}\, Cp_k\, (T_{sat} - T_{amb}) \quad (13.21)$$

Heat duty in distillation-1:

$$QH_{dst1} = (1 + R1) \sum_{k \in \boldsymbol{K^{dst1}}} F_{42,k} MW_k\, \lambda^{vap}{}_k \quad (13.22)$$

Cooling in distillation-1:

$$QC_{dst1} = R1 \sum_{k \in \boldsymbol{K^{dst1}}} F_{42,k} MW_k\, \lambda^{vap}{}_k \quad (13.23)$$

Steam required:

$$Mstm_{dst1} \lambda_{stm} = QS_{dst1} + QH_{dst1} \quad (13.24)$$

Cooling water required:

$$Mcw_{dst1} Cp_W (Tcw_{out} - Tcw_{in}) = QC_{dst1} \quad (13.25)$$

Equations for variable bounds:

$$N1_{min} \geq y_{dst1} \quad (13.26)$$

$$R1_{min} \geq 1.01 y_{dst1} \quad (13.27)$$

ATPE (Atpe):

Solubility equations for solvents (Poly (top) – Salt (bottom)):

$$M_{46,\mathrm{Poly}} = \psi_{Poly-BP} M_{46,\mathrm{Salt}} \quad (14.1)$$

$$M_{45,\mathrm{Salt}} = \psi_{Salt-TP} M_{45,\mathrm{Poly}} \quad (14.2)$$

Extraction factor ($EFatpe_{,k}$) for solutes ($\boldsymbol{Kp^{atpe}}$):

$$EFatpe_k = \frac{\kappa PT_k\, M_{44,Poly}}{M_{44,Salt}};\ |\forall\, k \in \boldsymbol{Kp^{atpe}} \quad (14.3)$$

Written as: $EFatpe_k M_{44,Salt} = \kappa PT_k\, M_{44,Poly}$

Design equations:

Equation for number of stages (N_{atpe}) in terms of the solutes not recovered in top phase (TP):

$$\frac{(M_{39,k} - M_{45,k})}{M_{39,k}} = \left(\frac{EFatpe_k - 1}{EFatpe_k{}^{(N_{atpe}+1)} - 1} \right);\ \ ,|\forall\, k\ \in \boldsymbol{Kp^{atpe}} \quad (14.4)$$

Written as: $\left(M_{39,k} - M_{45,k}\right)\left(EFatpe_k{}^{(N_{atpe}+1)} - 1\right) = M_{39,k}\left(EFatpe_k - 1\right)$

Costing variable:

$$Qc_{atpe} = \left[\sum_{k\in \boldsymbol{K_j}} \left(\frac{M_{39,k}}{\rho_k}\right)\right] + \left[\sum_{k\in \boldsymbol{K_j}} \left(\frac{M_{44,k}}{\rho_k}\right)\right] \quad (14.5)$$

Power required:

$$PW_{atpe} = Wsp_{atpe} Qc_{atpe} \quad (14.6)$$

Purchase costs of salt and polymer:

$$Cpur_{Salt} = \pi_{Salt} M_{49,Salt} \quad (14.7)$$

$$Cpur_{Poly} = \pi_{Poly} M_{50,Poly} \quad (14.8)$$

Equations for variable bounds:

$$N_{atpe} \geq y_{atpe} \quad (14.9)$$

$$EFatpe_{Prd} \geq 1.01 y_{atpe} \quad (14.10)$$

<u>Membrane-Ultrafiltration (mbr-uf):</u>

Retention factor equations:

$$\xi_{k,uf} = \frac{M_{47,k}}{M_{45,k}} \; ; \; \forall k \in \boldsymbol{K_j} \quad (15.1)$$

Concentration factor ($\mathrm{CF_{uf}}$):

$$\mathrm{CF_{uf}} = \frac{\left[\sum_{k\in \boldsymbol{K_j}} \left(\frac{M_{45,k}}{\rho_k}\right)\right]}{\left[\sum_{k\in \boldsymbol{K_j}} \left(\frac{M_{47,k}}{\rho_k}\right)\right]} \quad (15.2)$$

$$1.01 \leq \mathrm{CF_{uf}} \leq 35$$

Flux balance:

$$\zeta_{uf} Qc_{uf} = \left[\sum_{k\in \boldsymbol{K_j}} \left(\frac{M_{45,k}}{\rho_k}\right)\right]\left(1 - \frac{1}{CF_{uf}}\right) \quad (15.3)$$

Power required:

$$PW_{uf} = Wsp_{uf} Qc_{uf} \quad (15.4)$$

<u>Extraction (Ext):</u>

Solubility equations for solvent and water

$$M_{52,W} = \psi_{W-Sol} M_{52,Sol} \quad (16.1)$$

$$M_{53,Sol} = \psi_{Sol-W} M_{53,W} \quad (16.2)$$

Extraction factor ($EFext_k$) for solutes ($\boldsymbol{Kp^{ex}}$):

$$EFext_k = \frac{\kappa P_k \, M_{51,Solv}}{M_{40,W}} ; |\forall \; k \in \boldsymbol{Kp^{ex}} \quad (16.3)$$

Written as: $EFext_k M_{40,W} = \kappa P_k \; M_{51,Solv}$

Design equations:

Equation for number of stages (N_{atpe}) in terms of the solutes ($\boldsymbol{Kp^{ex}}$) remaining in raffinate phase:

$$\frac{(M_{40,k}-M_{52,k})}{M_{40,k}} = \left(\frac{EFext_k - 1}{EFext_k^{(N_{ext}+1)} - 1}\right); \;\; |\forall\, k \;\in \boldsymbol{Kp^{ex}} \tag{16.4}$$

Written as:

$$(M_{40,k} - M_{52,k})(EFext_k^{(N_{ext}+1)} - 1) = M_{40,k}(EFext_k - 1)$$

Desired product recovery constraint:

$$M_{52,Prd} = 0.9M_{40,Prd} \tag{16.5}$$

Capacity of the extraction (Qc_{ext}):

$$Qc_{ext} = \left[\sum_{k\in \boldsymbol{K_j}}\left(\frac{M_{40,k}}{\rho_k}\right)\right] + \left[\sum_{k\in \boldsymbol{K_j}}\left(\frac{M_{51,k}}{\rho_k}\right)\right] \tag{16.6}$$

Power required:

$$PW_{ext} = Wsp_{ext}Qc_{ext} \tag{16.7}$$

Solvent cost:

$$Cpur_{Solv} = \pi_{Solv}M_{56,Solv} \tag{16.8}$$

Equations for variable bounds:

$$N_{ext} \geq y_{ext} \tag{16.9}$$

<u>Distillation-2 (Dst,2):</u>

Molar flow rates in dst-2:

$$F_{j,k} = \frac{M_{j,k}}{MW_k}\; ; \; \forall\, j \in \boldsymbol{J^{dst2}}, k \in \boldsymbol{K^{dst2}} \tag{17.1}$$

Component balance in dst,2:

$$\sum_{j\in \boldsymbol{Jin_i}} F_{j,k} = \sum_{j\in \boldsymbol{Jout_i}} F_{j,k}\; ; \; \forall\, j \in \boldsymbol{J^{dst2}}, k \in \boldsymbol{K^{dst2}} \tag{17.2}$$

Mole fractions in dst,2:

$$Xm_{j,k} = \frac{F_{j,k}}{\sum_{k\in \boldsymbol{K^{dst2}}} F_{j,k}}; \; \forall\, j \in \boldsymbol{J^{dst2}} \tag{17.3}$$

Constraints on recovery:

Heavier than heavy key (HK - product) components are not in the distillate:

$$Xm_{54,k}\; when\; (\alpha_k < \alpha_{HK}) = 0\; ; \; \forall k \in \boldsymbol{K^{dst2}} \tag{17.4}$$

Lighter than light key (LK - solvent) components are not in the bottoms:

$$Xm_{55,k}\; when\; (\alpha_k > \alpha_{LK}) = 0\; ; \; \forall k \in \boldsymbol{K^{dst2}} \tag{17.5}$$

Distillate recovery constraints:

$$Xm_{54,Prd} = 0.08 \quad (17.6)$$

$$Xm_{54,Solv} = 0.92 \quad (17.7)$$

Design calculations:

The minimum number of stages can be calculated using Fenske's equation.

$$N2_{min} \log \alpha_{Solv} = \log \left[\left(\frac{Xm_{54,Solv}}{Xm_{54,Prd}} \right) \left(\frac{Xm_{55,Prd}}{Xm_{55,Solv}} \right) \right] \quad (17.8)$$

Underwood's variable (U_{v2}) is decided by using the expression;

$$(1 - q) = \sum_{k \in \boldsymbol{K}^{dst2}} \frac{\alpha_k Xm_{52,k}}{\alpha_k - U_{v2}} \quad (17.9)$$

Assume feed to be saturated liquid (q=1), L.H.S is zero

$$\sum_{k \in \boldsymbol{K}^{dst2}} \frac{\alpha_k Xm_{52,k}}{\alpha_k - U_{v2}} = 0$$

Minimum reflux ratio ($R2_{min}$)

$$R2_{min} = \sum_{k \in \boldsymbol{K}^{dst2}} \frac{\alpha_k Xm_{54,k}}{\alpha_k - U_{v2}} - 1 \quad (17.10)$$

Reflux ratio ($R2$)

$R2$ – reflux ratio, usually 10-50% greater than R_{min}.

$$R2 = 1.3 R2_{min} \qquad \text{(assumed)} \quad (17.11)$$

Number of stages equation:

Simplified equation suggested by Towler and Sinnot, 2016

$$0.6 N2 = N2_{min} \quad (17.12)$$

Number of actual stages ($N2_{act}$) is calculated by dividing the number of theoretical stages by the stage efficiency (η_{stage} - approximately 80%).

$$N2_{act} = \frac{N2}{\eta_{stage}} \quad (17.13)$$

Height of column (H_{dst2})

The actual number of stages times the stage height (H_{stage}) yields the height of the column.

$$H_{dst2} = H_{stage} \, N2_{act} \quad (17.14)$$

Liquid (Liq_{dst2}) and vapor (Vap_{dst2}) flowrates within the column:

$$Liq_{dst2} = R2 \sum_{k \in \boldsymbol{K}^{dst2}} M_{54,k} \quad (17.15)$$

$$Vap_{dst2} = Liq_{dst2} + \sum_{k \in \boldsymbol{K}^{dst2}} M_{54,k} \quad (17.16)$$

Column diameter (D_{dst2})

The column diameter is calculated using vapor flowrate in the column.

$$D_{dst2} = \sqrt{\frac{4Vap_{dst2}}{\pi u_{vap}}} \tag{17.17}$$

u_{vap} – vapor linear velocity (m/h) – 10800 m/h

Costing variable (volume) of distillation column:

$$Qc_{dst2} = \frac{\pi}{4} {D_{dst2}}^2 H_{dst2} \tag{17.18}$$

Utility requirements:

Initial heating (QS_{dst2}) of the feed to reach saturation temperature:

$$QS_{dst2} = \sum_{k\in \boldsymbol{K}^{dst2}} M_{52,k}\, Cp_k\, (T_{sat} - T_{amb}) \tag{17.19}$$

Heat duty in distillation-2:

$$QH_{dst2} = (1 + R2) \sum_{k\in \boldsymbol{K}^{dst2}} F_{54,k} MW_k\, {\lambda^{vap}}_k \tag{17.20}$$

Cooling in distillation-2:

$$QC_{dst2} = R2 \sum_{k\in \boldsymbol{K}^{dst2}} F_{54,k} MW_k\, {\lambda^{vap}}_k \tag{17.21}$$

Steam required:

$$Mstm_{dst2}\lambda_{stm} = QS_{dst2} + QH_{dst2} \tag{17.22}$$

Cooling water required:

$$Mcw_{dst2}Cp_W(Tcw_{out} - Tcw_{in}) = QC_{dst2} \tag{17.23}$$

Equations for variable bounds:

$$N2_{min} \geq y_{dst2} \tag{17.24}$$

$$R2_{min} \geq 1.01 y_{dst2} \tag{17.25}$$

Stage III (Product purification and refinement) technologies:

Chromatography (Chr)

Parameters for *chr* technology:

θ_{chr} – space time or residence time

κc_{chr} – capacity factor

w_{chr} – width of chromatogram

$HETP_{chr}$ – height equivalent to theoretical plates

$R_{LD,chr}$ – ratio of length to diameter

Parameter calculations:

Retention time: $\tau R_{chr} = (1 + \kappa c_{chr})\theta_{chr}$

Number of plates: $Np_{chr} = 16\left(\frac{\tau R_{chr}}{w_{chr}}\right)^2$

Length of column: $L_{chr} = HETP_{chr} Np_{chr}$

Diameter of column: $D_{chr} = L_{chr}/R_{LD,chr}$

Volume of column: $V1_{chr} = \frac{\pi}{4} {D_{chr}}^2 L_{chr}$

<u>Model equations</u>

Solvents and other components removed by chromatography

$$\sum_{k\in \boldsymbol{K}^{OTP}} M_{62,k} = \kappa c_{chr} \sum_{k\in \boldsymbol{K}^{OTP}} M_{59,k} \tag{18.1}$$

Product not retained by chromatography column

$$M_{61,Prd} = M_{59,Prd} \tag{18.2}$$

Number of columns required

$$Ncol_{chr} V1_{chr} = \theta_{chr} \sum_{k\in \boldsymbol{K_j}} \frac{M_{59,k}}{\rho_k} \tag{18.3}$$

Total volume (costing variable) for chromatography

$$Qc_{chr} = Ncol_{chr} V1_{chr} \tag{18.4}$$

<u>Pervaporation (Pvp):</u>

Retention factor equations:

$$\xi_{k,pvp} = \frac{M_{68,k}}{M_{65,k}} \ ; \ \forall k \in \boldsymbol{K_j} \tag{19.1}$$

Concentration factor ($\mathrm{CF_{pvp}}$)

$$\mathrm{CF_{pvp}} = \frac{\left[\sum_{k\in \boldsymbol{K_j}} \left(\frac{M_{65,k}}{\rho_k}\right)\right]}{\left[\sum_{k\in \boldsymbol{K_j}} \left(\frac{M_{68,k}}{\rho_k}\right)\right]} \tag{19.2}$$

$$1.01 \le \mathrm{CF_{pvp}} \le 35$$

Flux balance:

$$\zeta_{pvp} Qc_{pvp} = \left[\sum_{k\in \boldsymbol{K_j}} \left(\frac{M_{65,k}}{\rho_k}\right)\right] \left(1 - \frac{1}{CF_{pvp}}\right) \tag{19.3}$$

Power required:

$$PW_{pvp} = Wsp_{pvp} Qc_{pvp} \tag{19.4}$$

Heat required for vaporization of permeate stream:

$$Mstm_{pvp} \lambda_{stm} = \sum_{k\in \boldsymbol{K_j}} M_{67,k} \, {\lambda^{vap}}_k \tag{19.5}$$

<u>Bleaching (Blc):</u>

Impurity removal efficiency

$$\sum_{k\in \boldsymbol{K}^{OTP}} M_{71,k} = \eta_{blc} \sum_{k\in \boldsymbol{K}^{OTP}} M_{70,k} \tag{20.1}$$

Product not retained by adsorbent in bleaching

$$M_{72,Prd} = M_{70,Prd} \tag{20.2}$$

Volume (costing variable) of bleaching

$$Qc_{blc} = \theta_{blc} \sum_{k\in \boldsymbol{K_j}} \frac{M_{70,k}}{\rho_k} \tag{20.3}$$

Power required in bleaching

$$PW_{blc} = Wsp_{blc} Qc_{blc} \tag{20.4}$$

Product final conditions:

Product purity:

$$M_{72,Prd} = Pur \sum_{k\in \boldsymbol{K_j}} M_{72,k} \tag{21.1}$$

Final product amount:

$$\sum_{k\in \boldsymbol{K_j}} M_{72,k} = Prd_F \tag{21.2}$$

Equations for evaluating stage-wise categorical costs:

Fixed capital cost in each stage (annualized)

$$CCAC_{Nstg} = 1.66\ \varsigma_{RF} BMC_{mult} \sum_{i\in istg\{1,2,3\}} Ce_i \tag{22.1}$$

Materials cost in stages:

$$CCRM_{s1} = [\tau_{ann}(Cpur_{Flcnt1} + Cpur_{Flcnt2} + Cpur_{Enz})] \tag{22.2a}$$

$$CCRM_{s2} = [\tau_{ann}(Cpur_{Salt} + Cpur_{Poly} + Cpur_{Solv})] \tag{22.2b}$$

$$CCRM_{s3} = 0 \tag{22.2c}$$

Consumable costs in each stage:

$$CCRM_{s1} = Cons_{ftt1} + Cons_{ftt2} + Cons_{mf} \tag{22.3a}$$

$$CCRM_{s2} = Cons_{uf} \tag{22.3b}$$

$$CCRM_{s3} = Cons_{pvp} + Cons_{blc} \tag{22.3c}$$

Labor costs in each stage:

$$CCLB_{Nstg} = \tau_{ann}\ C_{Lab} \sum_{i\in istg\{1,2,3\}} Nlb_i \tag{22.4}$$

Utility costs in each stage:

$$CCUT_{Nstg} = \tau_{ann} \begin{bmatrix} \left(C_{elec} \sum_{i\in istg\{1,2,3\}} PW_i\right) + \left(C_{cwt} \sum_{i\in istg\{1,2,3\}} Mcwt_i\right) \\ +\left(C_{stm} \sum_{i\in istg\{1,2,3\}} Mstm_i\right) \end{bmatrix} \tag{22.5}$$

Total cost in each stage:

$$CCTC_{Nstg} = CCAC_{Nstg} + CCRM_{Nstg} + CCCS_{Nstg} + 2.78\ CCLB_{Nstg} + CCUT_{Nstg} \tag{22.6}$$

Other costs in each stage:

$$CCOT_{Nstg} = CCTC_{Nstg} - \{CCAC_{Nstg} + CCRM_{Nstg} + CCCS_{Nstg} + CCLB_{Nstg} + CCUT_{Nstg}\} \tag{22.7}$$

Total costs in different categories:

$$CCTFC = \tau_{ann}\, C_{Feed} \tag{23.1}$$

$$CCTAC = \sum_{n \in Nstg} CCAC_n \tag{23.2}$$

$$CCTRM = \sum_{n \in Nstg} CCRM_n \tag{23.3}$$

$$CCTCS = \sum_{n \in Nstg} CCCS_n \tag{23.4}$$

$$CCTLB = \sum_{n \in Nstg} CCLB_n \tag{23.5}$$

$$CCTUT = \sum_{n \in Nstg} CCUT_n \tag{23.6}$$

$$CCTOT = \sum_{n \in Nstg} CCOT_n \tag{23.7}$$

Total process cost:

$$CCTPC(\$/annum) = CCTFC + CCTAC + CCTRM + CCTCS + CCTLB + CCTUT + CCTOT \tag{24}$$

Objective function:

$$Obj = Min\, CCTPC \tag{25}$$

Overall process cost per unit product:

$$UPC(\$/kg) = \frac{CCTPC}{\tau_{ann} Prd_F} \tag{26}$$

C.3 Model parameters and input data for base case

Table C.3.1 Important input parameters and product specifications

Parameter	Nominal value	Units
Initial cell titer	5	g/L (kg/m^3)
Cell diameter	2	μm
Product content in cells	20	wt% of cell dry weight (CDW)
Desired production capacity	500	kg/h
Annual operation time	330	days/year
Final product purity	99	wt% purity

Table C.3.2 Cell dry weight composition:

Component	Mass composition (wt %)
Product	20%
Debris	45%
Soluble co-product	35%

Table C.3.3 Component density data:

Component	Density (kg/m3)
Water	1000
Biomass	1100
Product	850
Debris	1950
Soluble co-product	1100

C.3.4 Sedimentation tank (Sdm,#1,2):

Efficiency – 70%

Depth – 3m

C.3.5 Flocculation (Flc,#1,2):
Flocculent added – 0.04 kg/m3
Residence time – 0.5 hr
Floc diameter (#1) – 1E-5 m
Floc diameter (#2) – 5E-6 m
Flocculent cost – 5 $/kg

C.3.6 Centrifuge (Cnt,#1,2):

Parameter/Technology	Cnt1	Cnt2
Efficiency	80%	85%
Rotation speed	9000 rpm	12000 rpm

C.3.7 Filtration (Ftt,#1,2):
Flux - 0.2 $m^3m^{-2}h^{-1}$
Retention factors (Ftt,1): Biomass - 80%, Water - 20%
Retention factors (Ftt,2): product - 20%, Water - 20%, other solid components (debris)- 80%, soluble co-product and enzyme - 20%
Filter cost – 100 $/m^2
Replacement time – 2000 h

C.3.8 Bead mill (Bml):
Product release – 85% (k – 0.02, phi – 0.82)

C.3.9 Enzyme lysis (Ely):
Enzyme addition – 0.02 kg/kg biomass
Enzyme cost – 50 $/kg
Density enzyme – 1150 kg/m^3
Product release – 90%

C.3.10 Microfiltration (MF,1):
Flux – 0.0856 $m^3m^{-2}h^{-1}$
Retention factor: product, water and soluble co-product – 0.15, heavy solid (debris) and enzyme – 0.85
Membrane cost – 736 $/m^2
Replacement time – 2000 h

C.3.11 Distillation (Dst,1):
Relative volatility: product – 2.5, water – 1, soluble co-product – 1.05, heavy solid (debris) – 0.01, enzyme – 0.05
Heat of vaporization (KJ/kg): product – 2000, water – 2257, soluble co-product – 1875, heavy solid (debris) – 2257, enzyme – 2257
Feed quality, q_f = 1 (saturated liquid)
Vapor velocity – 3 m/s
Stage efficiency – 80%
Height of stage – 0.6 m
Reflux ratio multiplying factor – 1.3

C.3.12 ATPE (Atpe)
Partition coefficient in top phase: product – 5, water – 1, soluble co-product – 2, heavy solid (debris) – 0.001, enzyme – 0.001
Solubility of polymer in bottom phase – 0.005 (kg/kg)
Solubility of salt in top phase – 0.005 (kg/kg)
Polymer: Mol. Wt. – 450, Density – 1850 (kg/m^3), Cost – 2 $/kg
Salt: Mol. Wt. – 136, Density – 2338 (kg/m^3), Cost – 0.6 $/kg

C.3.13 Ultrafiltration (UF):
Flux – 0.0856 $m^3m^{-2}h^{-1}$
Retention factor: product, water and soluble co-product – 0.15, heavy solid (debris) and enzyme – 0.85
Membrane cost – 981 $/m^2

Replacement time – 2000 h

C.3.14 Extraction (Ext):
Partition coefficient in solvent phase: product – 1.2, soluble co-product – 0.3, heavy solid (debris) – 0.0001, enzyme – 0.0001
Solubility of solvent in water – 0.03 (kg/kg)
Solubility of water in solvent – 0.02 (kg/kg)
Solvent: Mol. Wt. – 78, Density – 810 (kg/m^3), Cost – 1.5 $/kg

C.3.15 Distillation (Dst,2): (some parameters are same as listed in C.3.12)
Relative volatility: solvent – 6, product – 1, water – 0.05, soluble co-product – 0.04, heavy solid (debris) – 0.01, enzyme – 0.05
Heat of vaporization (KJ/kg): solvent – 520, product – 2000, water – 2257, soluble co-product – 1875, heavy solid (debris) – 2257, enzyme – 2257

C.3.16 Chromatography (Chr):
Space time – 0.5 h
Column capacity – 95%
Width of chromatogram – 0.05 m
HETP – 0.0035
Ratio length to diameter – 0.14

C.3.17 Pervaporation (Pvp):
Flux – 0.055 $m^3m^{-2}h^{-1}$
Retention factor: product – 0.0002, water – 0.95, soluble co-product – 0.92, heavy solid (debris) and enzyme – 0.99, salt – 0.99, polymer – 0.99, solvent – 0.001
Membrane cost – 1000 $/$m^2$
Replacement time – 2000 h

C.3.18 Bleaching (Blc):
Bleaching efficiency – 99%
Cost of GAC (bleaching agent) – 4$/kg
Replacement time – 360 h (15 days)

C.3.20 Table for standard capacity, costs, scaling factors, labor requirements for technologies:

Unit operation (costing capacity)	Standard capacity (units)	Base costs (million $)	Scaling exponent (n)	Laborers required (#/h)	Power required (KW/h)
Flocculation (Volume)	2000 m^3	0.538	0.5	0.1	0.0002
Sedimentation (Area)	2500 m^2	1.128	0.57	0.1	0
Centrifuge#1 (Sigma factor)	60000 m^2	0.275	0.65	1	12.79
Filtration (Area)	80 m^2	0.039	0.55	0.5	0.1
Bead milling (Volume)	0.275 m^3	0.272	0.95	0.5	(calculated from eq. 8.4 in A.2.6)
Microfiltration (Area)	80 m^2	0.75	0.55	1	0.1
Centrifuge#2 (Sigma factor)	60000 m^2	0.66	0.65	1	19.2
Ultrafiltration (Area)	80 m^2	0.938	0.55	1	0.2
Distillation (Volume)	22.58 m^3	0.082	2.8	1	0

ATPE (Flowrate)	185 m^3/h	0.362	0.67	1	0.5
Extraction (Flowrate)	185 m^3/h	0.362	0.67	1	0.5
Chromatography (Volume)	0.633 m^3	0.775	0.67	1	0.33
Pervaporation (Area)	80 m^2	0.261	0.55	1	0.33
Bleaching (Volume)	0.27 m^3	0.1	0.67	1	0.33

C.4 Integer-cuts for alternate configurations

Integer cuts added successively to the optimization model for study case II are:

(1) Integer cut#1:

$$\begin{bmatrix} y_{cnt1} + y_{ely} + y_{flc2} + y_{cnt2} \\ +y_{dst1} + y_{byp3} + y_{byp4} \end{bmatrix} - \begin{bmatrix} y_{sdm1} + y_{ftt1} + y_{bml} + y_{mf} + y_{sdm2} + y_{ftt2} \\ + y_{byp1} + y_{atpe} + y_{uf} + y_{ext} + y_{dst2} + y_{byp2} \\ +y_{chr} + y_{pvp} \end{bmatrix} \leq (7) - 1 \quad (27)$$

(2) Integer cut#2:

$$\begin{bmatrix} y_{cnt1} + y_{ely} + y_{flc2} + y_{sdm2} \\ +y_{dst1} + y_{byp3} + y_{byp4} \end{bmatrix} - \begin{bmatrix} y_{sdm1} + y_{ftt1} + y_{bml} + y_{mf} + y_{cnt2} + y_{ftt2} \\ + y_{byp1} + y_{atpe} + y_{uf} + y_{ext} + y_{dst2} + y_{byp2} \\ +y_{chr} + y_{pvp} \end{bmatrix} \leq (7) - 1 \quad (28)$$

(3) Integer cut#3:

$$\begin{bmatrix} y_{cnt1} + y_{ely} + y_{flc2} + y_{ftt2} \\ +y_{dst1} + y_{byp3} + y_{byp4} \end{bmatrix} - \begin{bmatrix} y_{sdm1} + y_{ftt1} + y_{bml} + y_{mf} + y_{sdm2} + y_{cnt2} \\ + y_{byp1} + y_{atpe} + y_{uf} + y_{ext} + y_{dst2} + y_{byp2} \\ +y_{chr} + y_{pvp} \end{bmatrix} \leq (7) - 1 \quad (29)$$

C.5 Additional results from case study - 2

C.5.1 Stage-wise categorical cost contributions

Base case

The stage-wise categorical (annualized capital, materials, consumables, utilities, labor and others) cost contributions for the base case is shown in Figure C.4. In stage-II, the utility costs are very high (~94%) because of the energy required in the distillation column. The relative volatility and heat of vaporization of the components are the major parameters that affect the utility costs.

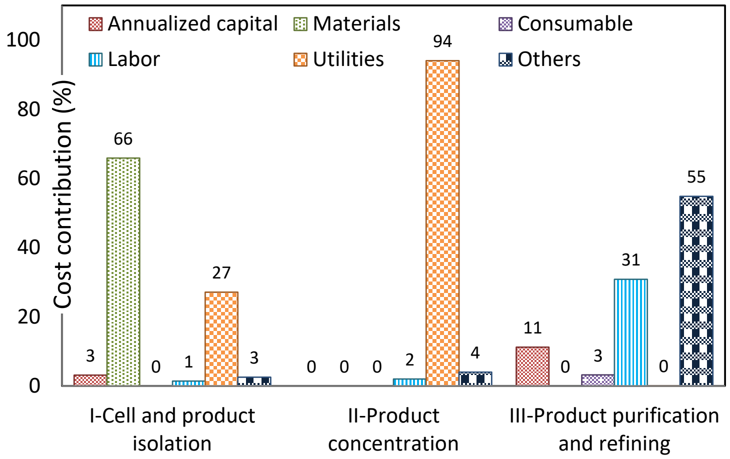

Figure C.4 Cost contribution by different categories (annualized capital, materials, consumables, labor, utilities and other costs) in the three separation stages for IN-SOL-LQD-VOL-SPC product.

ATPE option in stage-II

The stage-wise categorical (annualized capital, materials, consumables, utilities, labor and others) cost contributions for the ATPE option in stage-II is shown in Figure C.5. In stage-II, the annualized capital costs are very high (~37%) because of the size of the units. This is dependent on the amount of materials handled. The partition coefficient for the product in the top phase (κPT_k) to bottom phase decides the amount of separation agents added as well as the size of the UF membrane technology following the ATPE unit for further separation.

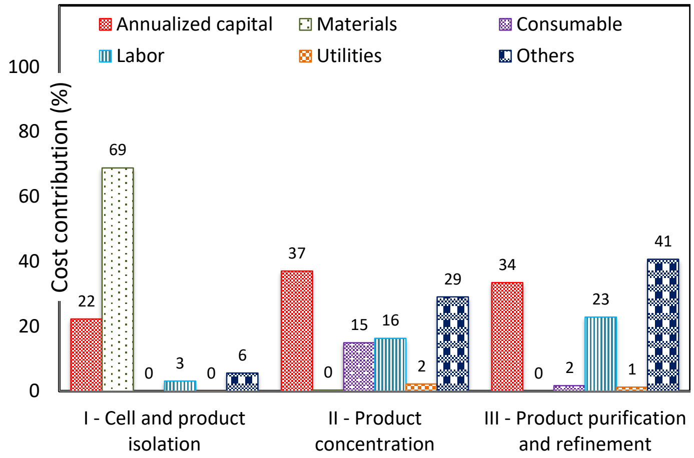

Figure C.5 Cost contribution by different categories (annualized capital, materials, consumables, labor, utilities and other costs) in the three separation stages, when ATPE is the fixed option in stage-II for IN-SOL-LQD-VOL-SPC product.

Extraction option in stage-II

The stage-wise categorical (annualized capital, materials, consumables, utilities, labor and others) cost contributions for the extraction option in stage-II is shown in Figure C.6. In stage-II, the material costs are very high (~96%) because of the extraction solvent used for product recovery. The amount of solvent

required is dependent on the partition coefficient (κP_k), solubility of solvent in water (ψ_{Sol-W}) or the raffinate phase, and the cost of solvent (π_{Sol}).

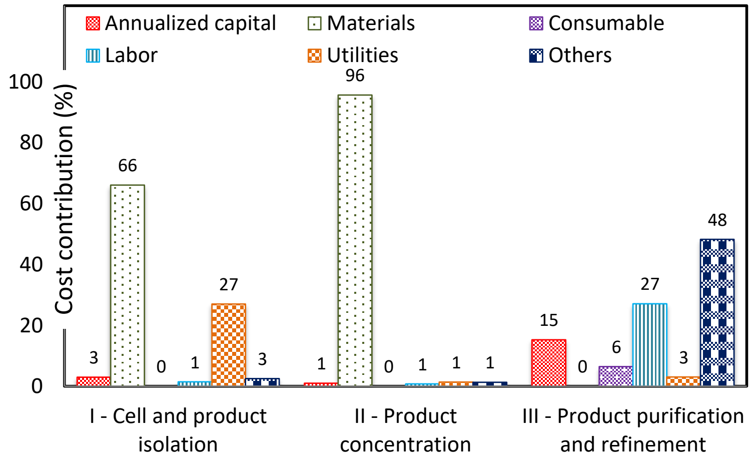

Figure C.6 Cost contribution by different categories (annualized capital, materials, consumables, labor, utilities and other costs) in the three separation stages, when extraction (Ext) is the fixed option in stage-II for IN-SOL-LQD-VOL-SPC product.

C.5.2 Plots for distillation parameters

The change in heat of vaporization does not have a significant effect on the overall process cost, however stage-II cost can increase by 6% if the heat of vaporization increases from 700 to 2200 KJ/kg.

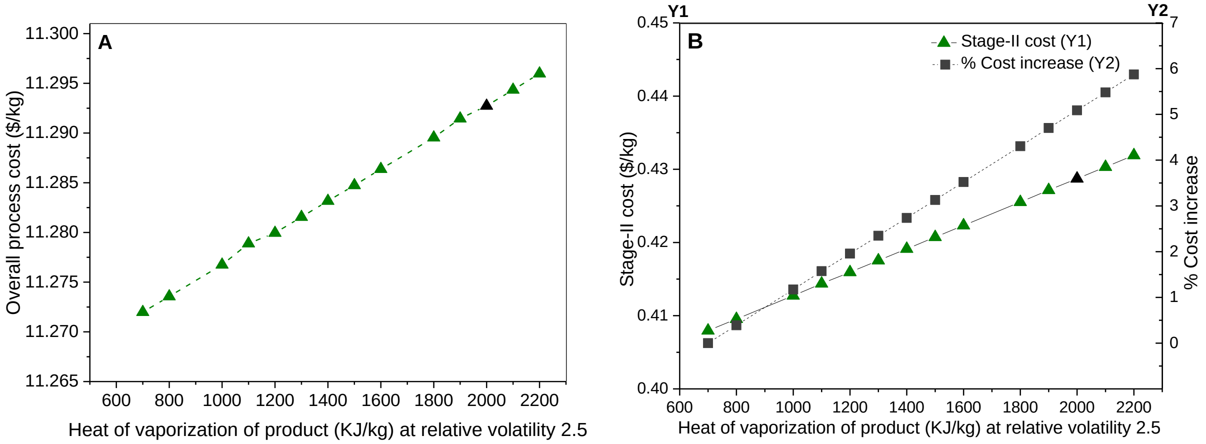

Figure C.7 Variation in (A) overall process cost (B) stage-II cost (Y1) and % cost increase (Y2) as a function of heat of vaporization of the product.

C.5.3 Plots for extraction parameters

For extraction unit the solubility and solvent costs have a significant effect on the overall cost however, the partition coefficient does not affect the cost significantly. The lower the values of solubility and solvent costs, the lower is the overall process cost.

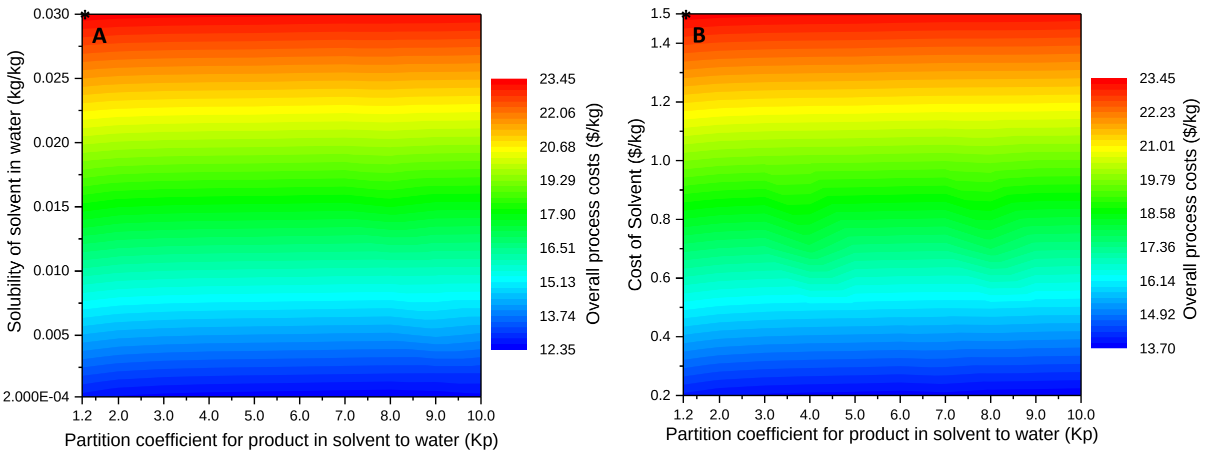

Figure C.8 Variation in (A) partition coefficient of solvent versus its solubility in water, and (B) partition coefficient of solvent versus cost of solvent. The nominal values assumed for the three parameters (Kp - 1.2, Cost of solvent - 1.5 $/kg and Solubility of solvent in water - 0.03 kg/kg) and the corresponding overall process cost is shown by the black marker.

C.5.4 Information for crystallization cost

For cost estimation, we choose the product containing stream leaving the extraction unit. Using SuperPro Designer v8.5 we simulate a cooling crystallization unit. The information for the input conditions and crystallization parameters is provided in Table C.5. The crystallization process cost is 8 $/kg. Thus, on adding the cost of feed (1.91 $/kg), stage-I (9.81 $/kg), stage-II (11.29 $/kg) and crystallization (8 $/kg), the overall process cost is 31 $/kg (Kp-1.2, Solubility-0.03 kg/kg, Cost of solvent-1.2 $/kg). Thus, the percentage contribution by crystallization is ~26%.

Table C.5. Parameters and input conditions in crystallization:

Parameter	Nominal value	Unit
Heat of crystallization	70,420	Kcal/kg
Inlet stream temperature	70	^{0}C
Crystallization temperature	15	^{0}C
Product in stream	700	kg/h
Other components (water)	200	kg/h
Specific power	0.1	KW/m^3
Chilled water inlet temperature	5	^{0}C
Chilled water outlet temperature	10	^{0}C
Crystallization product yield	95	%
Solubility at 70^{0}C	3.8	g/g
Solubility at 15^{0}C	1	g/g

References

1. Choi J, Lee SY. Efficient and economical recovery of poly(3-hydroxybutyrate) from recombinant Escherichia coli by simple digestion with chemicals. Biotechnol Bioeng. 1999;62:546–53.

2. Choi J, Lee SY. Process analysis and economic evaluation for Poly(3-hydroxybutyrate) production by fermentation. Bioprocess Eng. 1997;17:335–42.

3. Frey KM, Oppermann-Sanio FB, Schmidt H, Steinbüchel A. Technical-Scale Production of Cyanophycin with Recombinant Strains of Escherichia coli. Appl Environ Microbiol. 2002;68:3377–84.

4. Mooibroek H, Oosterhuis N, Giuseppin M, Toonen M, Franssen H, Scott E, et al. Assessment of technological options and economical feasibility for cyanophycin biopolymer and high-value amino acid production. Appl Microbiol Biotechnol. 2007;77:257–67.

5. Ruiz-Ruiz F, Benavides J, Rito-Palomares M. Scaling-up of a B-phycoerythrin production and purification bioprocess involving aqueous two-phase systems: Practical experiences. Process Biochem. 2013;48:738–45.

6. Cuellar-Bermudez SP, Aguilar-Hernandez I, Cardenas-Chavez DL, Ornelas-Soto N, Romero-Ogawa MA, Parra-Saldivar R. Extraction and purification of high-value metabolites from microalgae: essential lipids, astaxanthin and phycobiliproteins. Microb Biotechnol. 2015;8:190–209.

7. Machmudah S, Shotipruk A, Goto M, Sasaki M, Hirose T. Extraction of Astaxanthin from *Haematococcus p luvialis* Using Supercritical CO_2 and Ethanol as Entrainer. Ind Eng Chem Res. 2006;45:3652–7.

8. Sarada R, Vidhyavathi R, Usha D, Ravishankar GA. An efficient method for extraction of astaxanthin from green alga Haematococcus pluvialis. J Agric Food Chem. 2006;54:7585–8.

9. Yenkie KM, Wu W, Clark RL, Pfleger BF, Root TW, Maravelias CT. A roadmap for the synthesis of separation networks for the recovery of bio-based chemicals: Matching biological and process feasibility. Biotechnol Adv. 2016. doi:10.1016/j.biotechadv.2016.10.003.

10. Kokossis AC, Tsakalova M, Pyrgakis K. Design of integrated biorefineries. Comput Chem Eng. 2015;81:40–56.

11. Wu W, Henao CA, Maravelias CT. A superstructure representation, generation, and modeling framework for chemical process synthesis. AIChE J. 2016. http://onlinelibrary.wiley.com/doi/10.1002/aic.15300/abstract. Accessed 3 Aug 2016.

12. Yeomans H, Grossmann IE. A systematic modeling framework of superstructure optimization in process synthesis. Comput Chem Eng. 1999;23:709–31.

13. Kim J, Sen SM, Maravelias CT. An optimization-based assessment framework for biomass-to-fuel conversion strategies. Energy Environ Sci. 2013;6:1093.
